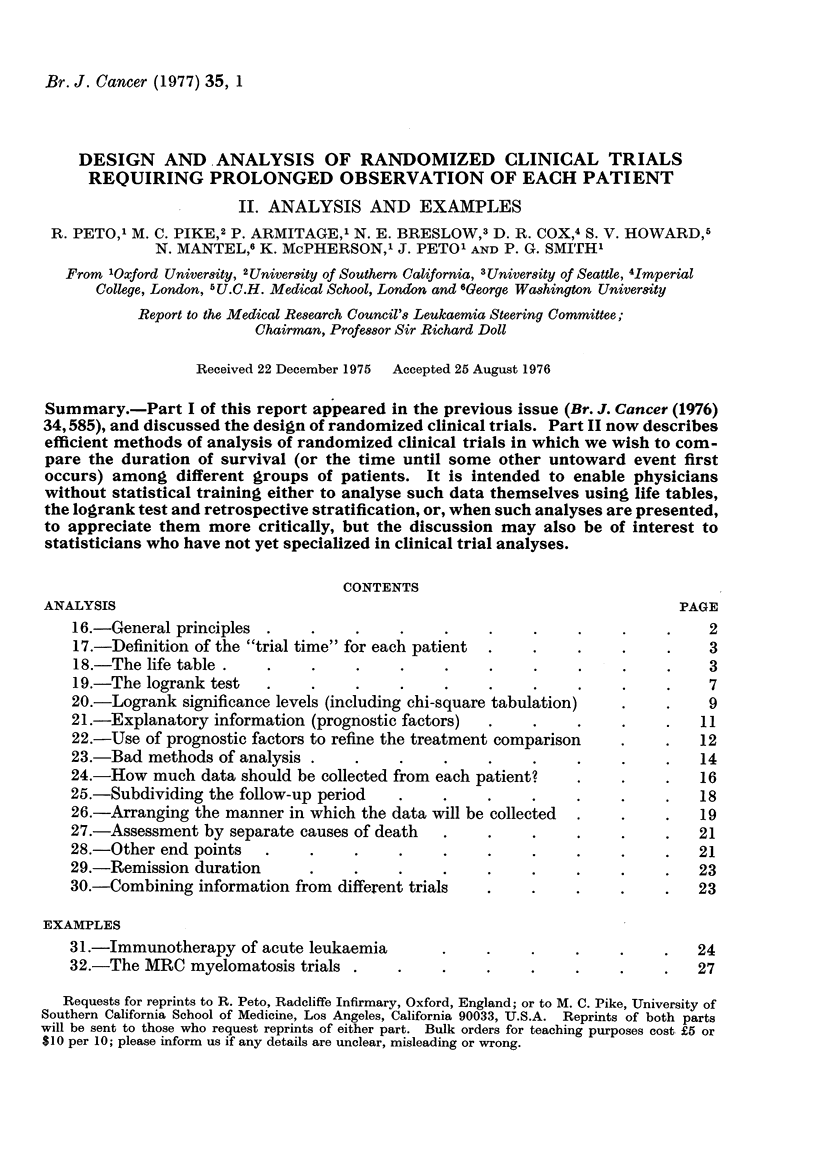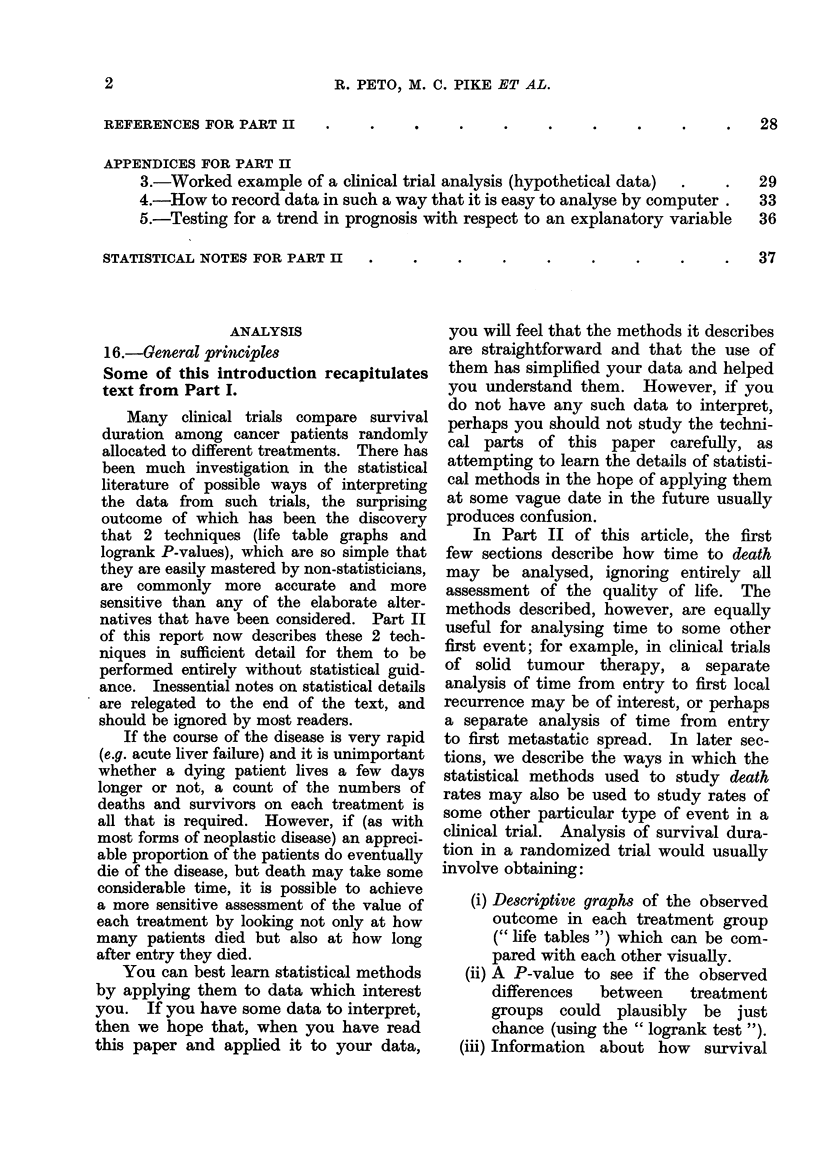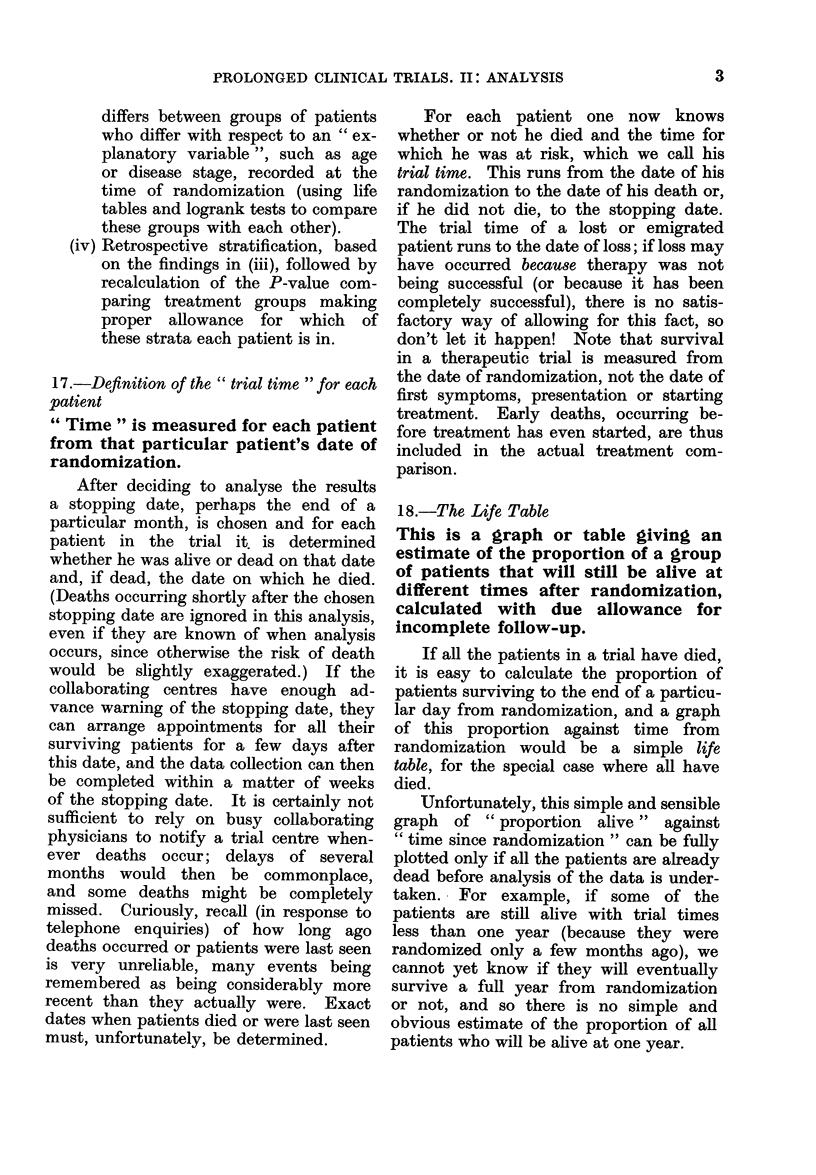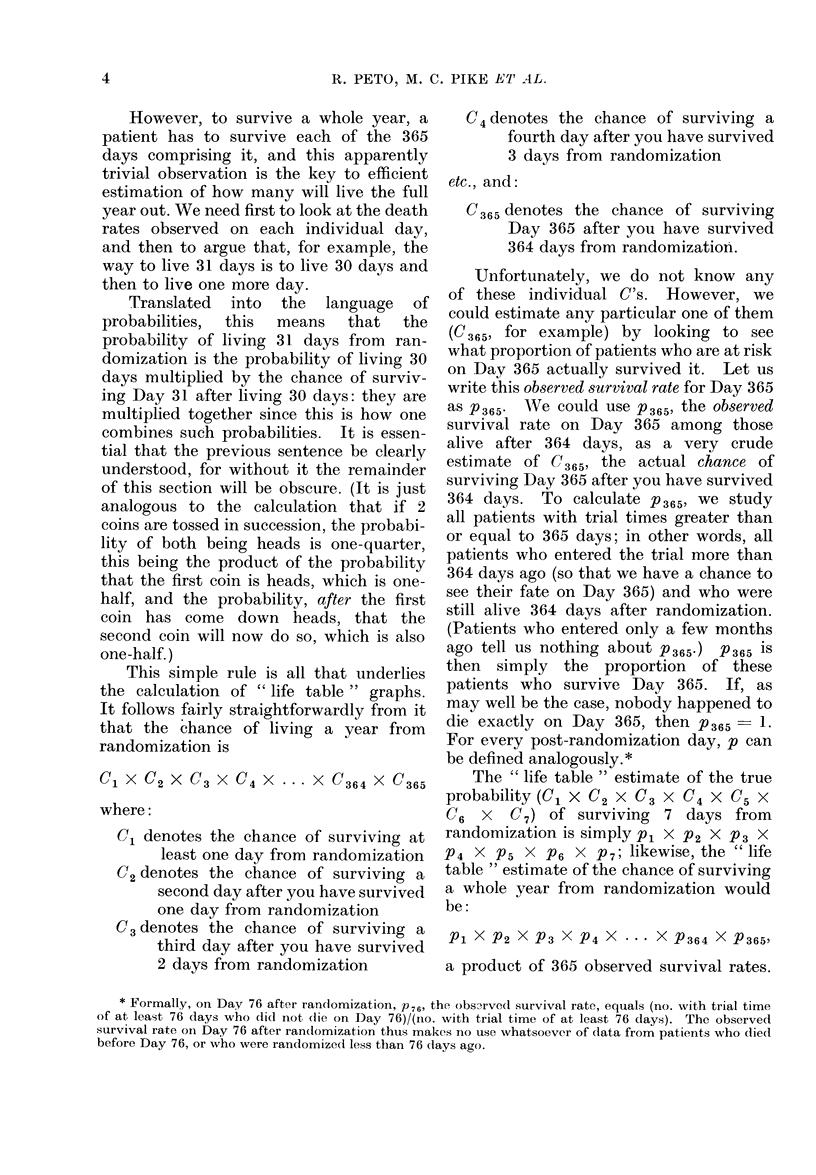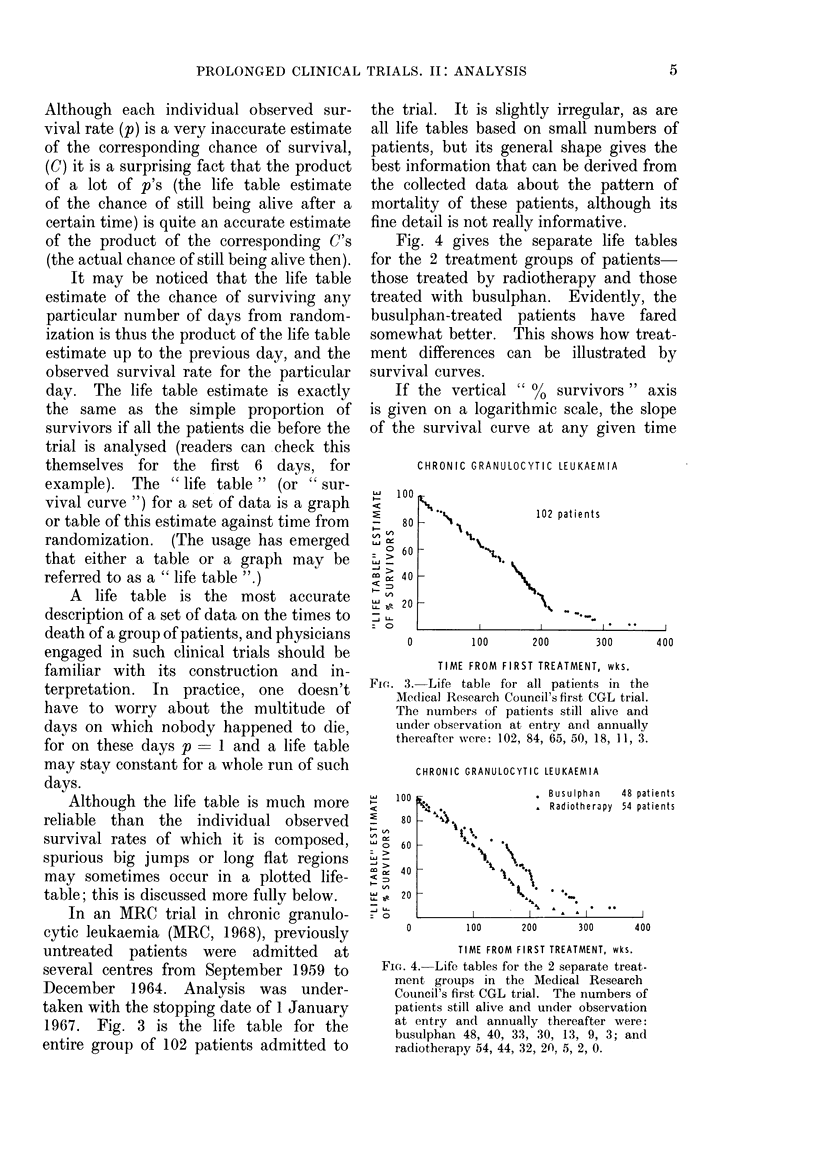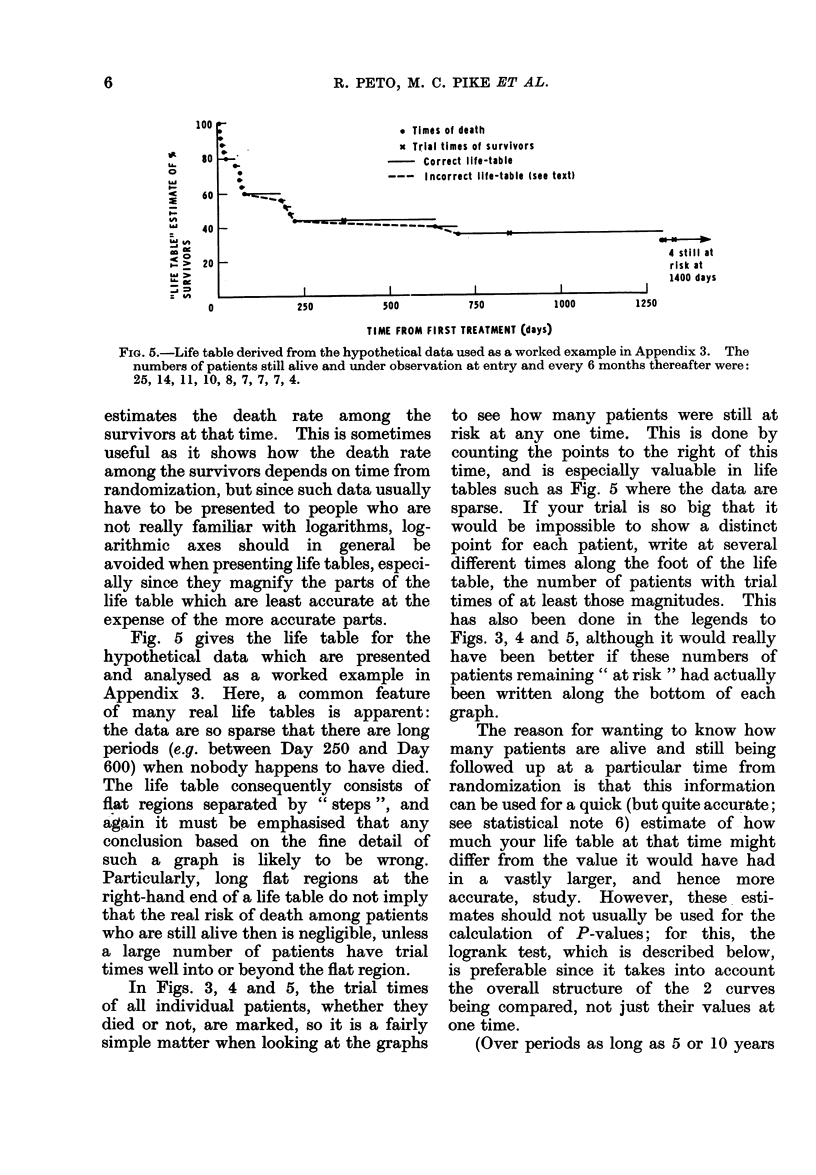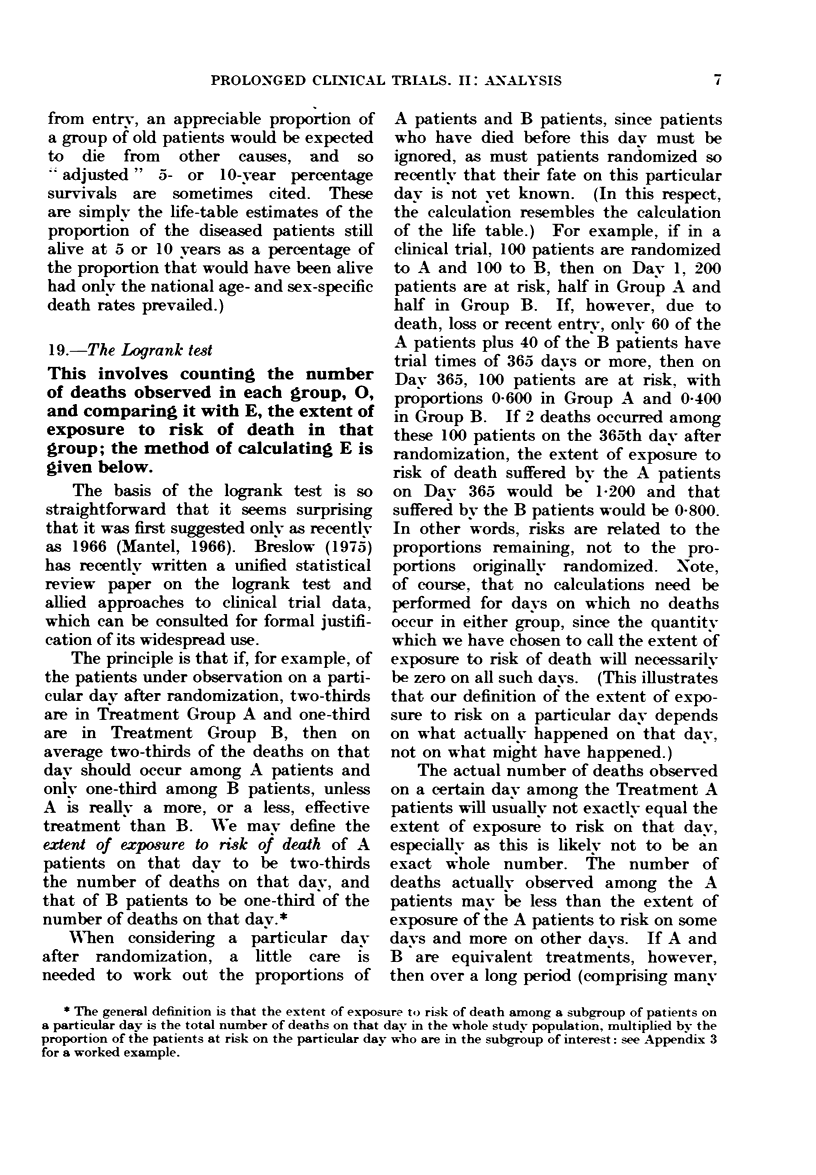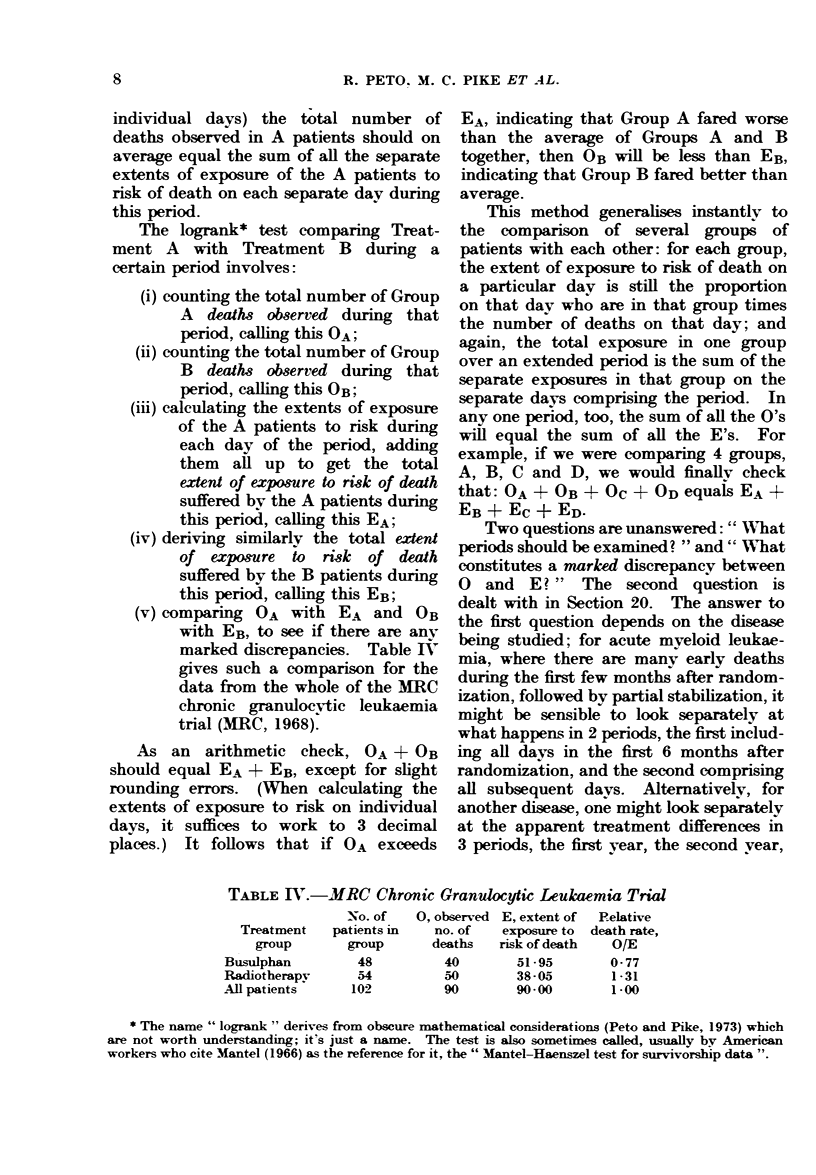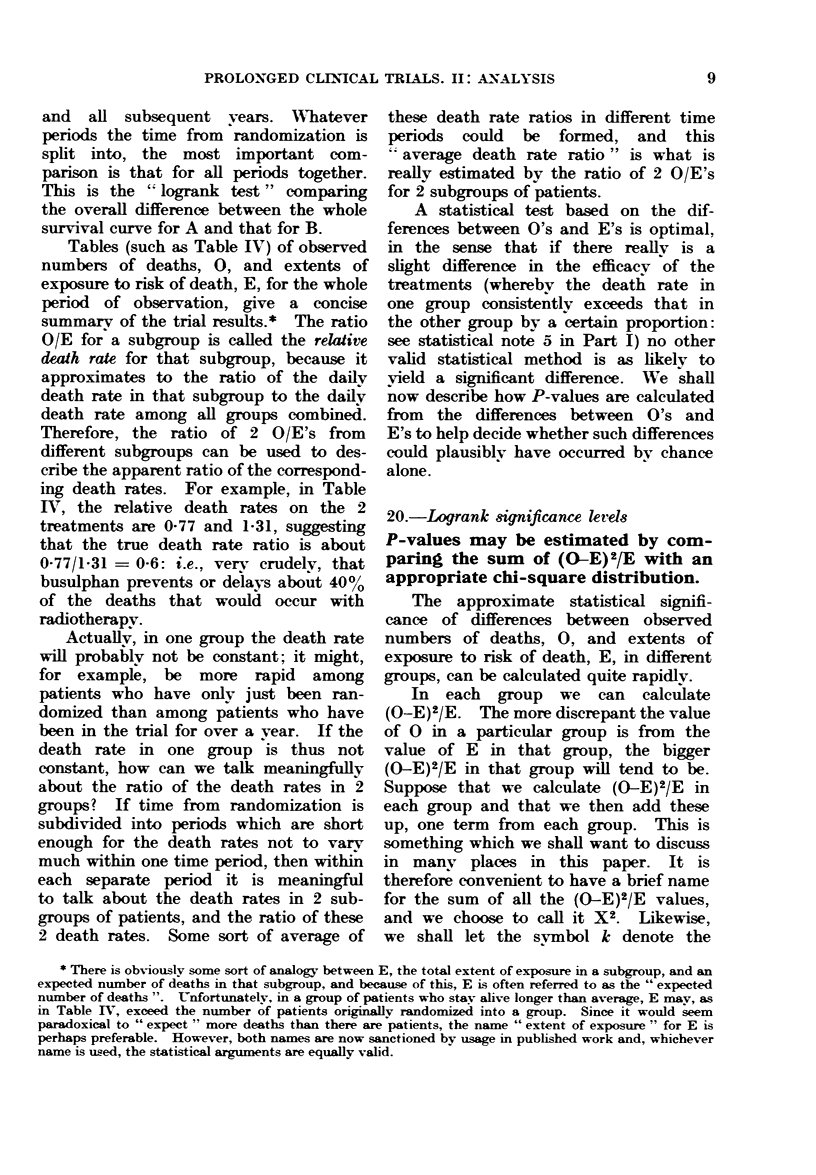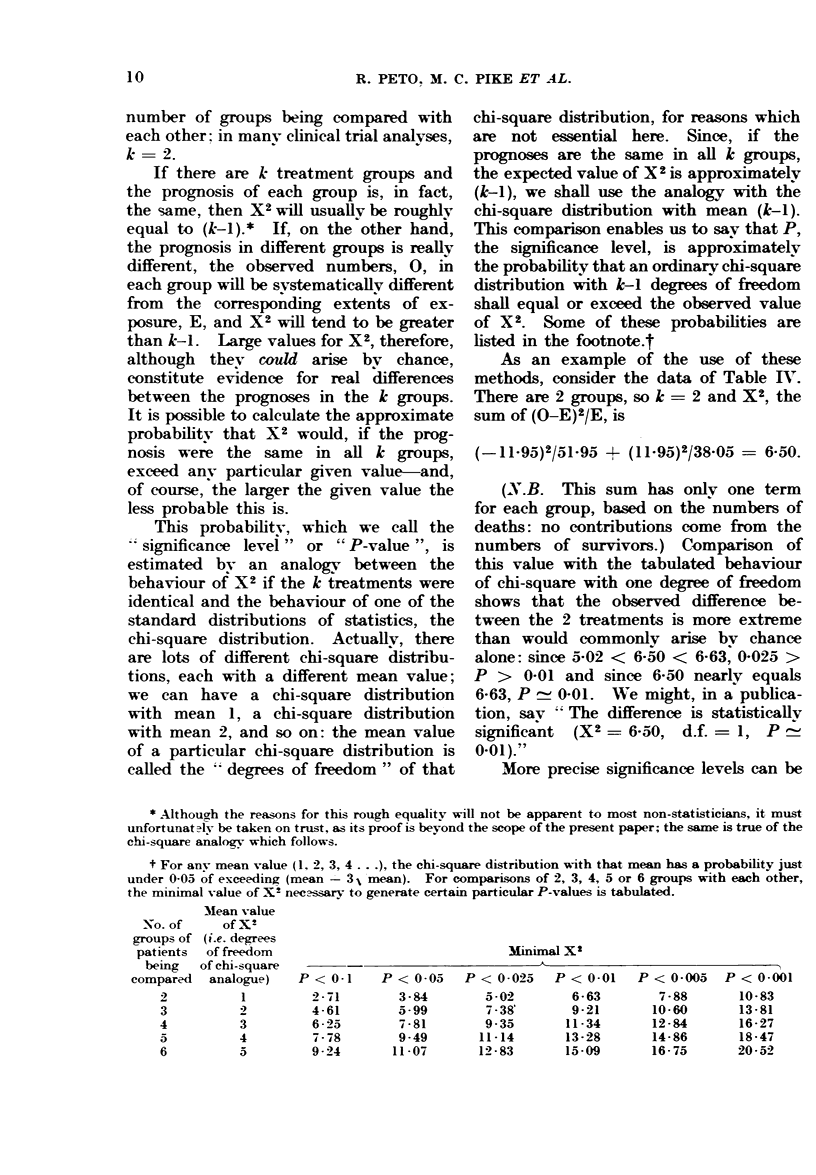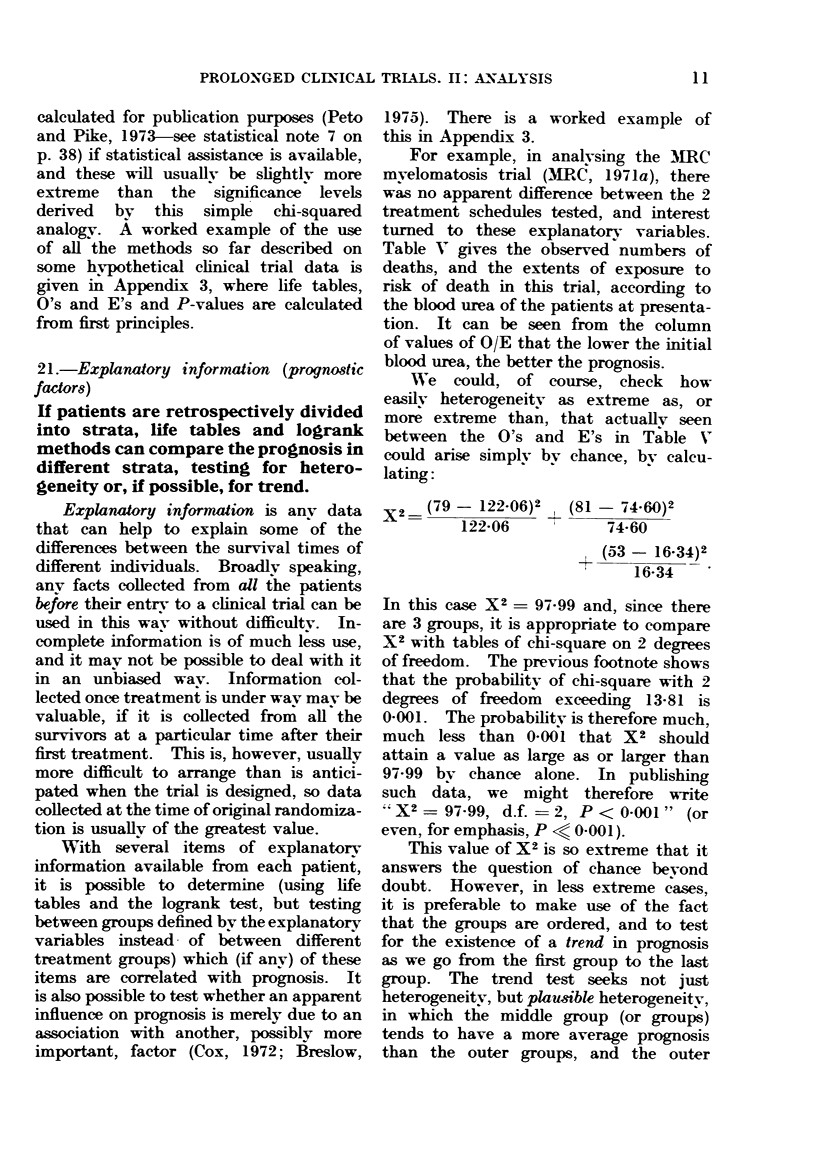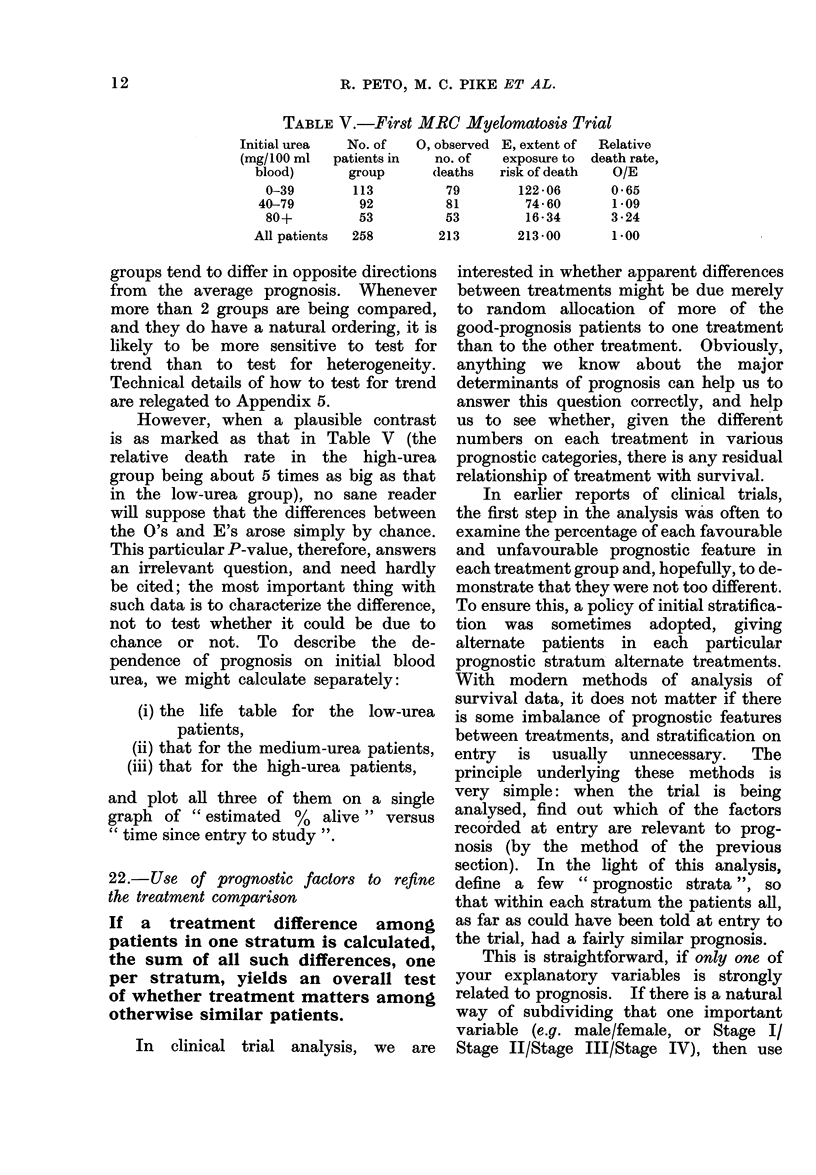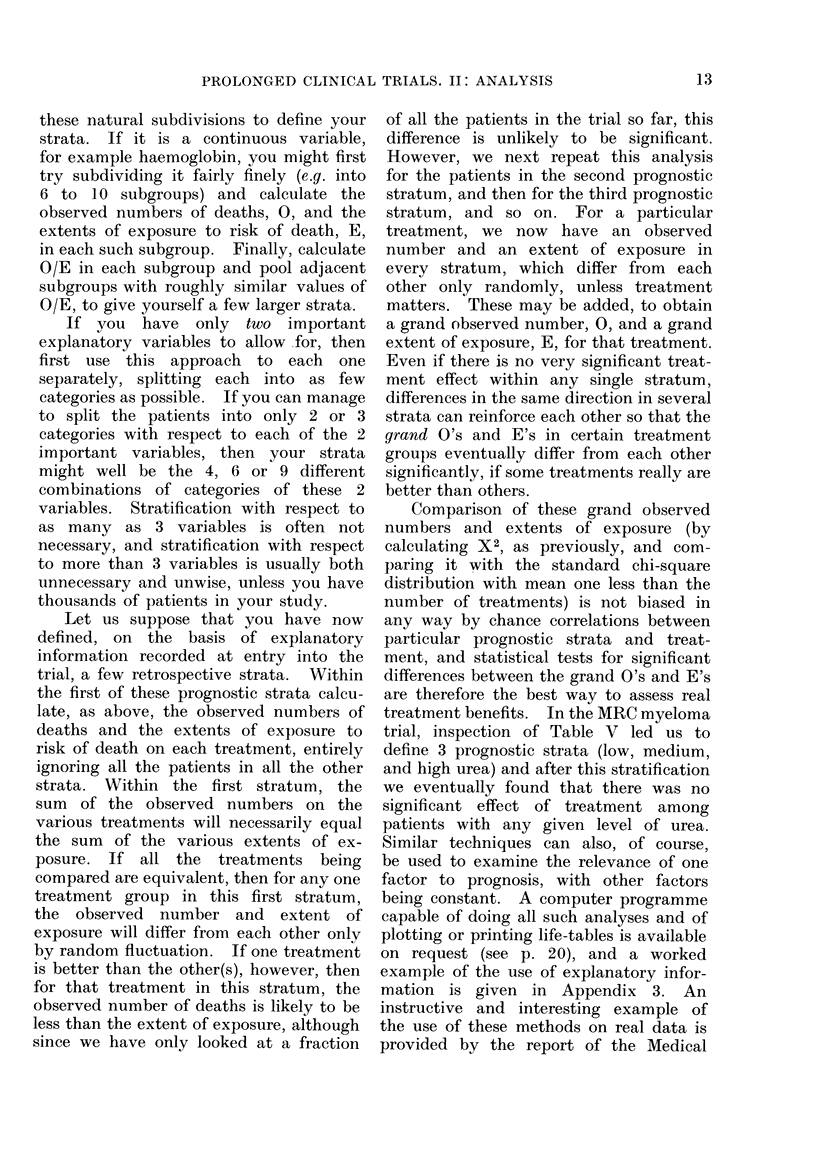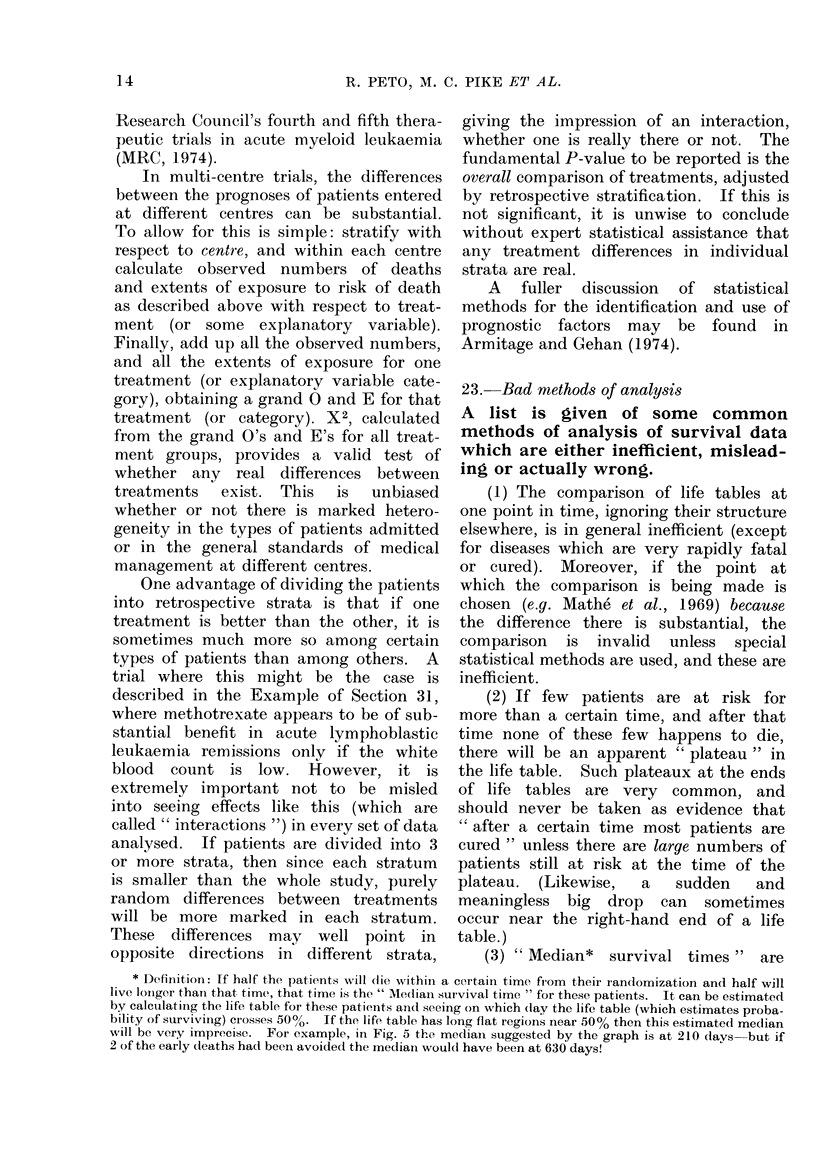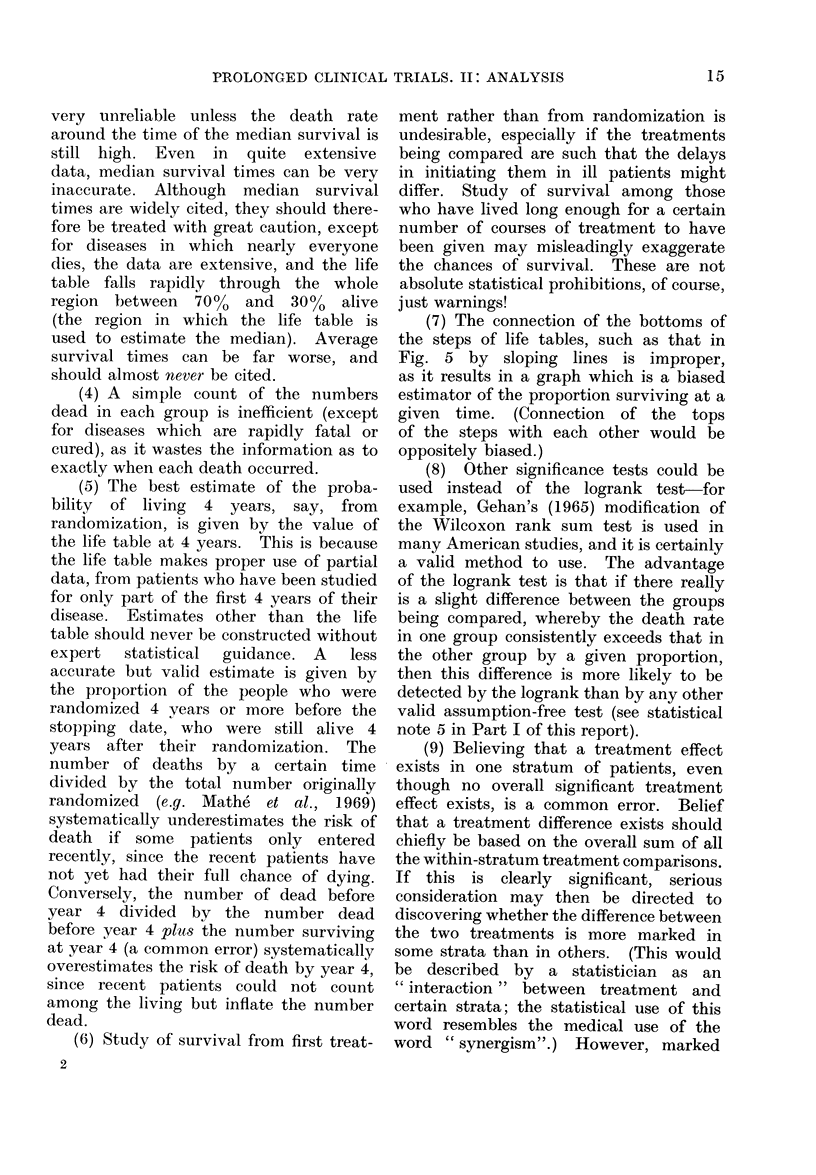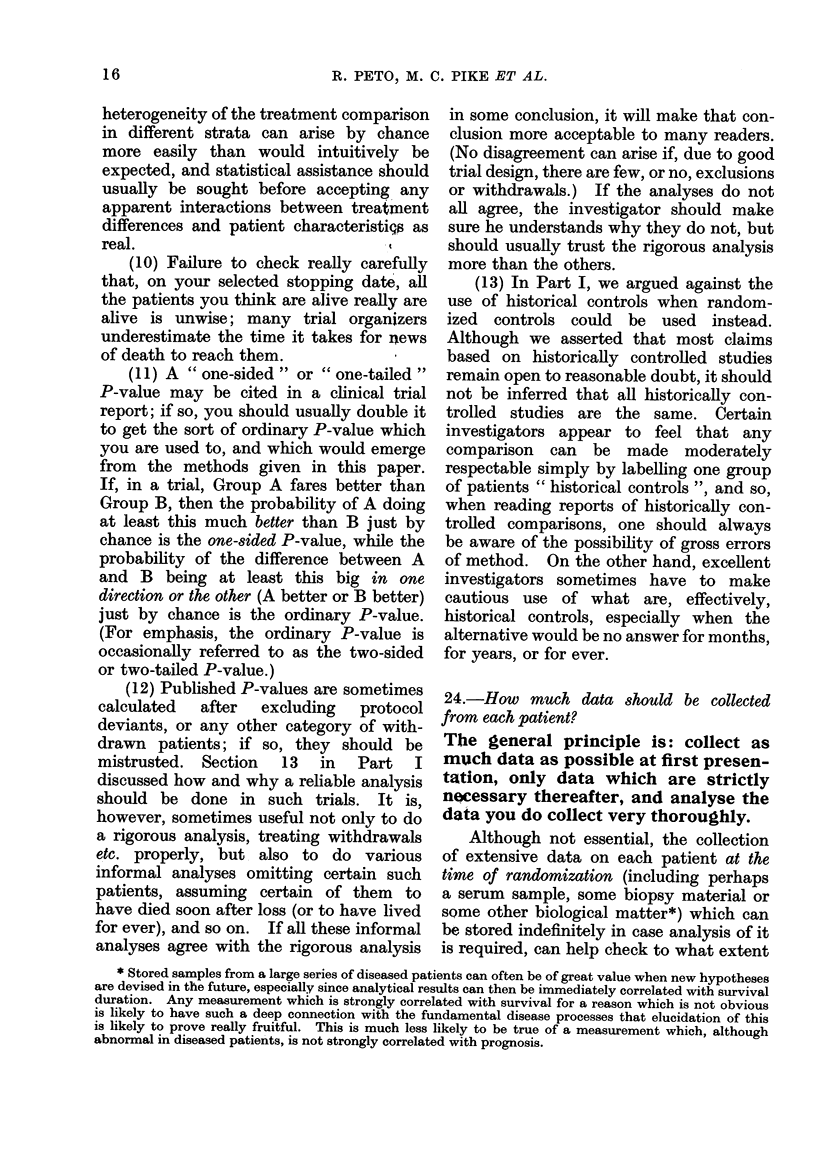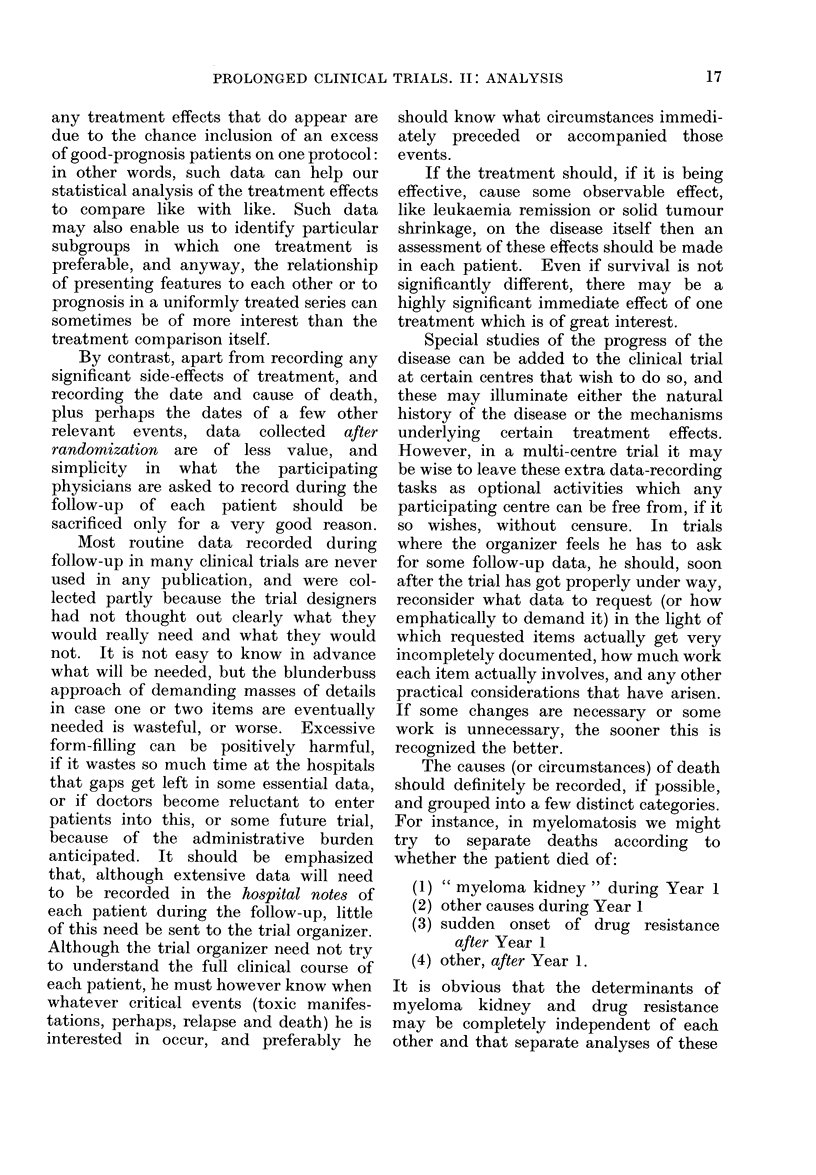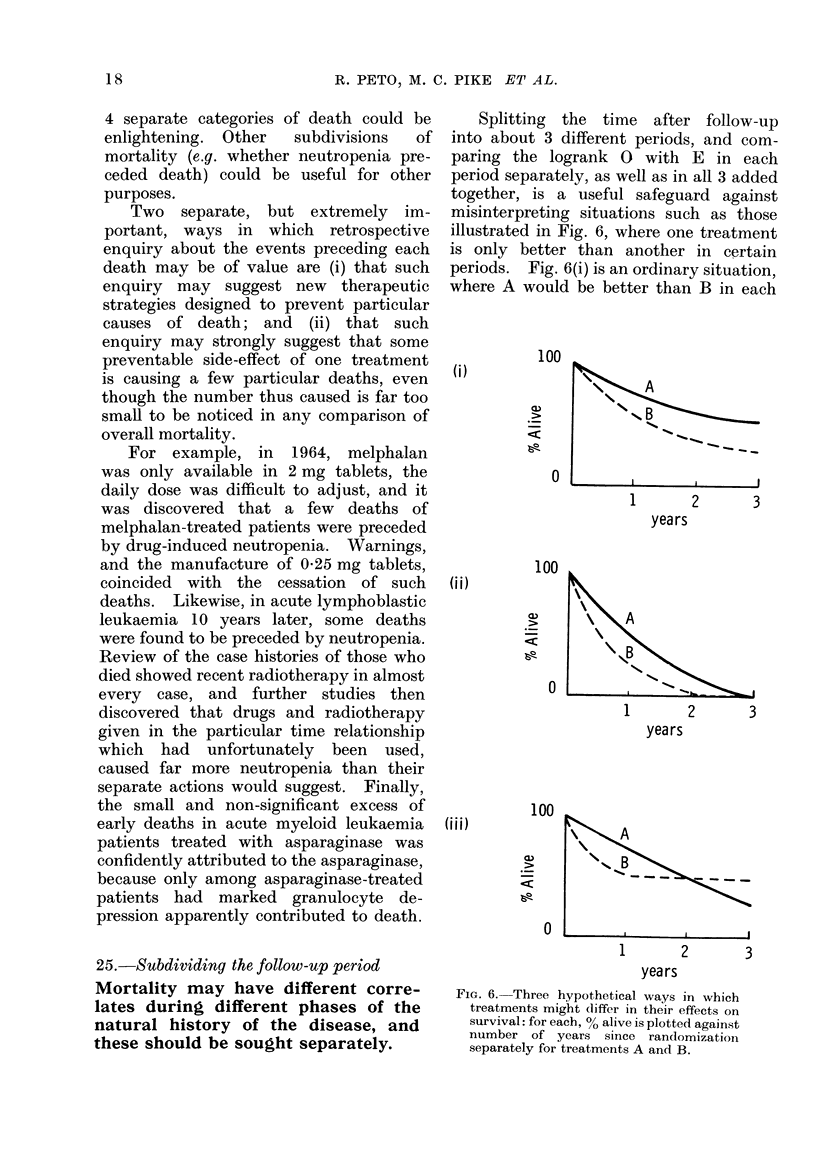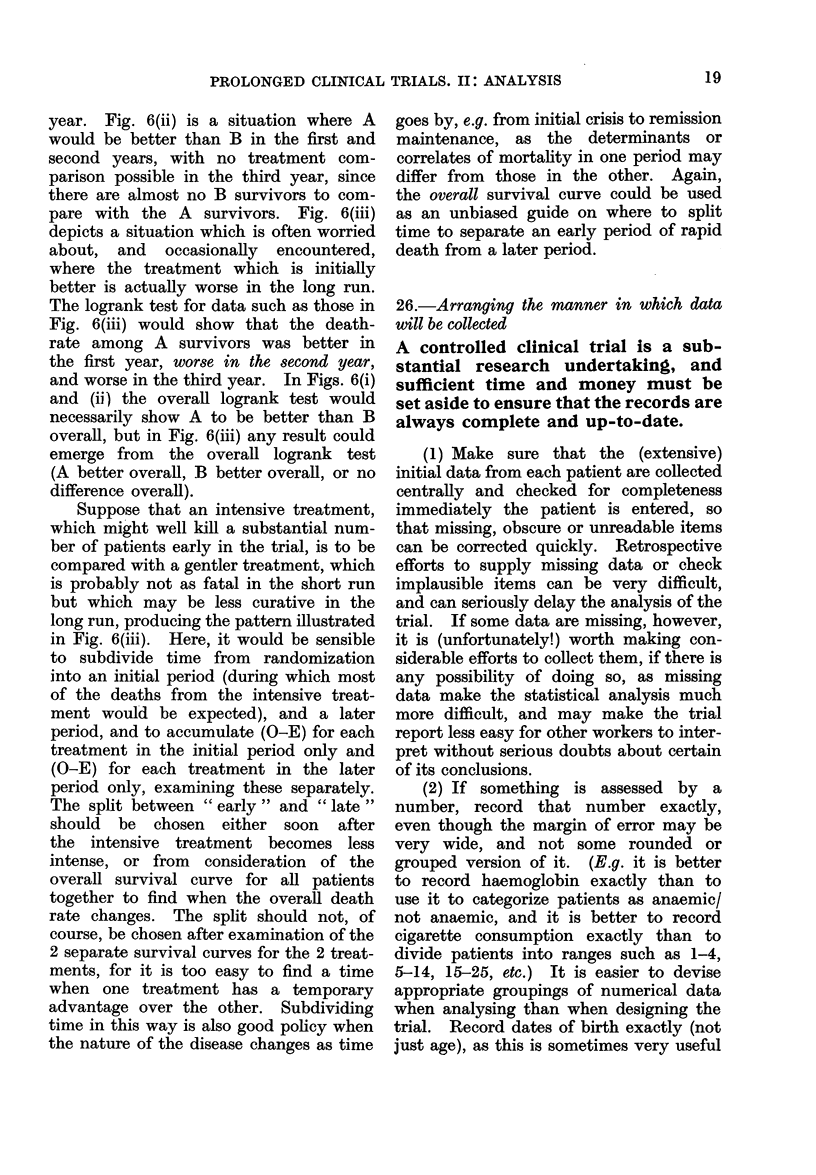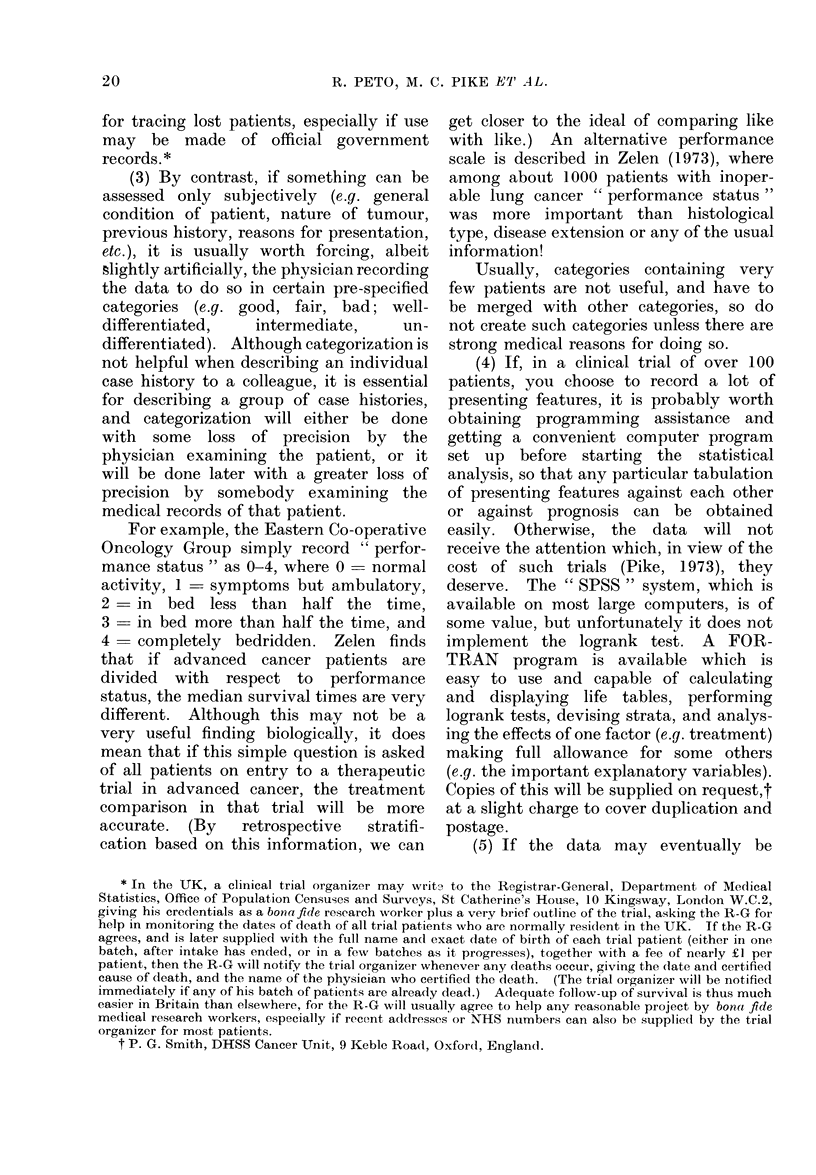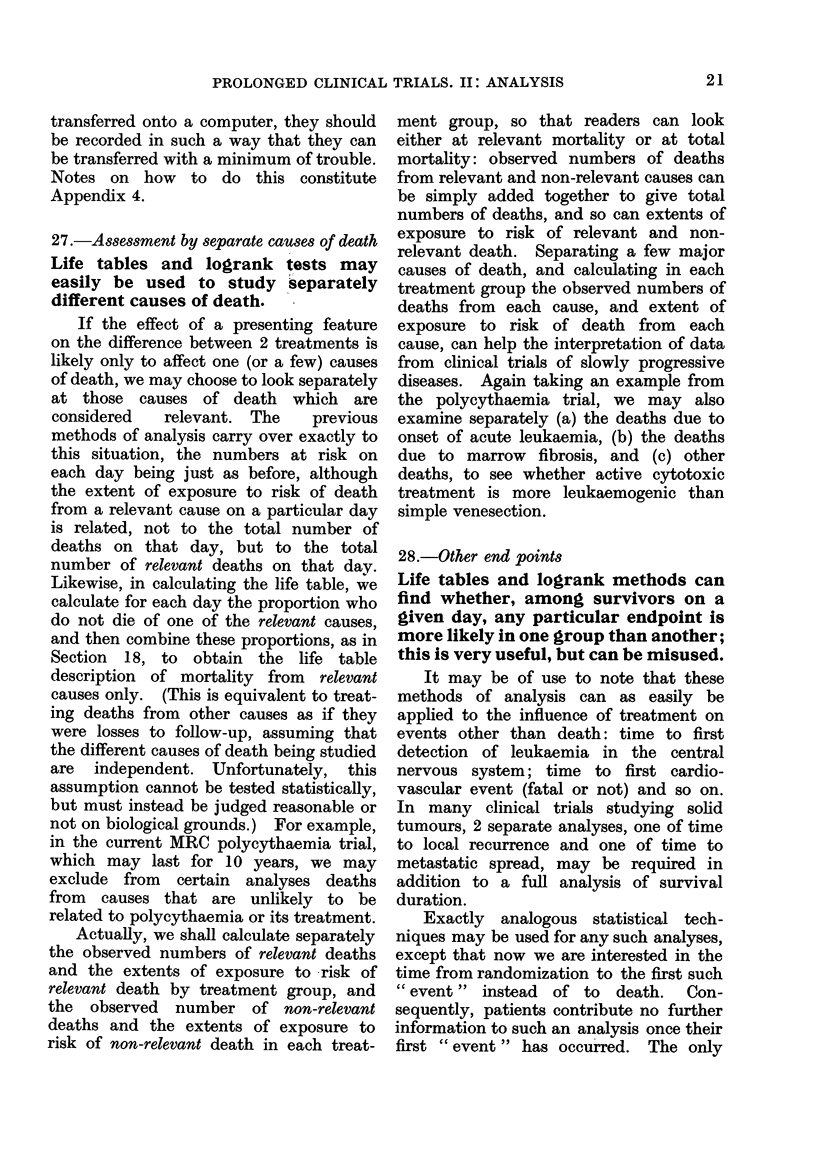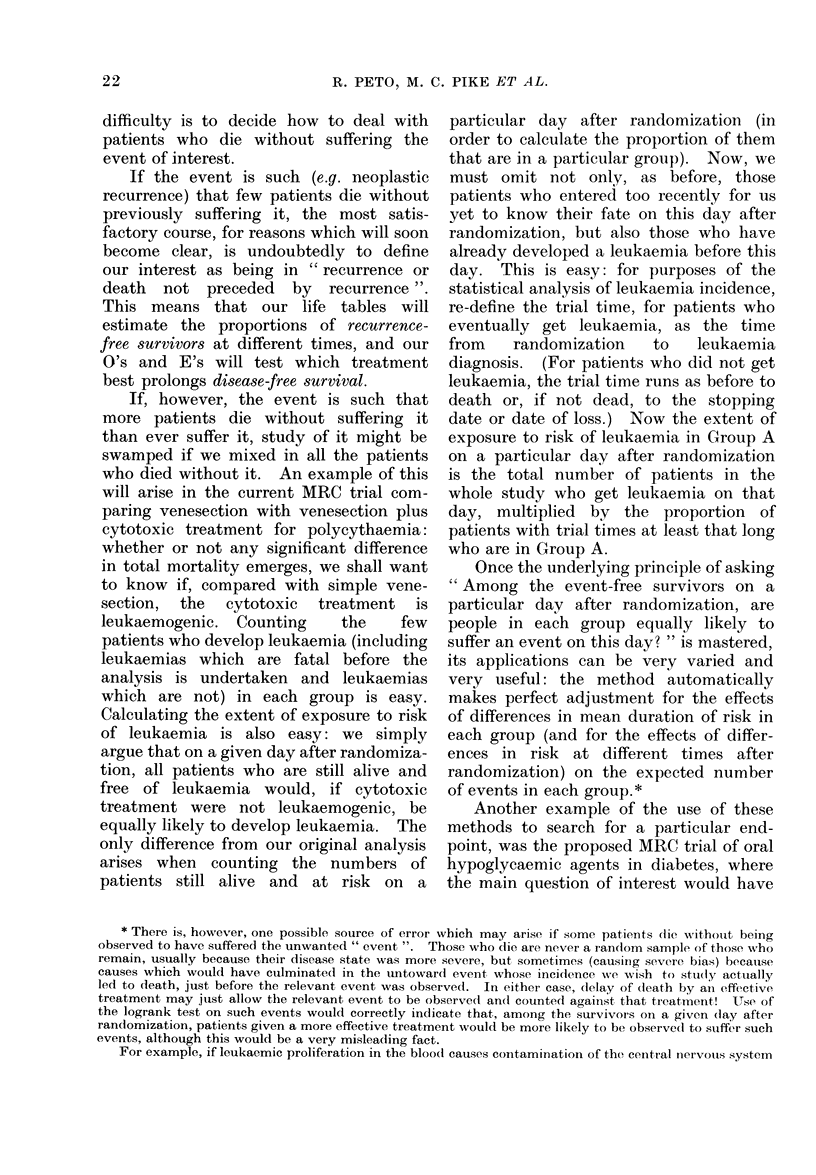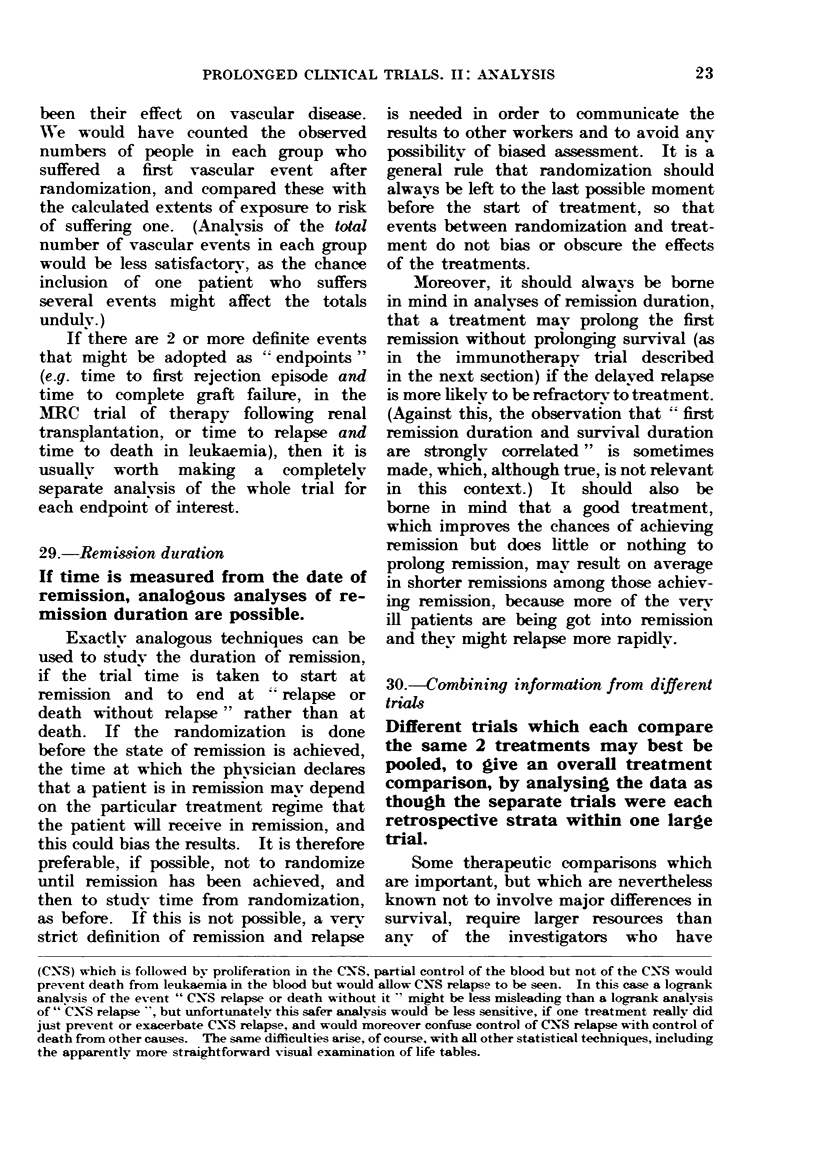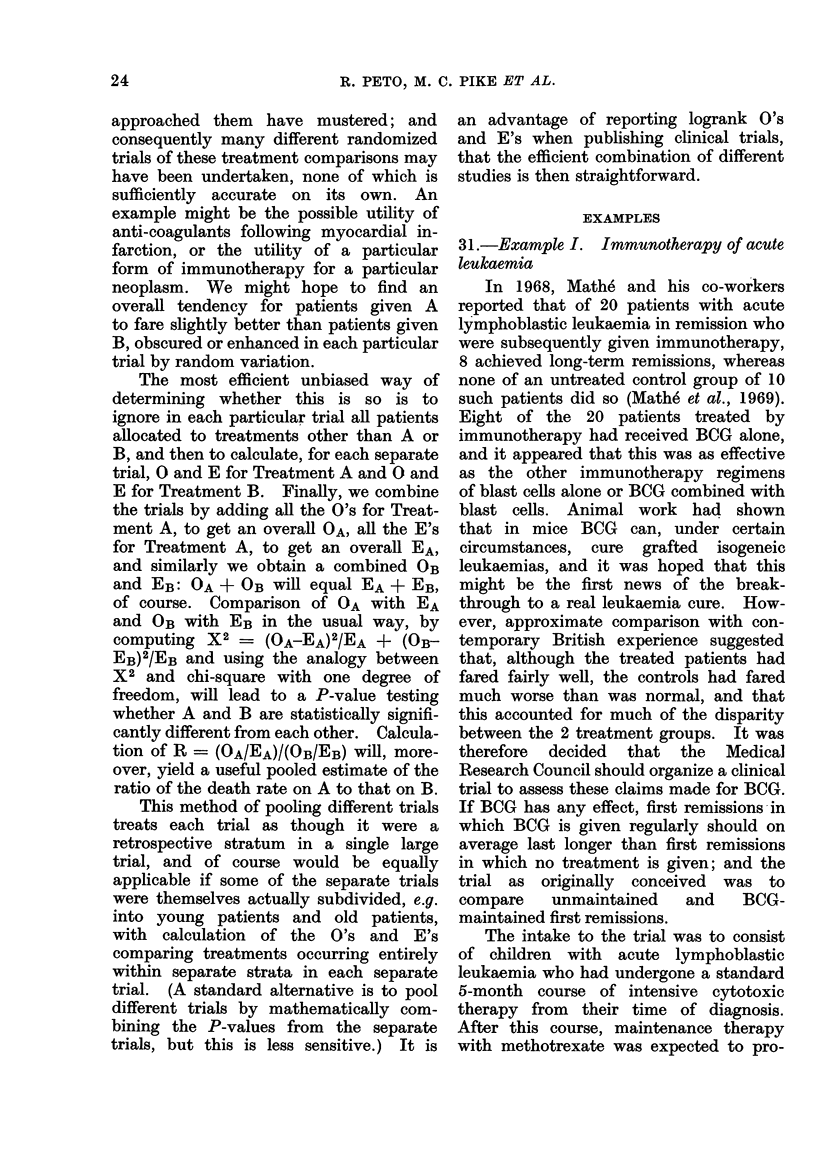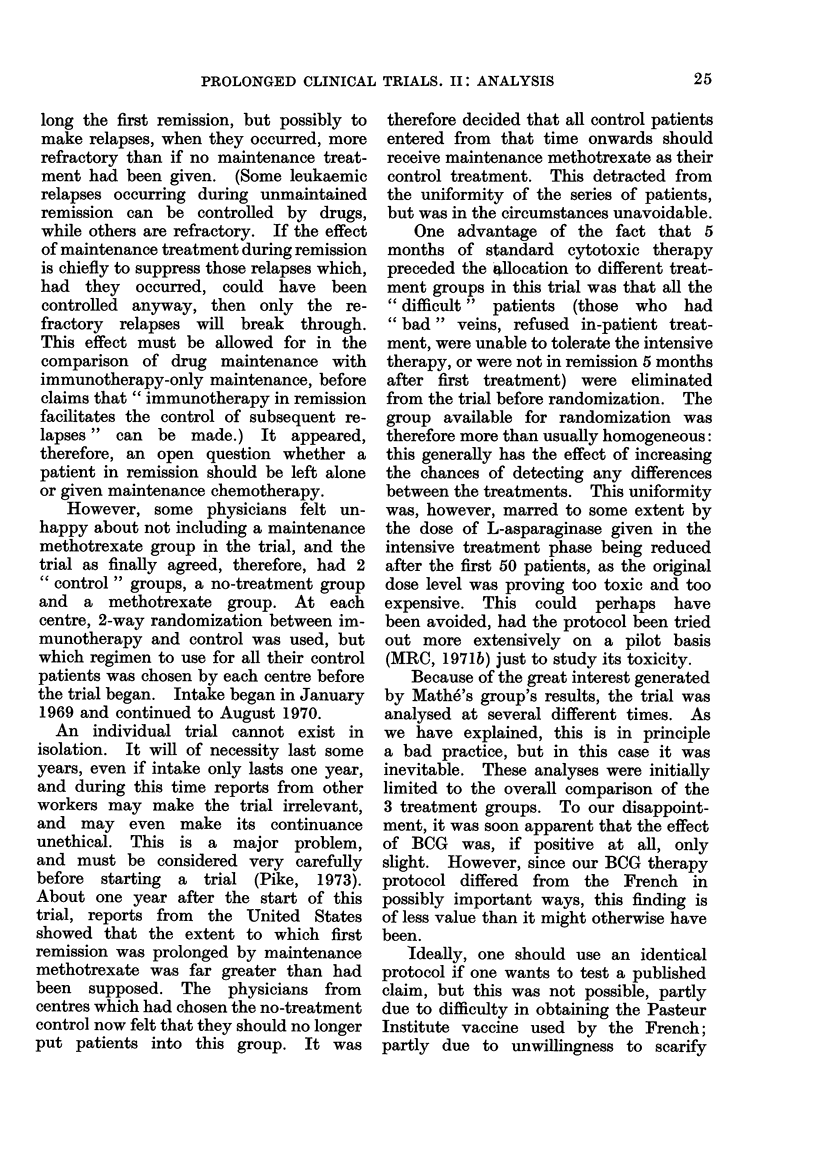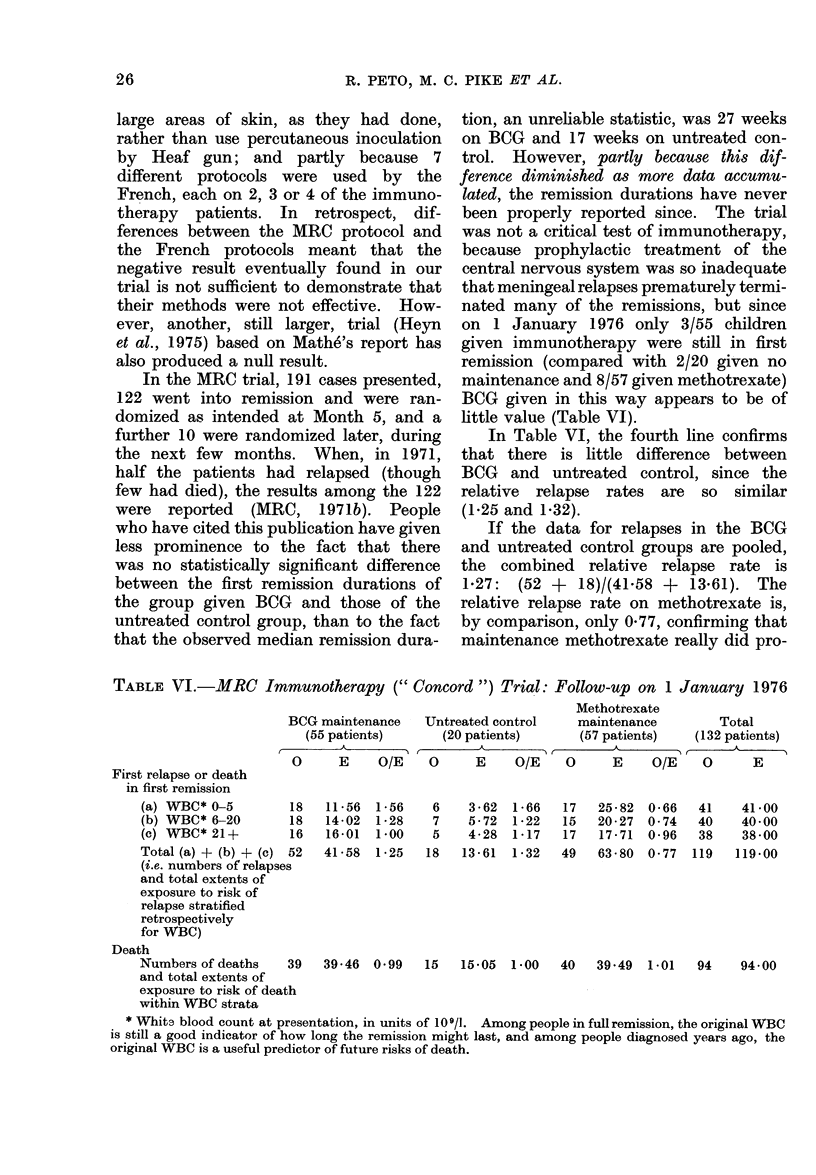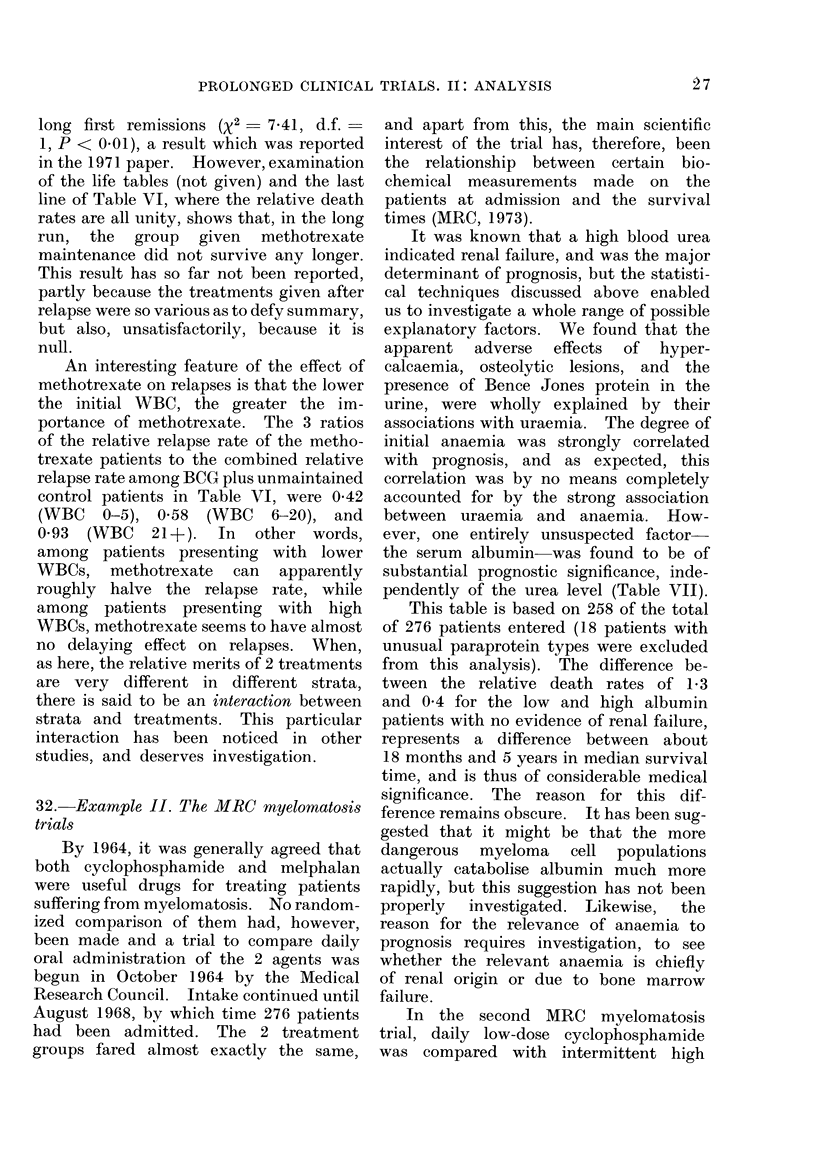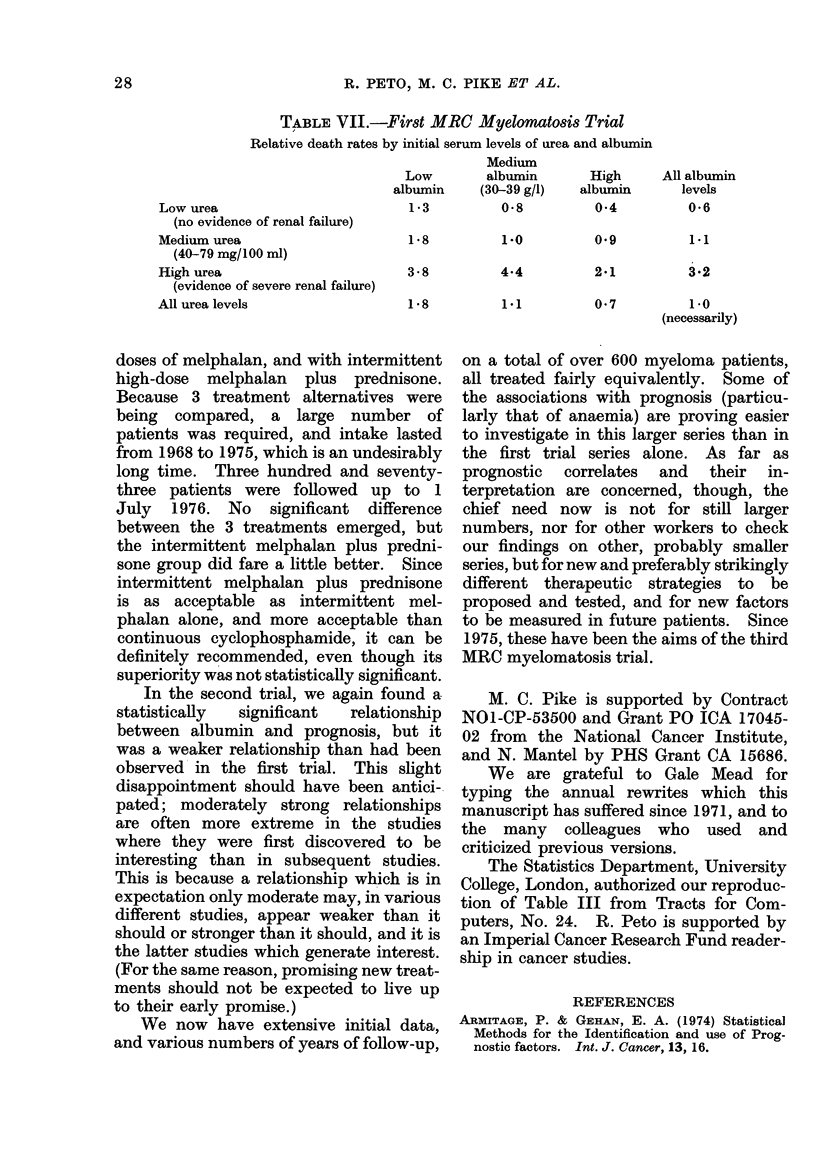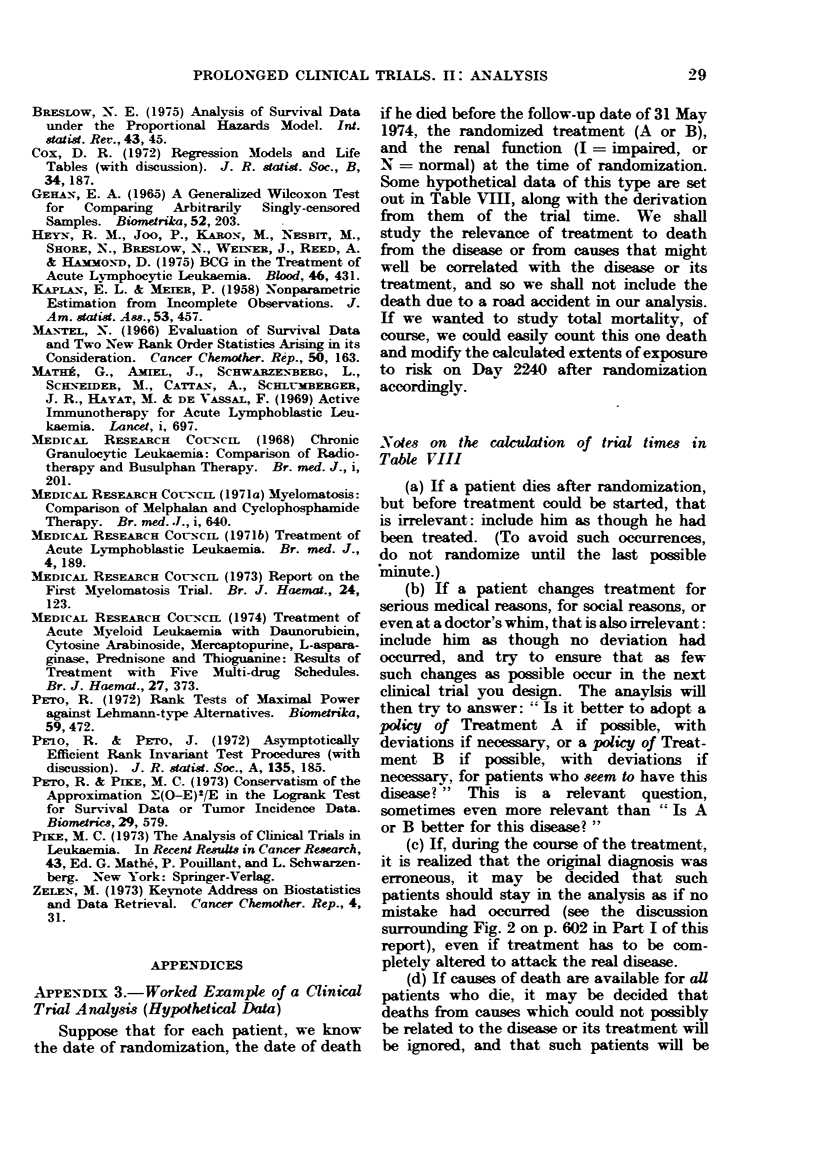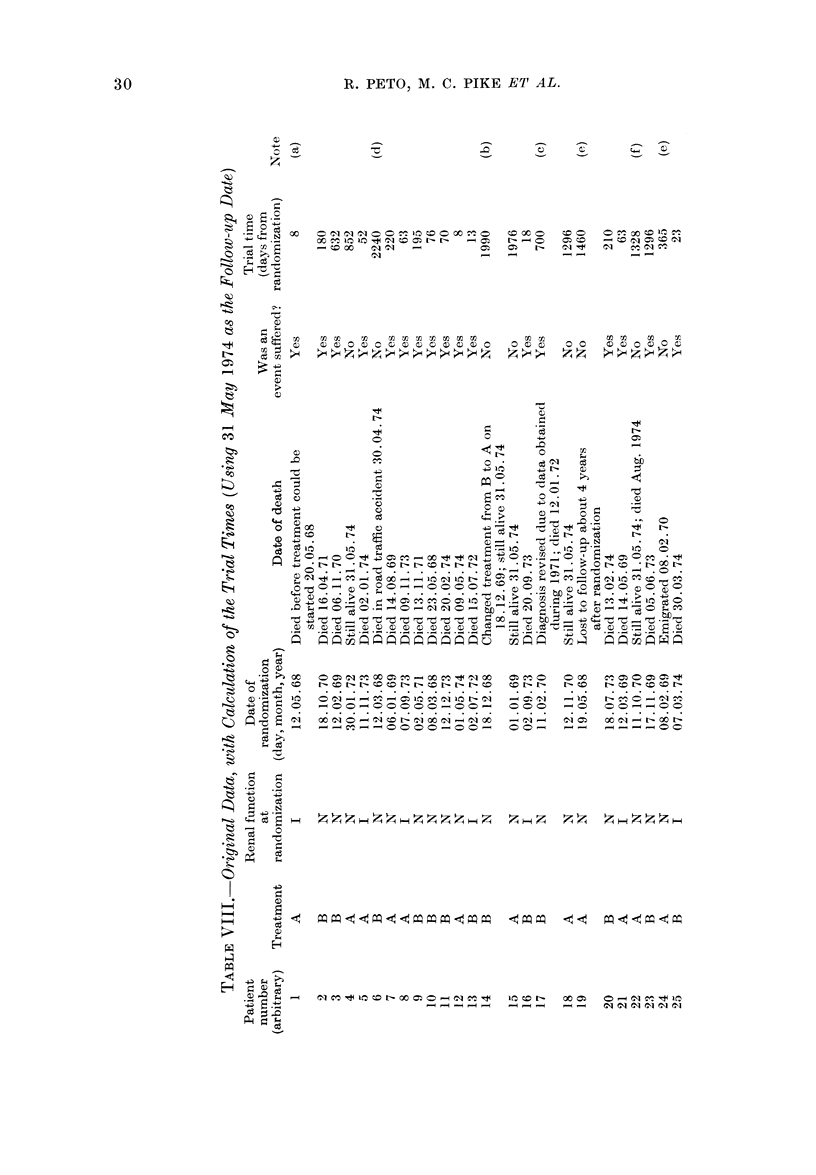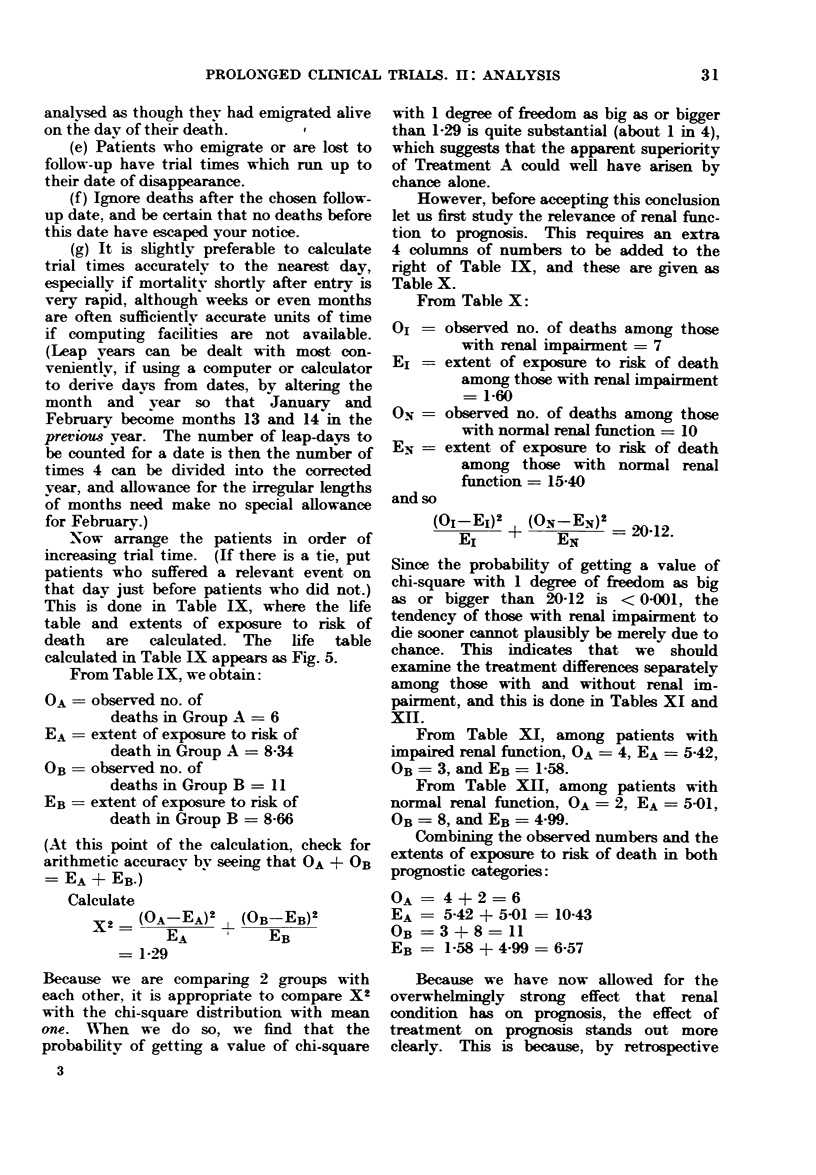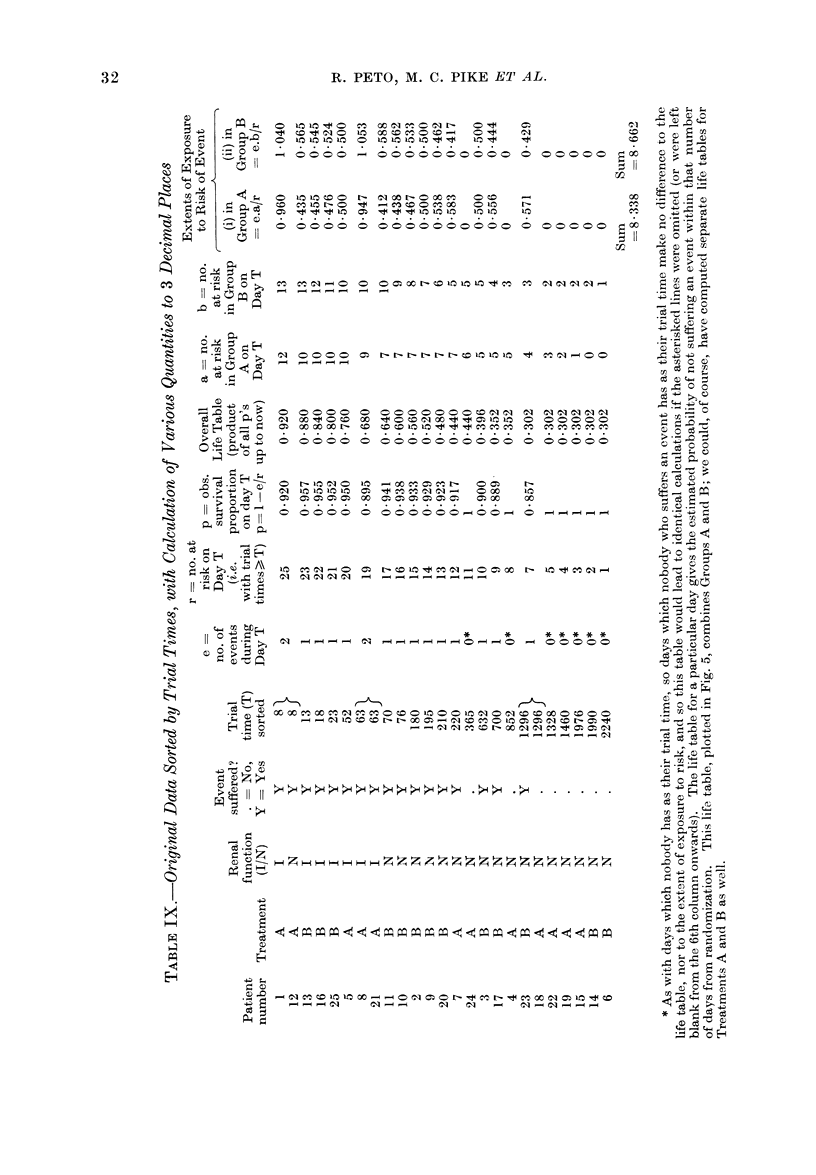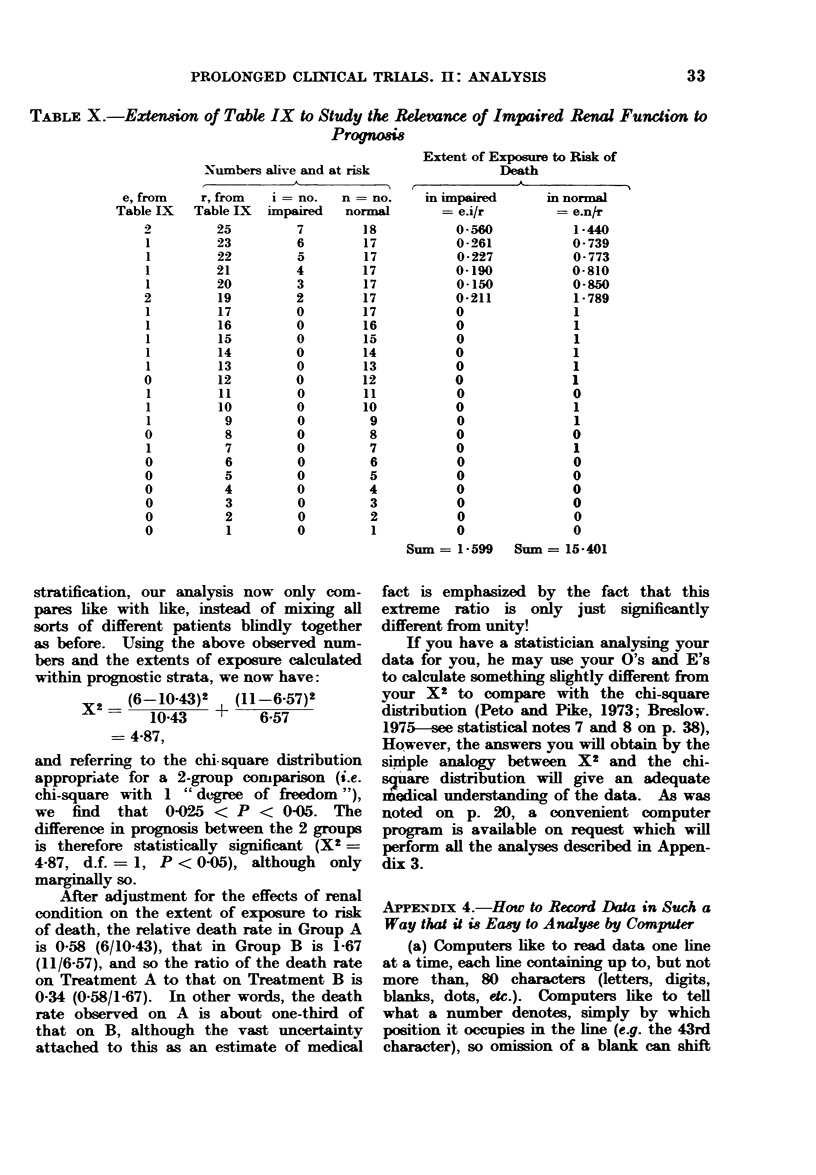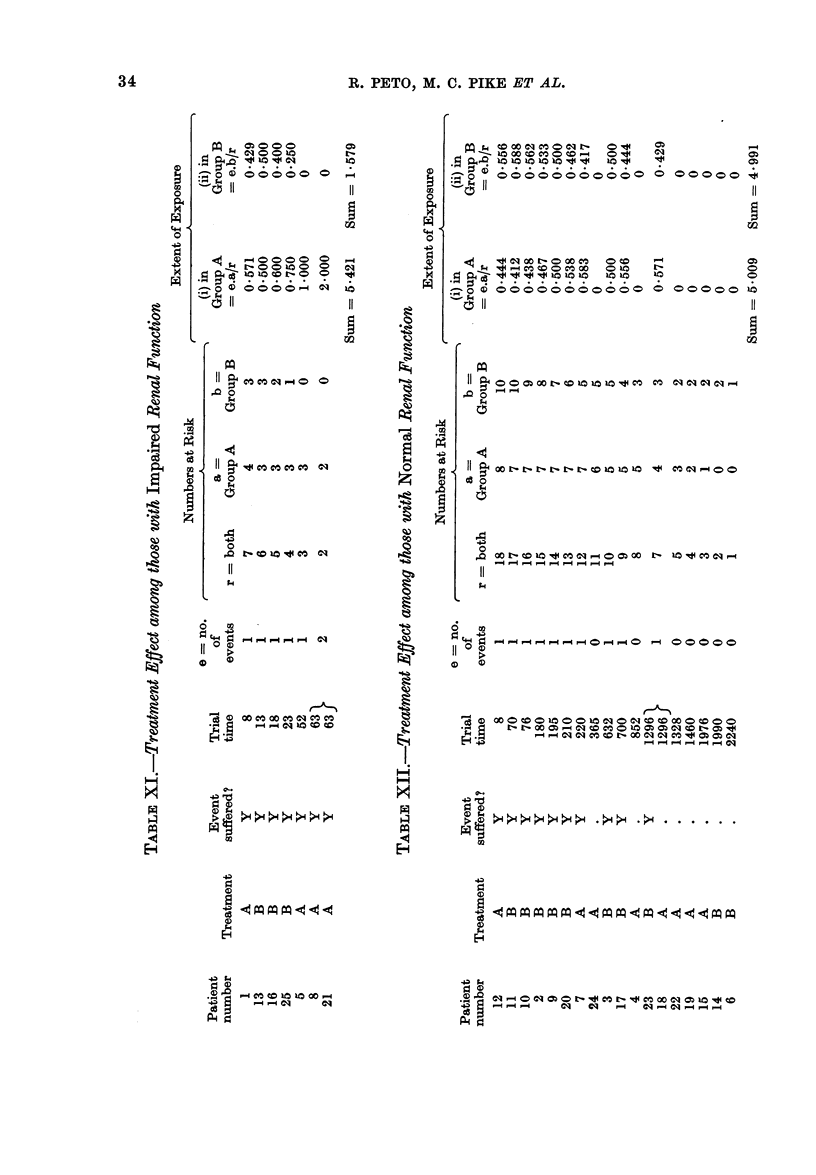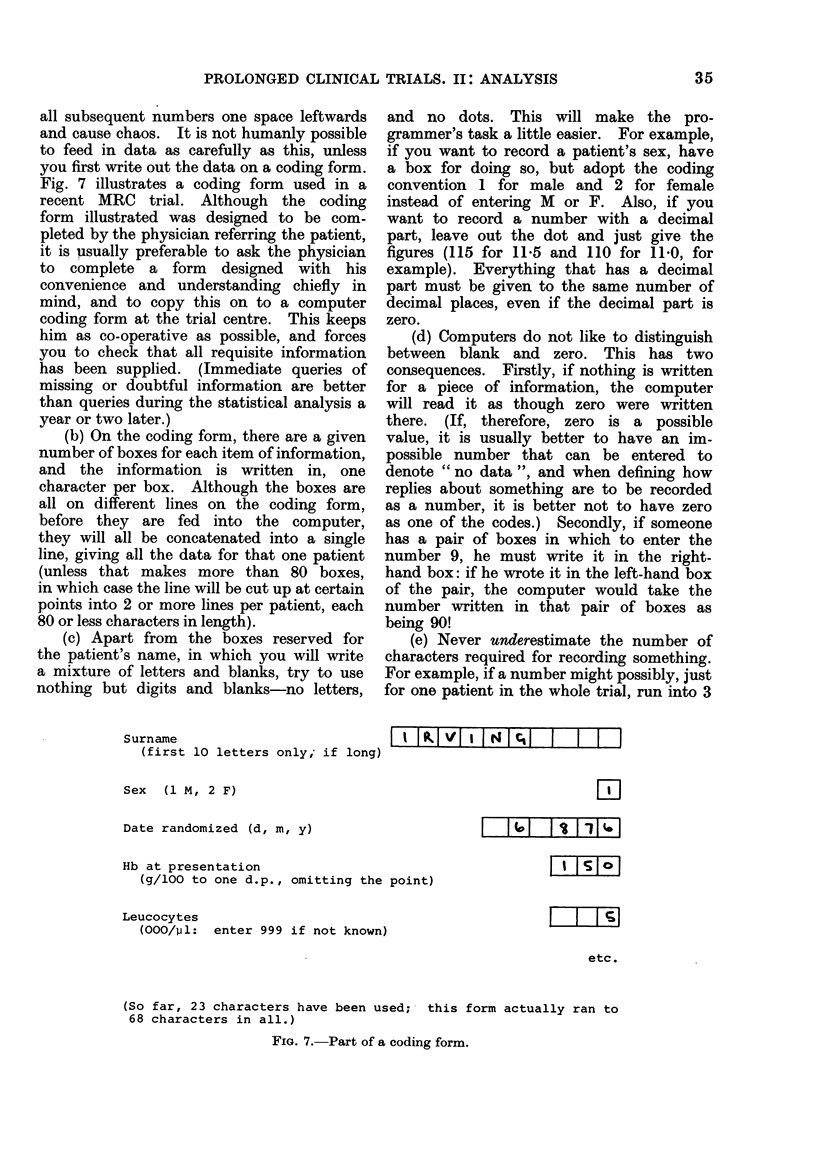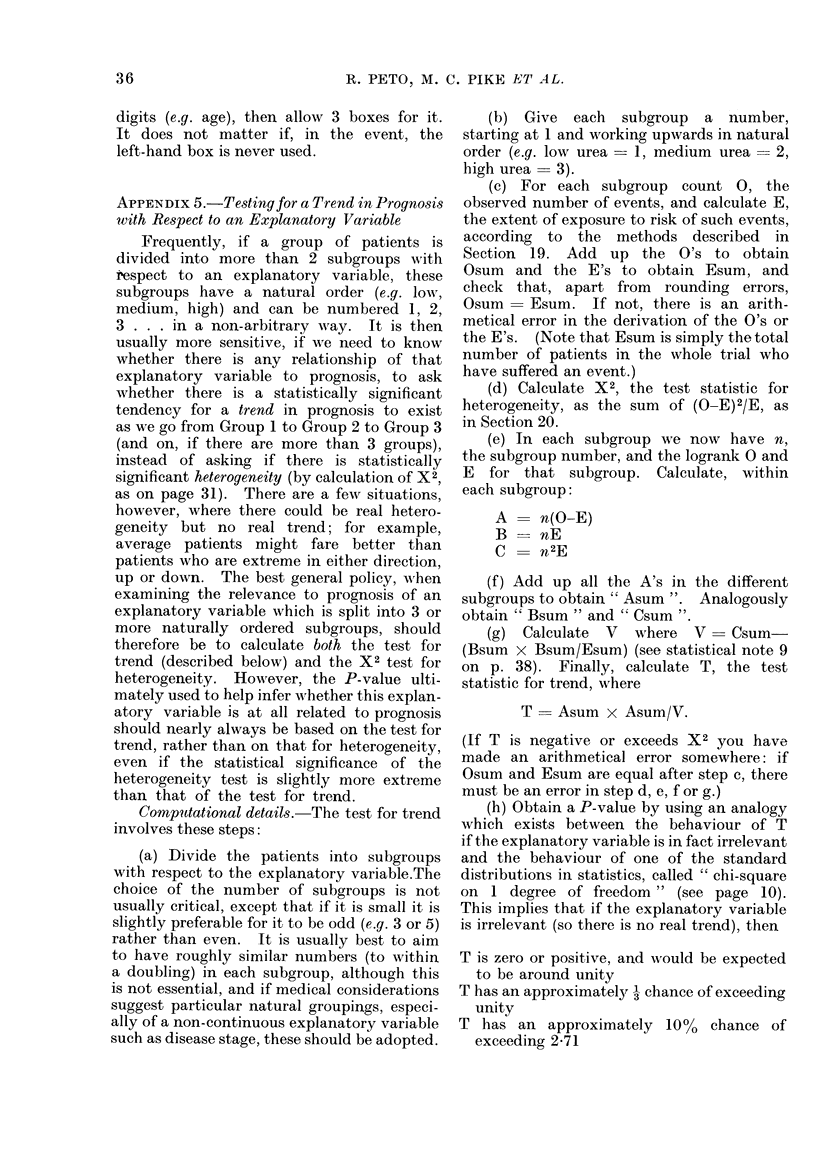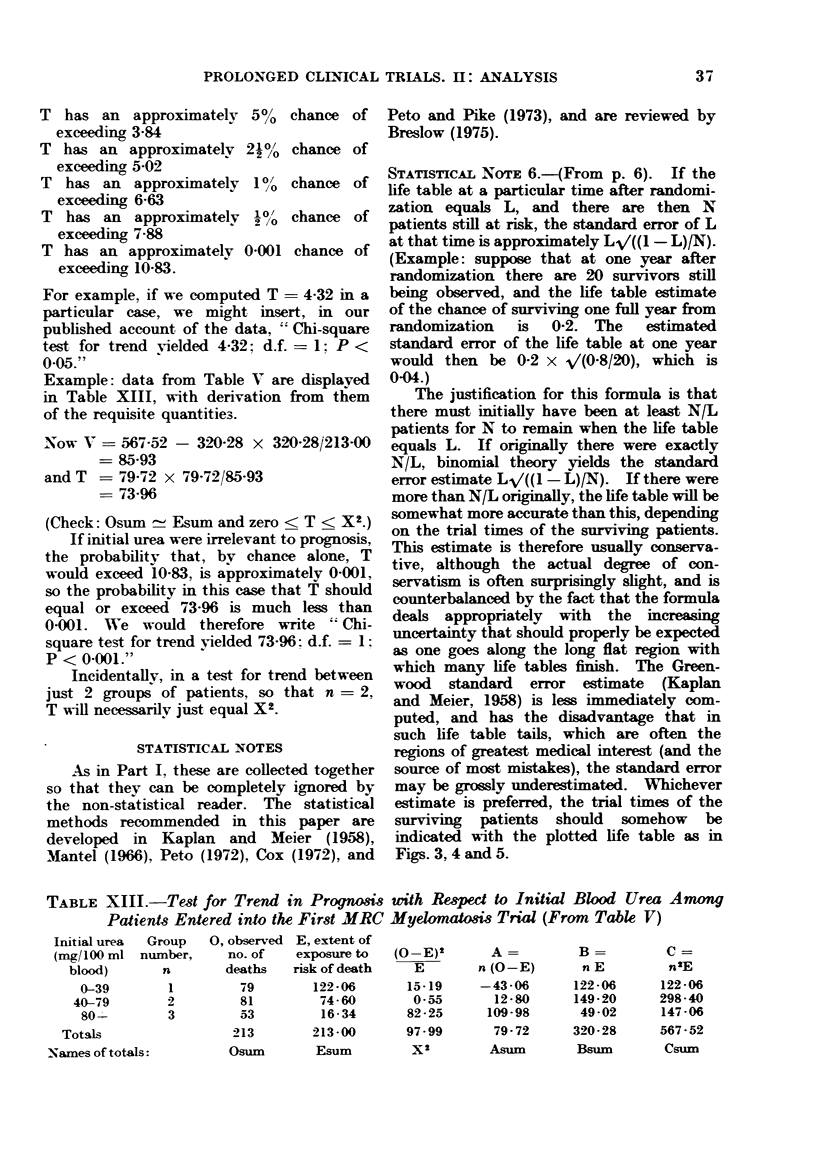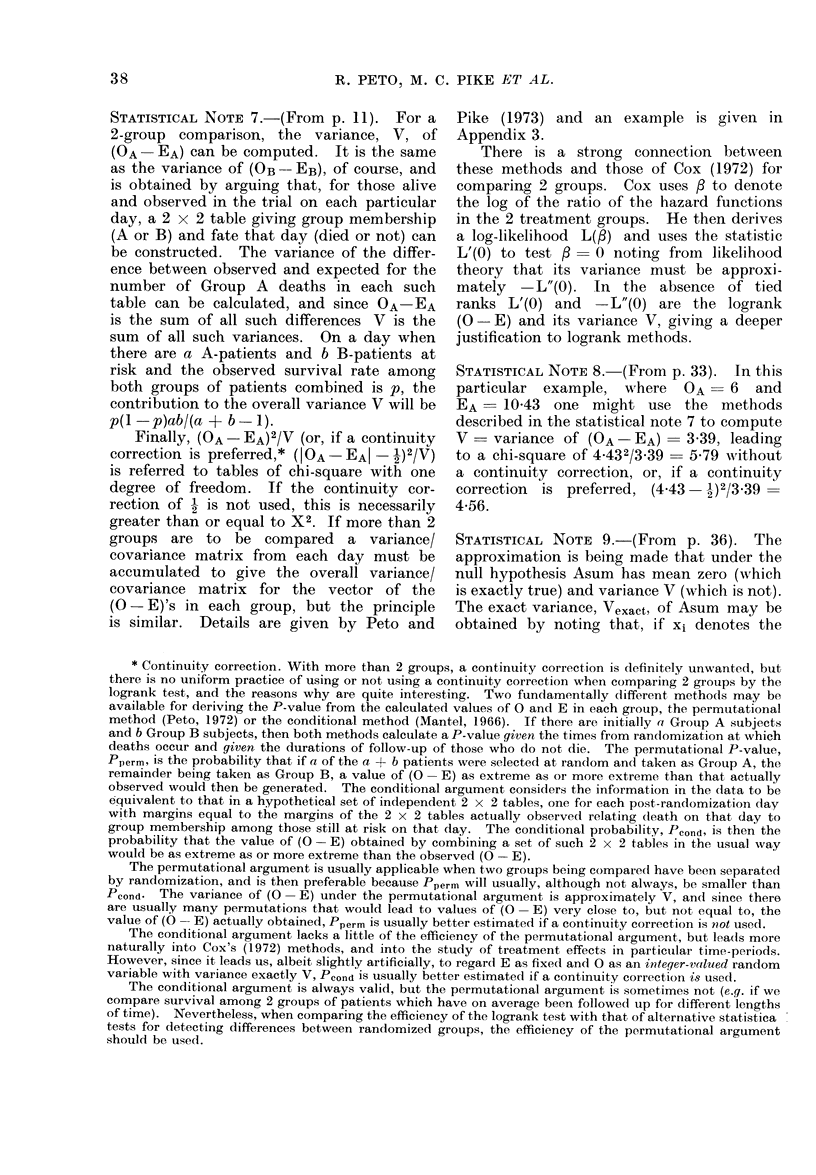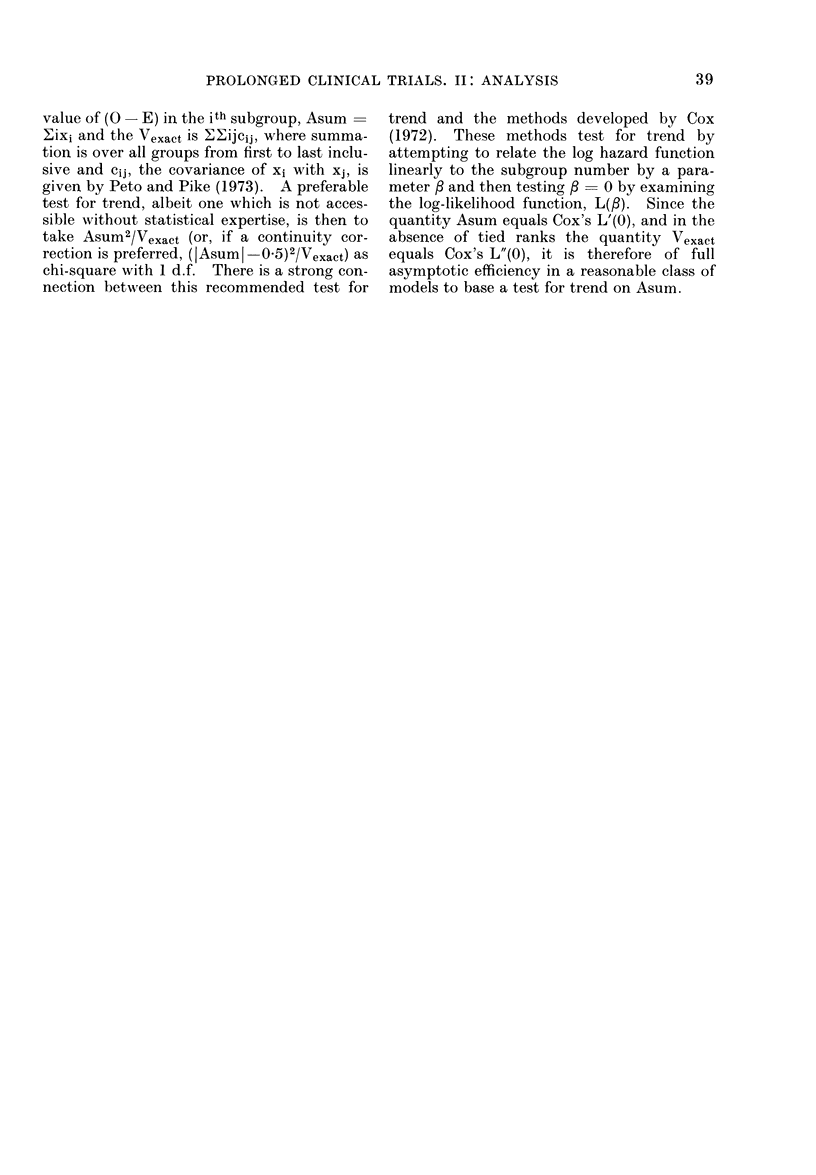# Design and analysis of randomized clinical trials requiring prolonged observation of each patient. II. analysis and examples.

**DOI:** 10.1038/bjc.1977.1

**Published:** 1977-01

**Authors:** R. Peto, M. C. Pike, P. Armitage, N. E. Breslow, D. R. Cox, S. V. Howard, N. Mantel, K. McPherson, J. Peto, P. G. Smith

## Abstract

Part I of this report appeared in the previous issue (Br. J. Cancer (1976) 34,585), and discussed the design of randomized clinical trials. Part II now describes efficient methods of analysis of randomized clinical trials in which we wish to compare the duration of survival (or the time until some other untoward event first occurs) among different groups of patients. It is intended to enable physicians without statistical training either to analyse such data themselves using life tables, the logrank test and retrospective stratification, or, when such analyses are presented, to appreciate them more critically, but the discussion may also be of interest to statisticians who have not yet specialized in clinical trial analyses.


*Br. J. Cancer* (1977) **35**, 1

### DESIGN AND ANALYSIS OF RANDOMIZED CLINICAL TRIALS REQUIRING PROLONGED OBSERVATION OF EACH PATIENT

### II. ANALYSIS AND EXAMPLES

R. PETO,[1] M. C. PIKE,[2] P. ARMITAGE,[1] N. E. BRESLOW,[3] D. R. COX,[4] S. V. HOWARD,[5] N. MANTEL,[6] K. McPHERSON,[1] J. PETO[1] AND P. G. SMITH[1]

*From* [1]*Oxford University,* [2]*University of Southern California,* [3]*University of Seattle,* [4]*Imperial College, London,* [5]*U.C.H. Medical School, London and* [6]*George Washington University*

*Report to the Medical Research Council's Leukaemia Steering Committee; Chairman, Professor Sir Richard Doll*

Received 22 December 1975 Accepted 25 August 1976

**Summary.—Part I of this report appeared in the previous issue (*Br. J. Cancer* (1976) 34, 585), and discussed the design of randomized clinical trials. Part II now describes efficient methods of analysis of randomized clinical trials in which we wish to compare the duration of survival (or the time until some other untoward event first occurs) among different groups of patients. It is intended to enable physicians without statistical training either to analyse such data themselves using life tables, the logrank test and retrospective stratification, or, when such analyses are presented, to appreciate them more critically, but the discussion may also be of interest to statisticians who have not yet specialized in clinical trial analyses.**

CONTENTS

ANALYSIS PAGE

16.—General principles . . . 2
17.—Definition of the "trial time" for each patient . . . 3
18.—The life table . . . 3
19.—The logrank test . . . 7
20.—Logrank significance levels (including chi-square tabulation) . . . 9
21.—Explanatory information (prognostic factors) . . . 11
22.—Use of prognostic factors to refine the treatment comparison . . . 12
23.—Bad methods of analysis . . . 14
24.—How much data should be collected from each patient? . . . 16
25.—Subdividing the follow-up period . . . 18
26.—Arranging the manner in which the data will be collected . . . 19
27.—Assessment by separate causes of death . . . 21
28.—Other end points . . . 21
29.—Remission duration . . . 23
30.—Combining information from different trials . . . 23

EXAMPLES

31.—Immunotherapy of acute leukaemia . . . 24
32.—The MRC myelomatosis trials . . . 27

Requests for reprints to R. Peto, Radcliffe Infirmary, Oxford, England; or to M. C. Pike, University of Southern California School of Medicine, Los Angeles, California 90033, U.S.A. Reprints of both parts will be sent to those who request reprints of either part. Bulk orders for teaching purposes cost £5 or $10 per 10; please inform us if any details are unclear, misleading or wrong.

REFERENCES FOR PART II . . . . . . . . . . . 28

APPENDICES FOR PART II
3.—Worked example of a clinical trial analysis (hypothetical data) . . 29
4.—How to record data in such a way that it is easy to analyse by computer . 33
5.—Testing for a trend in prognosis with respect to an explanatory variable 36

STATISTICAL NOTES FOR PART II . . . . . . . . . . 37

### ANALYSIS

### 16.—*General principles*

**Some of this introduction recapitulates text from Part I.**

Many clinical trials compare survival duration among cancer patients randomly allocated to different treatments. There has been much investigation in the statistical literature of possible ways of interpreting the data from such trials, the surprising outcome of which has been the discovery that 2 techniques (life table graphs and logrank *P*-values), which are so simple that they are easily mastered by non-statisticians, are commonly more accurate and more sensitive than any of the elaborate alternatives that have been considered. Part II of this report now describes these 2 techniques in sufficient detail for them to be performed entirely without statistical guidance. Inessential notes on statistical details are relegated to the end of the text, and should be ignored by most readers.

If the course of the disease is very rapid (*e.g.* acute liver failure) and it is unimportant whether a dying patient lives a few days longer or not, a count of the numbers of deaths and survivors on each treatment is all that is required. However, if (as with most forms of neoplastic disease) an appreciable proportion of the patients do eventually die of the disease, but death may take some considerable time, it is possible to achieve a more sensitive assessment of the value of each treatment by looking not only at how many patients died but also at how long after entry they died.

You can best learn statistical methods by applying them to data which interest you. If you have some data to interpret, then we hope that, when you have read this paper and applied it to your data, you will feel that the methods it describes are straightforward and that the use of them has simplified your data and helped you understand them. However, if you do not have any such data to interpret, perhaps you should not study the technical parts of this paper carefully, as attempting to learn the details of statistical methods in the hope of applying them at some vague date in the future usually produces confusion.

In Part II of this article, the first few sections describe how time to *death* may be analysed, ignoring entirely all assessment of the quality of life. The methods described, however, are equally useful for analysing time to some other first event; for example, in clinical trials of solid tumour therapy, a separate analysis of time from entry to first local recurrence may be of interest, or perhaps a separate analysis of time from entry to first metastatic spread. In later sections, we describe the ways in which the statistical methods used to study *death* rates may also be used to study rates of some other particular type of event in a clinical trial. Analysis of survival duration in a randomized trial would usually involve obtaining:

(i) *Descriptive graphs* of the observed outcome in each treatment group (" life tables ") which can be compared with each other visually.

(ii) A *P*-value to see if the observed differences between treatment groups could plausibly be just chance (using the " logrank test ").

(iii) Information about how survival differs between groups of patients who differ with respect to an " explanatory variable ", such as age or disease stage, recorded at the time of randomization (using life tables and logrank tests to compare these groups with each other).

(iv) Retrospective stratification, based on the findings in (iii), followed by recalculation of the *P*-value comparing treatment groups making proper allowance for which of these strata each patient is in.

17.—*Definition of the " trial time " for each patient*

**" Time " is measured for each patient from that particular patient's date of randomization.**

After deciding to analyse the results a stopping date, perhaps the end of a particular month, is chosen and for each patient in the trial it is determined whether he was alive or dead on that date and, if dead, the date on which he died. (Deaths occurring shortly after the chosen stopping date are ignored in this analysis, even if they are known of when analysis occurs, since otherwise the risk of death would be slightly exaggerated.) If the collaborating centres have enough advance warning of the stopping date, they can arrange appointments for all their surviving patients for a few days after this date, and the data collection can then be completed within a matter of weeks of the stopping date. It is certainly not sufficient to rely on busy collaborating physicians to notify a trial centre whenever deaths occur; delays of several months would then be commonplace, and some deaths might be completely missed. Curiously, recall (in response to telephone enquiries) of how long ago deaths occurred or patients were last seen is very unreliable, many events being remembered as being considerably more recent than they actually were. Exact dates when patients died or were last seen must, unfortunately, be determined.

For each patient one now knows whether or not he died and the time for which he was at risk, which we call his *trial time*. This runs from the date of his randomization to the date of his death or, if he did not die, to the stopping date. The trial time of a lost or emigrated patient runs to the date of loss; if loss may have occurred *because* therapy was not being successful (or because it has been completely successful), there is no satisfactory way of allowing for this fact, so don't let it happen! Note that survival in a therapeutic trial is measured from the date of randomization, not the date of first symptoms, presentation or starting treatment. Early deaths, occurring before treatment has even started, are thus included in the actual treatment comparison.

18.—*The Life Table*

**This is a graph or table giving an estimate of the proportion of a group of patients that will still be alive at different times after randomization, calculated with due allowance for incomplete follow-up.**

If all the patients in a trial have died, it is easy to calculate the proportion of patients surviving to the end of a particular day from randomization, and a graph of this proportion against time from randomization would be a simple *life table*, for the special case where all have died.

Unfortunately, this simple and sensible graph of " proportion alive " against " time since randomization " can be fully plotted only if all the patients are already dead before analysis of the data is undertaken. For example, if some of the patients are still alive with trial times less than one year (because they were randomized only a few months ago), we cannot yet know if they will eventually survive a full year from randomization or not, and so there is no simple and obvious estimate of the proportion of all patients who will be alive at one year.

However, to survive a whole year, a patient has to survive each of the 365 days comprising it, and this apparently trivial observation is the key to efficient estimation of how many will live the full year out. We need first to look at the death rates observed on each individual day, and then to argue that, for example, the way to live 31 days is to live 30 days and then to live one more day.

Translated into the language of probabilities, this means that the probability of living 31 days from randomization is the probability of living 30 days multiplied by the chance of surviving Day 31 after living 30 days: they are multiplied together since this is how one combines such probabilities. It is essential that the previous sentence be clearly understood, for without it the remainder of this section will be obscure. (It is just analogous to the calculation that if 2 coins are tossed in succession, the probability of both being heads is one-quarter, this being the product of the probability that the first coin is heads, which is one-half, and the probability, *after* the first coin has come down heads, that the second coin will now do so, which is also one-half.)

This simple rule is all that underlies the calculation of " life table " graphs. It follows fairly straightforwardly from it that the chance of living a year from randomization is

$$C_1 \times C_2 \times C_3 \times C_4 \times \ldots \times C_{364} \times C_{365}$$

where:

$C_1$ denotes the chance of surviving at least one day from randomization

$C_2$ denotes the chance of surviving a second day after you have survived one day from randomization

$C_3$ denotes the chance of surviving a third day after you have survived 2 days from randomization

$C_4$ denotes the chance of surviving a fourth day after you have survived 3 days from randomization

*etc.*, and:

$C_{365}$ denotes the chance of surviving Day 365 after you have survived 364 days from randomization.

Unfortunately, we do not know any of these individual $C$'s. However, we could estimate any particular one of them ($C_{365}$, for example) by looking to see what proportion of patients who are at risk on Day 365 actually survived it. Let us write this *observed survival rate* for Day 365 as $p_{365}$. We could use $p_{365}$, the *observed* survival rate on Day 365 among those alive after 364 days, as a very crude estimate of $C_{365}$, the actual *chance* of surviving Day 365 after you have survived 364 days. To calculate $p_{365}$, we study all patients with trial times greater than or equal to 365 days; in other words, all patients who entered the trial more than 364 days ago (so that we have a chance to see their fate on Day 365) and who were still alive 364 days after randomization. (Patients who entered only a few months ago tell us nothing about $p_{365}$.) $p_{365}$ is then simply the proportion of these patients who survive Day 365. If, as may well be the case, nobody happened to die exactly on Day 365, then $p_{365} = 1$. For every post-randomization day, $p$ can be defined analogously.*

The " life table " estimate of the true probability ($C_1 \times C_2 \times C_3 \times C_4 \times C_5 \times C_6 \times C_7$) of surviving 7 days from randomization is simply $p_1 \times p_2 \times p_3 \times p_4 \times p_5 \times p_6 \times p_7$; likewise, the " life table " estimate of the chance of surviving a whole year from randomization would be:

$$p_1 \times p_2 \times p_3 \times p_4 \times \ldots \times p_{364} \times p_{365},$$

a product of 365 observed survival rates.

* Formally, on Day 76 after randomization, $p_{76}$, the observed survival rate, equals (no. with trial time of at least 76 days who did not die on Day 76)/(no. with trial time of at least 76 days). The observed survival rate on Day 76 after randomization thus makes no use whatsoever of data from patients who died before Day 76, or who were randomized less than 76 days ago.

Although each individual observed survival rate ($p$) is a very inaccurate estimate of the corresponding chance of survival, ($C$) it is a surprising fact that the product of a lot of $p$'s (the life table estimate of the chance of still being alive after a certain time) is quite an accurate estimate of the product of the corresponding $C$'s (the actual chance of still being alive then).

It may be noticed that the life table estimate of the chance of surviving any particular number of days from randomization is thus the product of the life table estimate up to the previous day, and the observed survival rate for the particular day. The life table estimate is exactly the same as the simple proportion of survivors if all the patients die before the trial is analysed (readers can check this themselves for the first 6 days, for example). The " life table " (or " survival curve ") for a set of data is a graph or table of this estimate against time from randomization. (The usage has emerged that either a table or a graph may be referred to as a " life table ".)

A life table is the most accurate description of a set of data on the times to death of a group of patients, and physicians engaged in such clinical trials should be familiar with its construction and interpretation. In practice, one doesn't have to worry about the multitude of days on which nobody happened to die, for on these days $p = 1$ and a life table may stay constant for a whole run of such days.

Although the life table is much more reliable than the individual observed survival rates of which it is composed, spurious big jumps or long flat regions may sometimes occur in a plotted life-table; this is discussed more fully below.

In an MRC trial in chronic granulocytic leukaemia (MRC, 1968), previously untreated patients were admitted at several centres from September 1959 to December 1964. Analysis was undertaken with the stopping date of 1 January 1967. Fig. 3 is the life table for the entire group of 102 patients admitted to the trial. It is slightly irregular, as are all life tables based on small numbers of patients, but its general shape gives the best information that can be derived from the collected data about the pattern of mortality of these patients, although its fine detail is not really informative.

Fig. 4 gives the separate life tables for the 2 treatment groups of patients—those treated by radiotherapy and those treated with busulphan. Evidently, the busulphan-treated patients have fared somewhat better. This shows how treatment differences can be illustrated by survival curves.

If the vertical " % survivors " axis is given on a logarithmic scale, the slope of the survival curve at any given time

FIG. 3.—Life table for all patients in the Medical Research Council's first CGL trial. The numbers of patients still alive and under observation at entry and annually thereafter were: 102, 84, 65, 50, 18, 11, 3.

FIG. 4.—Life tables for the 2 separate treatment groups in the Medical Research Council's first CGL trial. The numbers of patients still alive and under observation at entry and annually thereafter were: busulphan 48, 40, 33, 30, 13, 9, 3; and radiotherapy 54, 44, 32, 20, 5, 2, 0.

FIG. 5.—Life table derived from the hypothetical data used as a worked example in Appendix 3. The numbers of patients still alive and under observation at entry and every 6 months thereafter were: 25, 14, 11, 10, 8, 7, 7, 7, 4.

estimates the death rate among the survivors at that time. This is sometimes useful as it shows how the death rate among the survivors depends on time from randomization, but since such data usually have to be presented to people who are not really familiar with logarithms, logarithmic axes should in general be avoided when presenting life tables, especially since they magnify the parts of the life table which are least accurate at the expense of the more accurate parts.

Fig. 5 gives the life table for the hypothetical data which are presented and analysed as a worked example in Appendix 3. Here, a common feature of many real life tables is apparent: the data are so sparse that there are long periods (*e.g.* between Day 250 and Day 600) when nobody happens to have died. The life table consequently consists of flat regions separated by " steps ", and again it must be emphasised that any conclusion based on the fine detail of such a graph is likely to be wrong. Particularly, long flat regions at the right-hand end of a life table do not imply that the real risk of death among patients who are still alive then is negligible, unless a large number of patients have trial times well into or beyond the flat region.

In Figs. 3, 4 and 5, the trial times of all individual patients, whether they died or not, are marked, so it is a fairly simple matter when looking at the graphs to see how many patients were still at risk at any one time. This is done by counting the points to the right of this time, and is especially valuable in life tables such as Fig. 5 where the data are sparse. If your trial is so big that it would be impossible to show a distinct point for each patient, write at several different times along the foot of the life table, the number of patients with trial times of at least those magnitudes. This has also been done in the legends to Figs. 3, 4 and 5, although it would really have been better if these numbers of patients remaining " at risk " had actually been written along the bottom of each graph.

The reason for wanting to know how many patients are alive and still being followed up at a particular time from randomization is that this information can be used for a quick (but quite accurate; see statistical note 6) estimate of how much your life table at that time might differ from the value it would have had in a vastly larger, and hence more accurate, study. However, these estimates should not usually be used for the calculation of $P$-values; for this, the logrank test, which is described below, is preferable since it takes into account the overall structure of the 2 curves being compared, not just their values at one time.

(Over periods as long as 5 or 10 years from entry, an appreciable proportion of a group of old patients would be expected to die from other causes, and so "adjusted" 5- or 10-year percentage survivals are sometimes cited. These are simply the life-table estimates of the proportion of the diseased patients still alive at 5 or 10 years as a percentage of the proportion that would have been alive had only the national age- and sex-specific death rates prevailed.)

19.—*The Logrank test*

**This involves counting the number of deaths observed in each group, O, and comparing it with E, the extent of exposure to risk of death in that group; the method of calculating E is given below.**

The basis of the logrank test is so straightforward that it seems surprising that it was first suggested only as recently as 1966 (Mantel, 1966). Breslow (1975) has recently written a unified statistical review paper on the logrank test and allied approaches to clinical trial data, which can be consulted for formal justification of its widespread use.

The principle is that if, for example, of the patients under observation on a particular day after randomization, two-thirds are in Treatment Group A and one-third are in Treatment Group B, then on average two-thirds of the deaths on that day should occur among A patients and only one-third among B patients, unless A is really a more, or a less, effective treatment than B. We may define the *extent of exposure to risk of death* of A patients on that day to be two-thirds the number of deaths on that day, and that of B patients to be one-third of the number of deaths on that day.*

When considering a particular day after randomization, a little care is needed to work out the proportions of A patients and B patients, since patients who have died before this day must be ignored, as must patients randomized so recently that their fate on this particular day is not yet known. (In this respect, the calculation resembles the calculation of the life table.) For example, if in a clinical trial, 100 patients are randomized to A and 100 to B, then on Day 1, 200 patients are at risk, half in Group A and half in Group B. If, however, due to death, loss or recent entry, only 60 of the A patients plus 40 of the B patients have trial times of 365 days or more, then on Day 365, 100 patients are at risk, with proportions 0·600 in Group A and 0·400 in Group B. If 2 deaths occurred among these 100 patients on the 365th day after randomization, the extent of exposure to risk of death suffered by the A patients on Day 365 would be 1·200 and that suffered by the B patients would be 0·800. In other words, risks are related to the proportions remaining, not to the proportions originally randomized. Note, of course, that no calculations need be performed for days on which no deaths occur in either group, since the quantity which we have chosen to call the extent of exposure to risk of death will necessarily be zero on all such days. (This illustrates that our definition of the extent of exposure to risk on a particular day depends on what actually happened on that day, not on what might have happened.)

The actual number of deaths observed on a certain day among the Treatment A patients will usually not exactly equal the extent of exposure to risk on that day, especially as this is likely not to be an exact whole number. The number of deaths actually observed among the A patients may be less than the extent of exposure of the A patients to risk on some days and more on other days. If A and B are equivalent treatments, however, then over a long period (comprising many

* The general definition is that the extent of exposure to risk of death among a subgroup of patients on a particular day is the total number of deaths on that day in the whole study population, multiplied by the proportion of the patients at risk on the particular day who are in the subgroup of interest: see Appendix 3 for a worked example.

individual days) the total number of deaths observed in A patients should on average equal the sum of all the separate extents of exposure of the A patients to risk of death on each separate day during this period.

The logrank* test comparing Treatment A with Treatment B during a certain period involves:

(i) counting the total number of Group A *deaths observed* during that period, calling this $O_A$;
(ii) counting the total number of Group B *deaths observed* during that period, calling this $O_B$;
(iii) calculating the extents of exposure of the A patients to risk during each day of the period, adding them all up to get the total *extent of exposure to risk of death* suffered by the A patients during this period, calling this $E_A$;
(iv) deriving similarly the total *extent of exposure to risk of death* suffered by the B patients during this period, calling this $E_B$;
(v) comparing $O_A$ with $E_A$ and $O_B$ with $E_B$, to see if there are any marked discrepancies. Table IV gives such a comparison for the data from the whole of the MRC chronic granulocytic leukaemia trial (MRC, 1968).

As an arithmetic check, $O_A + O_B$ should equal $E_A + E_B$, except for slight rounding errors. (When calculating the extents of exposure to risk on individual days, it suffices to work to 3 decimal places.) It follows that if $O_A$ exceeds $E_A$, indicating that Group A fared worse than the average of Groups A and B together, then $O_B$ will be less than $E_B$, indicating that Group B fared better than average.

This method generalises instantly to the comparison of several groups of patients with each other: for each group, the extent of exposure to risk of death on a particular day is still the proportion on that day who are in that group times the number of deaths on that day; and again, the total exposure in one group over an extended period is the sum of the separate exposures in that group on the separate days comprising the period. In any one period, too, the sum of all the O's will equal the sum of all the E's. For example, if we were comparing 4 groups, A, B, C and D, we would finally check that: $O_A + O_B + O_C + O_D$ equals $E_A + E_B + E_C + E_D$.

Two questions are unanswered: " What periods should be examined? " and " What constitutes a *marked* discrepancy between O and E? " The second question is dealt with in Section 20. The answer to the first question depends on the disease being studied; for acute myeloid leukaemia, where there are many early deaths during the first few months after randomization, followed by partial stabilization, it might be sensible to look separately at what happens in 2 periods, the first including all days in the first 6 months after randomization, and the second comprising all subsequent days. Alternatively, for another disease, one might look separately at the apparent treatment differences in 3 periods, the first year, the second year,

TABLE IV.—*MRC Chronic Granulocytic Leukaemia Trial*

| Treatment group | No. of patients in group | O, observed no. of deaths | E, extent of exposure to risk of death | Relative death rate, O/E |
|---|---|---|---|---|
| Busulphan | 48 | 40 | 51·95 | 0·77 |
| Radiotherapy | 54 | 50 | 38·05 | 1·31 |
| All patients | 102 | 90 | 90·00 | 1·00 |

* The name " logrank " derives from obscure mathematical considerations (Peto and Pike, 1973) which are not worth understanding; it's just a name. The test is also sometimes called, usually by American workers who cite Mantel (1966) as the reference for it, the " Mantel–Haenszel test for survivorship data ".

and all subsequent years. Whatever periods the time from randomization is split into, the most important comparison is that for all periods together. This is the "logrank test" comparing the overall difference between the whole survival curve for A and that for B.

Tables (such as Table IV) of observed numbers of deaths, O, and extents of exposure to risk of death, E, for the whole period of observation, give a concise summary of the trial results.* The ratio O/E for a subgroup is called the *relative death rate* for that subgroup, because it approximates to the ratio of the daily death rate in that subgroup to the daily death rate among all groups combined. Therefore, the ratio of 2 O/E's from different subgroups can be used to describe the apparent ratio of the corresponding death rates. For example, in Table IV, the relative death rates on the 2 treatments are 0·77 and 1·31, suggesting that the true death rate ratio is about 0·77/1·31 = 0·6: *i.e.*, very crudely, that busulphan prevents or delays about 40% of the deaths that would occur with radiotherapy.

Actually, in one group the death rate will probably not be constant; it might, for example, be more rapid among patients who have only just been randomized than among patients who have been in the trial for over a year. If the death rate in one group is thus not constant, how can we talk meaningfully about the ratio of the death rates in 2 groups? If time from randomization is subdivided into periods which are short enough for the death rates not to vary much within one time period, then within each separate period it is meaningful to talk about the death rates in 2 subgroups of patients, and the ratio of these 2 death rates. Some sort of average of these death rate ratios in different time periods could be formed, and this "average death rate ratio" is what is really estimated by the ratio of 2 O/E's for 2 subgroups of patients.

A statistical test based on the differences between O's and E's is optimal, in the sense that if there really is a slight difference in the efficacy of the treatments (whereby the death rate in one group consistently exceeds that in the other group by a certain proportion: see statistical note 5 in Part I) no other valid statistical method is as likely to yield a significant difference. We shall now describe how *P*-values are calculated from the differences between O's and E's to help decide whether such differences could plausibly have occurred by chance alone.

20.—*Logrank significance levels*

**$P$-values may be estimated by comparing the sum of $(O-E)^2/E$ with an appropriate chi-square distribution.**

The approximate statistical significance of differences between observed numbers of deaths, O, and extents of exposure to risk of death, E, in different groups, can be calculated quite rapidly.

In each group we can calculate $(O-E)^2/E$. The more discrepant the value of O in a particular group is from the value of E in that group, the bigger $(O-E)^2/E$ in that group will tend to be. Suppose that we calculate $(O-E)^2/E$ in each group and that we then add these up, one term from each group. This is something which we shall want to discuss in many places in this paper. It is therefore convenient to have a brief name for the sum of all the $(O-E)^2/E$ values, and we choose to call it $X^2$. Likewise, we shall let the symbol $k$ denote the

* There is obviously some sort of analogy between E, the total extent of exposure in a subgroup, and an expected number of deaths in that subgroup, and because of this, E is often referred to as the "expected number of deaths". Unfortunately, in a group of patients who stay alive longer than average, E may, as in Table IV, exceed the number of patients originally randomized into a group. Since it would seem paradoxical to "expect" more deaths than there are patients, the name "extent of exposure" for E is perhaps preferable. However, both names are now sanctioned by usage in published work and, whichever name is used, the statistical arguments are equally valid.

number of groups being compared with each other: in many clinical trial analyses, $k = 2$.

If there are $k$ treatment groups and the prognosis of each group is, in fact, the same, then $X^2$ will usually be roughly equal to ($k$–1).* If, on the other hand, the prognosis in different groups is really different, the observed numbers, O, in each group will be systematically different from the corresponding extents of exposure, E, and $X^2$ will tend to be greater than $k$–1. Large values for $X^2$, therefore, although they *could* arise by chance, constitute evidence for real differences between the prognoses in the $k$ groups. It is possible to calculate the approximate probability that $X^2$ would, if the prognosis were the same in all $k$ groups, exceed any particular given value—and, of course, the larger the given value the less probable this is.

This probability, which we call the "significance level" or "$P$-value", is estimated by an analogy between the behaviour of $X^2$ if the $k$ treatments were identical and the behaviour of one of the standard distributions of statistics, the chi-square distribution. Actually, there are lots of different chi-square distributions, each with a different mean value; we can have a chi-square distribution with mean 1, a chi-square distribution with mean 2, and so on: the mean value of a particular chi-square distribution is called the "degrees of freedom" of that chi-square distribution, for reasons which are not essential here. Since, if the prognoses are the same in all $k$ groups, the expected value of $X^2$ is approximately ($k$–1), we shall use the analogy with the chi-square distribution with mean ($k$–1). This comparison enables us to say that $P$, the significance level, is approximately the probability that an ordinary chi-square distribution with $k$–1 degrees of freedom shall equal or exceed the observed value of $X^2$. Some of these probabilities are listed in the footnote.†

As an example of the use of these methods, consider the data of Table IV. There are 2 groups, so $k = 2$ and $X^2$, the sum of $(O-E)^2/E$, is

$$(-11{\cdot}95)^2/51{\cdot}95 + (11{\cdot}95)^2/38{\cdot}05 = 6{\cdot}50.$$

(*N.B.* This sum has only one term for each group, based on the numbers of deaths: no contributions come from the numbers of survivors.) Comparison of this value with the tabulated behaviour of chi-square with one degree of freedom shows that the observed difference between the 2 treatments is more extreme than would commonly arise by chance alone: since $5{\cdot}02 < 6{\cdot}50 < 6{\cdot}63$, $0{\cdot}025 > P > 0{\cdot}01$ and since 6·50 nearly equals 6·63, $P \simeq 0{\cdot}01$. We might, in a publication, say "The difference is statistically significant ($X^2 = 6{\cdot}50$, d.f. = 1, $P \simeq 0{\cdot}01$)."

More precise significance levels can be

* Although the reasons for this rough equality will not be apparent to most non-statisticians, it must unfortunately be taken on trust, as its proof is beyond the scope of the present paper; the same is true of the chi-square analogy which follows.

† For any mean value (1, 2, 3, 4 . . .), the chi-square distribution with that mean has a probability just under 0·05 of exceeding (mean + 3$\sqrt{}$ mean). For comparisons of 2, 3, 4, 5 or 6 groups with each other, the minimal value of $X^2$ necessary to generate certain particular $P$-values is tabulated.

| No. of groups of patients being compared | Mean value of $X^2$ (*i.e.* degrees of freedom of chi-square analogue) | Minimal $X^2$ | | | | | |
|---|---|---|---|---|---|---|---|
| | | $P < 0{\cdot}1$ | $P < 0{\cdot}05$ | $P < 0{\cdot}025$ | $P < 0{\cdot}01$ | $P < 0{\cdot}005$ | $P < 0{\cdot}001$ |
| 2 | 1 | 2·71 | 3·84 | 5·02 | 6·63 | 7·88 | 10·83 |
| 3 | 2 | 4·61 | 5·99 | 7·38 | 9·21 | 10·60 | 13·81 |
| 4 | 3 | 6·25 | 7·81 | 9·35 | 11·34 | 12·84 | 16·27 |
| 5 | 4 | 7·78 | 9·49 | 11·14 | 13·28 | 14·86 | 18·47 |
| 6 | 5 | 9·24 | 11·07 | 12·83 | 15·09 | 16·75 | 20·52 |

calculated for publication purposes (Peto and Pike, 1973—see statistical note 7 on p. 38) if statistical assistance is available, and these will usually be slightly more extreme than the significance levels derived by this simple chi-squared analogy. A worked example of the use of all the methods so far described on some hypothetical clinical trial data is given in Appendix 3, where life tables, O's and E's and *P*-values are calculated from first principles.

21.—*Explanatory information (prognostic factors)*

**If patients are retrospectively divided into strata, life tables and logrank methods can compare the prognosis in different strata, testing for heterogeneity or, if possible, for trend.**

*Explanatory information* is any data that can help to explain some of the differences between the survival times of different individuals. Broadly speaking, any facts collected from *all* the patients *before* their entry to a clinical trial can be used in this way without difficulty. Incomplete information is of much less use, and it may not be possible to deal with it in an unbiased way. Information collected once treatment is under way may be valuable, if it is collected from all the survivors at a particular time after their first treatment. This is, however, usually more difficult to arrange than is anticipated when the trial is designed, so data collected at the time of original randomization is usually of the greatest value.

With several items of explanatory information available from each patient, it is possible to determine (using life tables and the logrank test, but testing between groups defined by the explanatory variables instead of between different treatment groups) which (if any) of these items are correlated with prognosis. It is also possible to test whether an apparent influence on prognosis is merely due to an association with another, possibly more important, factor (Cox, 1972; Breslow, 1975). There is a worked example of this in Appendix 3.

For example, in analysing the MRC myelomatosis trial (MRC, 1971*a*), there was no apparent difference between the 2 treatment schedules tested, and interest turned to these explanatory variables. Table V gives the observed numbers of deaths, and the extents of exposure to risk of death in this trial, according to the blood urea of the patients at presentation. It can be seen from the column of values of O/E that the lower the initial blood urea, the better the prognosis.

We could, of course, check how easily heterogeneity as extreme as, or more extreme than, that actually seen between the O's and E's in Table V could arise simply by chance, by calculating:

$$X^2 = \frac{(79 - 122{\cdot}06)^2}{122{\cdot}06} + \frac{(81 - 74{\cdot}60)^2}{74{\cdot}60} + \frac{(53 - 16{\cdot}34)^2}{16{\cdot}34}.$$

In this case $X^2 = 97{\cdot}99$ and, since there are 3 groups, it is appropriate to compare $X^2$ with tables of chi-square on 2 degrees of freedom. The previous footnote shows that the probability of chi-square with 2 degrees of freedom exceeding 13·81 is 0·001. The probability is therefore much, much less than 0·001 that $X^2$ should attain a value as large as or larger than 97·99 by chance alone. In publishing such data, we might therefore write "$X^2 = 97{\cdot}99$, d.f. = 2, $P < 0{\cdot}001$" (or even, for emphasis, $P \ll 0{\cdot}001$).

This value of $X^2$ is so extreme that it answers the question of chance beyond doubt. However, in less extreme cases, it is preferable to make use of the fact that the groups are ordered, and to test for the existence of a *trend* in prognosis as we go from the first group to the last group. The trend test seeks not just heterogeneity, but *plausible* heterogeneity, in which the middle group (or groups) tends to have a more average prognosis than the outer groups, and the outer

TABLE V.—*First MRC Myelomatosis Trial*

| Initial urea (mg/100 ml blood) | No. of patients in group | O, observed no. of deaths | E, extent of exposure to risk of death | Relative death rate, O/E |
|---|---|---|---|---|
| 0–39 | 113 | 79 | 122·06 | 0·65 |
| 40–79 | 92 | 81 | 74·60 | 1·09 |
| 80+ | 53 | 53 | 16·34 | 3·24 |
| All patients | 258 | 213 | 213·00 | 1·00 |

groups tend to differ in opposite directions from the average prognosis. Whenever more than 2 groups are being compared, and they do have a natural ordering, it is likely to be more sensitive to test for trend than to test for heterogeneity. Technical details of how to test for trend are relegated to Appendix 5.

However, when a plausible contrast is as marked as that in Table V (the relative death rate in the high-urea group being about 5 times as big as that in the low-urea group), no sane reader will suppose that the differences between the O's and E's arose simply by chance. This particular $P$-value, therefore, answers an irrelevant question, and need hardly be cited; the most important thing with such data is to characterize the difference, not to test whether it could be due to chance or not. To describe the dependence of prognosis on initial blood urea, we might calculate separately:

(i) the life table for the low-urea patients,
(ii) that for the medium-urea patients,
(iii) that for the high-urea patients, and plot all three of them on a single graph of " estimated % alive " versus " time since entry to study ".

22.—*Use of prognostic factors to refine the treatment comparison*

**If a treatment difference among patients in one stratum is calculated, the sum of all such differences, one per stratum, yields an overall test of whether treatment matters among otherwise similar patients.**

In clinical trial analysis, we are interested in whether apparent differences between treatments might be due merely to random allocation of more of the good-prognosis patients to one treatment than to the other treatment. Obviously, anything we know about the major determinants of prognosis can help us to answer this question correctly, and help us to see whether, given the different numbers on each treatment in various prognostic categories, there is any residual relationship of treatment with survival.

In earlier reports of clinical trials, the first step in the analysis was often to examine the percentage of each favourable and unfavourable prognostic feature in each treatment group and, hopefully, to demonstrate that they were not too different. To ensure this, a policy of initial stratification was sometimes adopted, giving alternate patients in each particular prognostic stratum alternate treatments. With modern methods of analysis of survival data, it does not matter if there is some imbalance of prognostic features between treatments, and stratification on entry is usually unnecessary. The principle underlying these methods is very simple: when the trial is being analysed, find out which of the factors recorded at entry are relevant to prognosis (by the method of the previous section). In the light of this analysis, define a few " prognostic strata ", so that within each stratum the patients all, as far as could have been told at entry to the trial, had a fairly similar prognosis.

This is straightforward, if *only one* of your explanatory variables is strongly related to prognosis. If there is a natural way of subdividing that one important variable (*e.g.* male/female, or Stage I/ Stage II/Stage III/Stage IV), then use these natural subdivisions to define your strata. If it is a continuous variable, for example haemoglobin, you might first try subdividing it fairly finely (*e.g.* into 6 to 10 subgroups) and calculate the observed numbers of deaths, O, and the extents of exposure to risk of death, E, in each such subgroup. Finally, calculate O/E in each subgroup and pool adjacent subgroups with roughly similar values of O/E, to give yourself a few larger strata.

If you have only *two* important explanatory variables to allow for, then first use this approach to each one separately, splitting each into as few categories as possible. If you can manage to split the patients into only 2 or 3 categories with respect to each of the 2 important variables, then your strata might well be the 4, 6 or 9 different combinations of categories of these 2 variables. Stratification with respect to as many as 3 variables is often not necessary, and stratification with respect to more than 3 variables is usually both unnecessary and unwise, unless you have thousands of patients in your study.

Let us suppose that you have now defined, on the basis of explanatory information recorded at entry into the trial, a few retrospective strata. Within the first of these prognostic strata calculate, as above, the observed numbers of deaths and the extents of exposure to risk of death on each treatment, entirely ignoring all the patients in all the other strata. Within the first stratum, the sum of the observed numbers on the various treatments will necessarily equal the sum of the various extents of exposure. If all the treatments being compared are equivalent, then for any one treatment group in this first stratum, the observed number and extent of exposure will differ from each other only by random fluctuation. If one treatment is better than the other(s), however, then for that treatment in this stratum, the observed number of deaths is likely to be less than the extent of exposure, although since we have only looked at a fraction of all the patients in the trial so far, this difference is unlikely to be significant. However, we next repeat this analysis for the patients in the second prognostic stratum, and then for the third prognostic stratum, and so on. For a particular treatment, we now have an observed number and an extent of exposure in every stratum, which differ from each other only randomly, unless treatment matters. These may be added, to obtain a grand observed number, O, and a grand extent of exposure, E, for that treatment. Even if there is no very significant treatment effect within any single stratum, differences in the same direction in several strata can reinforce each other so that the *grand* O's and E's in certain treatment groups eventually differ from each other significantly, if some treatments really are better than others.

Comparison of these grand observed numbers and extents of exposure (by calculating $X^2$, as previously, and comparing it with the standard chi-square distribution with mean one less than the number of treatments) is not biased in any way by chance correlations between particular prognostic strata and treatment, and statistical tests for significant differences between the grand O's and E's are therefore the best way to assess real treatment benefits. In the MRC myeloma trial, inspection of Table V led us to define 3 prognostic strata (low, medium, and high urea) and after this stratification we eventually found that there was no significant effect of treatment among patients with any given level of urea. Similar techniques can also, of course, be used to examine the relevance of one factor to prognosis, with other factors being constant. A computer programme capable of doing all such analyses and of plotting or printing life-tables is available on request (see p. 20), and a worked example of the use of explanatory information is given in Appendix 3. An instructive and interesting example of the use of these methods on real data is provided by the report of the Medical

Research Council's fourth and fifth therapeutic trials in acute myeloid leukaemia (MRC, 1974).

In multi-centre trials, the differences between the prognoses of patients entered at different centres can be substantial. To allow for this is simple: stratify with respect to *centre*, and within each centre calculate observed numbers of deaths and extents of exposure to risk of death as described above with respect to treatment (or some explanatory variable). Finally, add up all the observed numbers, and all the extents of exposure for one treatment (or explanatory variable category), obtaining a grand O and E for that treatment (or category). $X^2$, calculated from the grand O's and E's for all treatment groups, provides a valid test of whether any real differences between treatments exist. This is unbiased whether or not there is marked heterogeneity in the types of patients admitted or in the general standards of medical management at different centres.

One advantage of dividing the patients into retrospective strata is that if one treatment is better than the other, it is sometimes much more so among certain types of patients than among others. A trial where this might be the case is described in the Example of Section 31, where methotrexate appears to be of substantial benefit in acute lymphoblastic leukaemia remissions only if the white blood count is low. However, it is extremely important not to be misled into seeing effects like this (which are called " interactions ") in every set of data analysed. If patients are divided into 3 or more strata, then since each stratum is smaller than the whole study, purely random differences between treatments will be more marked in each stratum. These differences may well point in opposite directions in different strata, giving the impression of an interaction, whether one is really there or not. The fundamental *P*-value to be reported is the *overall* comparison of treatments, adjusted by retrospective stratification. If this is not significant, it is unwise to conclude without expert statistical assistance that any treatment differences in individual strata are real.

A fuller discussion of statistical methods for the identification and use of prognostic factors may be found in Armitage and Gehan (1974).

23.—*Bad methods of analysis*

**A list is given of some common methods of analysis of survival data which are either inefficient, misleading or actually wrong.**

(1) The comparison of life tables at one point in time, ignoring their structure elsewhere, is in general inefficient (except for diseases which are very rapidly fatal or cured). Moreover, if the point at which the comparison is being made is chosen (*e.g.* Mathé *et al.*, 1969) *because* the difference there is substantial, the comparison is invalid unless special statistical methods are used, and these are inefficient.

(2) If few patients are at risk for more than a certain time, and after that time none of these few happens to die, there will be an apparent " plateau " in the life table. Such plateaux at the ends of life tables are very common, and should never be taken as evidence that " after a certain time most patients are cured " unless there are *large* numbers of patients still at risk at the time of the plateau. (Likewise, a sudden and meaningless big drop can sometimes occur near the right-hand end of a life table.)

(3) " Median* survival times " are

* Definition: If half the patients will die within a certain time from their randomization and half will live longer than that time, that time is the " Median survival time " for these patients. It can be estimated by calculating the life table for these patients and seeing on which day the life table (which estimates probability of surviving) crosses 50%. If the life table has long flat regions near 50% then this estimated median will be very imprecise. For example, in Fig. 5 the median suggested by the graph is at 210 days—but if 2 of the early deaths had been avoided the median would have been at 630 days!

very unreliable unless the death rate around the time of the median survival is still high. Even in quite extensive data, median survival times can be very inaccurate. Although median survival times are widely cited, they should therefore be treated with great caution, except for diseases in which nearly everyone dies, the data are extensive, and the life table falls rapidly through the whole region between 70% and 30% alive (the region in which the life table is used to estimate the median). Average survival times can be far worse, and should almost *never* be cited.

(4) A simple count of the numbers dead in each group is inefficient (except for diseases which are rapidly fatal or cured), as it wastes the information as to exactly when each death occurred.

(5) The best estimate of the probability of living 4 years, say, from randomization, is given by the value of the life table at 4 years. This is because the life table makes proper use of partial data, from patients who have been studied for only part of the first 4 years of their disease. Estimates other than the life table should never be constructed without expert statistical guidance. A less accurate but valid estimate is given by the proportion of the people who were randomized 4 years or more before the stopping date, who were still alive 4 years after their randomization. The number of deaths by a certain time divided by the total number originally randomized (*e.g.* Mathé *et al.*, 1969) systematically underestimates the risk of death if some patients only entered recently, since the recent patients have not yet had their full chance of dying. Conversely, the number of dead before year 4 divided by the number dead before year 4 *plus* the number surviving at year 4 (a common error) systematically overestimates the risk of death by year 4, since recent patients could not count among the living but inflate the number dead.

(6) Study of survival from first treatment rather than from randomization is undesirable, especially if the treatments being compared are such that the delays in initiating them in ill patients might differ. Study of survival among those who have lived long enough for a certain number of courses of treatment to have been given may misleadingly exaggerate the chances of survival. These are not absolute statistical prohibitions, of course, just warnings!

(7) The connection of the bottoms of the steps of life tables, such as that in Fig. 5 by sloping lines is improper, as it results in a graph which is a biased estimator of the proportion surviving at a given time. (Connection of the tops of the steps with each other would be oppositely biased.)

(8) Other significance tests could be used instead of the logrank test—for example, Gehan's (1965) modification of the Wilcoxon rank sum test is used in many American studies, and it is certainly a valid method to use. The advantage of the logrank test is that if there really is a slight difference between the groups being compared, whereby the death rate in one group consistently exceeds that in the other group by a given proportion, then this difference is more likely to be detected by the logrank than by any other valid assumption-free test (see statistical note 5 in Part I of this report).

(9) Believing that a treatment effect exists in one stratum of patients, even though no overall significant treatment effect exists, is a common error. Belief that a treatment difference exists should chiefly be based on the overall sum of all the within-stratum treatment comparisons. If this is clearly significant, serious consideration may then be directed to discovering whether the difference between the two treatments is more marked in some strata than in others. (This would be described by a statistician as an "interaction" between treatment and certain strata; the statistical use of this word resembles the medical use of the word "synergism".) However, marked heterogeneity of the treatment comparison in different strata can arise by chance more easily than would intuitively be expected, and statistical assistance should usually be sought before accepting any apparent interactions between treatment differences and patient characteristics as real.

(10) Failure to check really carefully that, on your selected stopping date, all the patients you think are alive really are alive is unwise; many trial organizers underestimate the time it takes for news of death to reach them.

(11) A " one-sided " or " one-tailed " *P*-value may be cited in a clinical trial report; if so, you should usually double it to get the sort of ordinary *P*-value which you are used to, and which would emerge from the methods given in this paper. If, in a trial, Group A fares better than Group B, then the probability of A doing at least this much *better* than B just by chance is the *one-sided P*-value, while the probability of the difference between A and B being at least this big *in one direction or the other* (A better or B better) just by chance is the ordinary *P*-value. (For emphasis, the ordinary *P*-value is occasionally referred to as the two-sided or two-tailed *P*-value.)

(12) Published *P*-values are sometimes calculated after excluding protocol deviants, or any other category of withdrawn patients; if so, they should be mistrusted. Section 13 in Part I discussed how and why a reliable analysis should be done in such trials. It is, however, sometimes useful not only to do a rigorous analysis, treating withdrawals *etc.* properly, but also to do various informal analyses omitting certain such patients, assuming certain of them to have died soon after loss (or to have lived for ever), and so on. If all these informal analyses agree with the rigorous analysis in some conclusion, it will make that conclusion more acceptable to many readers. (No disagreement can arise if, due to good trial design, there are few, or no, exclusions or withdrawals.) If the analyses do not all agree, the investigator should make sure he understands why they do not, but should usually trust the rigorous analysis more than the others.

(13) In Part I, we argued against the use of historical controls when randomized controls could be used instead. Although we asserted that most claims based on historically controlled studies remain open to reasonable doubt, it should not be inferred that all historically controlled studies are the same. Certain investigators appear to feel that any comparison can be made moderately respectable simply by labelling one group of patients " historical controls ", and so, when reading reports of historically controlled comparisons, one should always be aware of the possibility of gross errors of method. On the other hand, excellent investigators sometimes have to make cautious use of what are, effectively, historical controls, especially when the alternative would be no answer for months, for years, or for ever.

### 24.—*How much data should be collected from each patient?*

**The general principle is: collect as much data as possible at first presentation, only data which are strictly necessary thereafter, and analyse the data you do collect very thoroughly.**

Although not essential, the collection of extensive data on each patient *at the time of randomization* (including perhaps a serum sample, some biopsy material or some other biological matter*) which can be stored indefinitely in case analysis of it is required, can help check to what extent

* Stored samples from a large series of diseased patients can often be of great value when new hypotheses are devised in the future, especially since analytical results can then be immediately correlated with survival duration. Any measurement which is strongly correlated with survival for a reason which is not obvious is likely to have such a deep connection with the fundamental disease processes that elucidation of this is likely to prove really fruitful. This is much less likely to be true of a measurement which, although abnormal in diseased patients, is not strongly correlated with prognosis.

any treatment effects that do appear are due to the chance inclusion of an excess of good-prognosis patients on one protocol: in other words, such data can help our statistical analysis of the treatment effects to compare like with like. Such data may also enable us to identify particular subgroups in which one treatment is preferable, and anyway, the relationship of presenting features to each other or to prognosis in a uniformly treated series can sometimes be of more interest than the treatment comparison itself.

By contrast, apart from recording any significant side-effects of treatment, and recording the date and cause of death, plus perhaps the dates of a few other relevant events, data collected *after randomization* are of less value, and simplicity in what the participating physicians are asked to record during the follow-up of each patient should be sacrificed only for a very good reason.

Most routine data recorded during follow-up in many clinical trials are never used in any publication, and were collected partly because the trial designers had not thought out clearly what they would really need and what they would not. It is not easy to know in advance what will be needed, but the blunderbuss approach of demanding masses of details in case one or two items are eventually needed is wasteful, or worse. Excessive form-filling can be positively harmful, if it wastes so much time at the hospitals that gaps get left in some essential data, or if doctors become reluctant to enter patients into this, or some future trial, because of the administrative burden anticipated. It should be emphasized that, although extensive data will need to be recorded in the *hospital notes* of each patient during the follow-up, little of this need be sent to the trial organizer. Although the trial organizer need not try to understand the full clinical course of each patient, he must however know when whatever critical events (toxic manifestations, perhaps, relapse and death) he is interested in occur, and preferably he should know what circumstances immediately preceded or accompanied those events.

If the treatment should, if it is being effective, cause some observable effect, like leukaemia remission or solid tumour shrinkage, on the disease itself then an assessment of these effects should be made in each patient. Even if survival is not significantly different, there may be a highly significant immediate effect of one treatment which is of great interest.

Special studies of the progress of the disease can be added to the clinical trial at certain centres that wish to do so, and these may illuminate either the natural history of the disease or the mechanisms underlying certain treatment effects. However, in a multi-centre trial it may be wise to leave these extra data-recording tasks as optional activities which any participating centre can be free from, if it so wishes, without censure. In trials where the organizer feels he has to ask for some follow-up data, he should, soon after the trial has got properly under way, reconsider what data to request (or how emphatically to demand it) in the light of which requested items actually get very incompletely documented, how much work each item actually involves, and any other practical considerations that have arisen. If some changes are necessary or some work is unnecessary, the sooner this is recognized the better.

The causes (or circumstances) of death should definitely be recorded, if possible, and grouped into a few distinct categories. For instance, in myelomatosis we might try to separate deaths according to whether the patient died of:

(1) " myeloma kidney " during Year 1
(2) other causes during Year 1
(3) sudden onset of drug resistance *after* Year 1
(4) other, *after* Year 1.

It is obvious that the determinants of myeloma kidney and drug resistance may be completely independent of each other and that separate analyses of these

4 separate categories of death could be enlightening. Other subdivisions of mortality (*e.g.* whether neutropenia preceded death) could be useful for other purposes.

Two separate, but extremely important, ways in which retrospective enquiry about the events preceding each death may be of value are (i) that such enquiry may suggest new therapeutic strategies designed to prevent particular causes of death; and (ii) that such enquiry may strongly suggest that some preventable side-effect of one treatment is causing a few particular deaths, even though the number thus caused is far too small to be noticed in any comparison of overall mortality.

For example, in 1964, melphalan was only available in 2 mg tablets, the daily dose was difficult to adjust, and it was discovered that a few deaths of melphalan-treated patients were preceded by drug-induced neutropenia. Warnings, and the manufacture of 0·25 mg tablets, coincided with the cessation of such deaths. Likewise, in acute lymphoblastic leukaemia 10 years later, some deaths were found to be preceded by neutropenia. Review of the case histories of those who died showed recent radiotherapy in almost every case, and further studies then discovered that drugs and radiotherapy given in the particular time relationship which had unfortunately been used, caused far more neutropenia than their separate actions would suggest. Finally, the small and non-significant excess of early deaths in acute myeloid leukaemia patients treated with asparaginase was confidently attributed to the asparaginase, because only among asparaginase-treated patients had marked granulocyte depression apparently contributed to death.

### 25.—*Subdividing the follow-up period*

**Mortality may have different correlates during different phases of the natural history of the disease, and these should be sought separately.**

Splitting the time after follow-up into about 3 different periods, and comparing the logrank O with E in each period separately, as well as in all 3 added together, is a useful safeguard against misinterpreting situations such as those illustrated in Fig. 6, where one treatment is only better than another in certain periods. Fig. 6(i) is an ordinary situation, where A would be better than B in each

FIG. 6.—Three hypothetical ways in which treatments might differ in their effects on survival: for each, % alive is plotted against number of years since randomization separately for treatments A and B.

year. Fig. 6(ii) is a situation where A would be better than B in the first and second years, with no treatment comparison possible in the third year, since there are almost no B survivors to compare with the A survivors. Fig. 6(iii) depicts a situation which is often worried about, and occasionally encountered, where the treatment which is initially better is actually worse in the long run. The logrank test for data such as those in Fig. 6(iii) would show that the death-rate among A survivors was better in the first year, *worse in the second year*, and worse in the third year. In Figs. 6(i) and (ii) the overall logrank test would necessarily show A to be better than B overall, but in Fig. 6(iii) any result could emerge from the overall logrank test (A better overall, B better overall, or no difference overall).

Suppose that an intensive treatment, which might well kill a substantial number of patients early in the trial, is to be compared with a gentler treatment, which is probably not as fatal in the short run but which may be less curative in the long run, producing the pattern illustrated in Fig. 6(iii). Here, it would be sensible to subdivide time from randomization into an initial period (during which most of the deaths from the intensive treatment would be expected), and a later period, and to accumulate (O–E) for each treatment in the initial period only and (O–E) for each treatment in the later period only, examining these separately. The split between " early " and " late " should be chosen either soon after the intensive treatment becomes less intense, or from consideration of the overall survival curve for all patients together to find when the overall death rate changes. The split should not, of course, be chosen after examination of the 2 separate survival curves for the 2 treatments, for it is too easy to find a time when one treatment has a temporary advantage over the other. Subdividing time in this way is also good policy when the nature of the disease changes as time goes by, *e.g.* from initial crisis to remission maintenance, as the determinants or correlates of mortality in one period may differ from those in the other. Again, the *overall* survival curve could be used as an unbiased guide on where to split time to separate an early period of rapid death from a later period.

26.—*Arranging the manner in which data will be collected*

**A controlled clinical trial is a substantial research undertaking, and sufficient time and money must be set aside to ensure that the records are always complete and up-to-date.**

(1) Make sure that the (extensive) initial data from each patient are collected centrally and checked for completeness immediately the patient is entered, so that missing, obscure or unreadable items can be corrected quickly. Retrospective efforts to supply missing data or check implausible items can be very difficult, and can seriously delay the analysis of the trial. If some data are missing, however, it is (unfortunately!) worth making considerable efforts to collect them, if there is any possibility of doing so, as missing data make the statistical analysis much more difficult, and may make the trial report less easy for other workers to interpret without serious doubts about certain of its conclusions.

(2) If something is assessed by a number, record that number exactly, even though the margin of error may be very wide, and not some rounded or grouped version of it. (*E.g.* it is better to record haemoglobin exactly than to use it to categorize patients as anaemic/not anaemic, and it is better to record cigarette consumption exactly than to divide patients into ranges such as 1–4, 5–14, 15–25, *etc.*) It is easier to devise appropriate groupings of numerical data when analysing than when designing the trial. Record dates of birth exactly (not just age), as this is sometimes very useful for tracing lost patients, especially if use may be made of official government records.*

(3) By contrast, if something can be assessed only subjectively (*e.g.* general condition of patient, nature of tumour, previous history, reasons for presentation, *etc.*), it is usually worth forcing, albeit slightly artificially, the physician recording the data to do so in certain pre-specified categories (*e.g.* good, fair, bad; well-differentiated, intermediate, undifferentiated). Although categorization is not helpful when describing an individual case history to a colleague, it is essential for describing a group of case histories, and categorization will either be done with some loss of precision by the physician examining the patient, or it will be done later with a greater loss of precision by somebody examining the medical records of that patient.

For example, the Eastern Co-operative Oncology Group simply record " performance status " as 0–4, where 0 = normal activity, 1 = symptoms but ambulatory, 2 = in bed less than half the time, 3 = in bed more than half the time, and 4 = completely bedridden. Zelen finds that if advanced cancer patients are divided with respect to performance status, the median survival times are very different. Although this may not be a very useful finding biologically, it does mean that if this simple question is asked of all patients on entry to a therapeutic trial in advanced cancer, the treatment comparison in that trial will be more accurate. (By retrospective stratification based on this information, we can get closer to the ideal of comparing like with like.) An alternative performance scale is described in Zelen (1973), where among about 1000 patients with inoperable lung cancer " performance status " was more important than histological type, disease extension or any of the usual information!

Usually, categories containing very few patients are not useful, and have to be merged with other categories, so do not create such categories unless there are strong medical reasons for doing so.

(4) If, in a clinical trial of over 100 patients, you choose to record a lot of presenting features, it is probably worth obtaining programming assistance and getting a convenient computer program set up before starting the statistical analysis, so that any particular tabulation of presenting features against each other or against prognosis can be obtained easily. Otherwise, the data will not receive the attention which, in view of the cost of such trials (Pike, 1973), they deserve. The " SPSS " system, which is available on most large computers, is of some value, but unfortunately it does not implement the logrank test. A FORTRAN program is available which is easy to use and capable of calculating and displaying life tables, performing logrank tests, devising strata, and analysing the effects of one factor (*e.g.* treatment) making full allowance for some others (*e.g.* the important explanatory variables). Copies of this will be supplied on request,† at a slight charge to cover duplication and postage.

(5) If the data may eventually be

* In the UK, a clinical trial organizer may write to the Registrar-General, Department of Medical Statistics, Office of Population Censuses and Surveys, St Catherine's House, 10 Kingsway, London W.C.2, giving his credentials as a *bona fide* research worker plus a very brief outline of the trial, asking the R-G for help in monitoring the dates of death of all trial patients who are normally resident in the UK. If the R-G agrees, and is later supplied with the full name and exact date of birth of each trial patient (either in one batch, after intake has ended, or in a few batches as it progresses), together with a fee of nearly £1 per patient, then the R-G will notify the trial organizer whenever any deaths occur, giving the date and certified cause of death, and the name of the physician who certified the death. (The trial organizer will be notified immediately if any of his batch of patients are already dead.) Adequate follow-up of survival is thus much easier in Britain than elsewhere, for the R-G will usually agree to help any reasonable project by *bona fide* medical research workers, especially if recent addresses or NHS numbers can also be supplied by the trial organizer for most patients.

† P. G. Smith, DHSS Cancer Unit, 9 Keble Road, Oxford, England.

transferred onto a computer, they should be recorded in such a way that they can be transferred with a minimum of trouble. Notes on how to do this constitute Appendix 4.

27.—*Assessment by separate causes of death*

**Life tables and logrank tests may easily be used to study separately different causes of death.**

If the effect of a presenting feature on the difference between 2 treatments is likely only to affect one (or a few) causes of death, we may choose to look separately at those causes of death which are considered relevant. The previous methods of analysis carry over exactly to this situation, the numbers at risk on each day being just as before, although the extent of exposure to risk of death from a relevant cause on a particular day is related, not to the total number of deaths on that day, but to the total number of *relevant* deaths on that day. Likewise, in calculating the life table, we calculate for each day the proportion who do not die of one of the *relevant* causes, and then combine these proportions, as in Section 18, to obtain the life table description of mortality from *relevant* causes only. (This is equivalent to treating deaths from other causes as if they were losses to follow-up, assuming that the different causes of death being studied are independent. Unfortunately, this assumption cannot be tested statistically, but must instead be judged reasonable or not on biological grounds.) For example, in the current MRC polycythaemia trial, which may last for 10 years, we may exclude from certain analyses deaths from causes that are unlikely to be related to polycythaemia or its treatment.

Actually, we shall calculate separately the observed numbers of *relevant* deaths and the extents of exposure to risk of *relevant* death by treatment group, and the observed number of *non-relevant* deaths and the extents of exposure to risk of *non-relevant* death in each treatment group, so that readers can look either at relevant mortality or at total mortality: observed numbers of deaths from relevant and non-relevant causes can be simply added together to give total numbers of deaths, and so can extents of exposure to risk of relevant and non-relevant death. Separating a few major causes of death, and calculating in each treatment group the observed numbers of deaths from each cause, and extent of exposure to risk of death from each cause, can help the interpretation of data from clinical trials of slowly progressive diseases. Again taking an example from the polycythaemia trial, we may also examine separately (a) the deaths due to onset of acute leukaemia, (b) the deaths due to marrow fibrosis, and (c) other deaths, to see whether active cytotoxic treatment is more leukaemogenic than simple venesection.

28.—*Other end points*

**Life tables and logrank methods can find whether, among survivors on a given day, any particular endpoint is more likely in one group than another; this is very useful, but can be misused.**

It may be of use to note that these methods of analysis can as easily be applied to the influence of treatment on events other than death: time to first detection of leukaemia in the central nervous system; time to first cardiovascular event (fatal or not) and so on. In many clinical trials studying solid tumours, 2 separate analyses, one of time to local recurrence and one of time to metastatic spread, may be required in addition to a full analysis of survival duration.

Exactly analogous statistical techniques may be used for any such analyses, except that now we are interested in the time from randomization to the first such "event" instead of to death. Consequently, patients contribute no further information to such an analysis once their first "event" has occurred. The only difficulty is to decide how to deal with patients who die without suffering the event of interest.

If the event is such (*e.g.* neoplastic recurrence) that few patients die without previously suffering it, the most satisfactory course, for reasons which will soon become clear, is undoubtedly to define our interest as being in " recurrence or death not preceded by recurrence ". This means that our life tables will estimate the proportions of *recurrence-free survivors* at different times, and our O's and E's will test which treatment best prolongs *disease-free survival.*

If, however, the event is such that more patients die without suffering it than ever suffer it, study of it might be swamped if we mixed in all the patients who died without it. An example of this will arise in the current MRC trial comparing venesection with venesection plus cytotoxic treatment for polycythaemia: whether or not any significant difference in total mortality emerges, we shall want to know if, compared with simple venesection, the cytotoxic treatment is leukaemogenic. Counting the few patients who develop leukaemia (including leukaemias which are fatal before the analysis is undertaken and leukaemias which are not) in each group is easy. Calculating the extent of exposure to risk of leukaemia is also easy: we simply argue that on a given day after randomization, all patients who are still alive and free of leukaemia would, if cytotoxic treatment were not leukaemogenic, be equally likely to develop leukaemia. The only difference from our original analysis arises when counting the numbers of patients still alive and at risk on a particular day after randomization (in order to calculate the proportion of them that are in a particular group). Now, we must omit not only, as before, those patients who entered too recently for us yet to know their fate on this day after randomization, but also those who have already developed a leukaemia before this day. This is easy: for purposes of the statistical analysis of leukaemia incidence, re-define the trial time, for patients who eventually get leukaemia, as the time from randomization to leukaemia diagnosis. (For patients who did not get leukaemia, the trial time runs as before to death or, if not dead, to the stopping date or date of loss.) Now the extent of exposure to risk of leukaemia in Group A on a particular day after randomization is the total number of patients in the whole study who get leukaemia on that day, multiplied by the proportion of patients with trial times at least that long who are in Group A.

Once the underlying principle of asking " Among the event-free survivors on a particular day after randomization, are people in each group equally likely to suffer an event on this day? " is mastered, its applications can be very varied and very useful: the method automatically makes perfect adjustment for the effects of differences in mean duration of risk in each group (and for the effects of differences in risk at different times after randomization) on the expected number of events in each group.*

Another example of the use of these methods to search for a particular end-point, was the proposed MRC trial of oral hypoglycaemic agents in diabetes, where the main question of interest would have

* There is, however, one possible source of error which may arise if some patients die without being observed to have suffered the unwanted " event ". Those who die are never a random sample of those who remain, usually because their disease state was more severe, but sometimes (causing severe bias) because causes which would have culminated in the untoward event whose incidence we wish to study actually led to death, just before the relevant event was observed. In either case, delay of death by an effective treatment may just allow the relevant event to be observed and counted against that treatment! Use of the logrank test on such events would correctly indicate that, among the survivors on a given day after randomization, patients given a more effective treatment would be more likely to be observed to suffer such events, although this would be a very misleading fact.

For example, if leukaemic proliferation in the blood causes contamination of the central nervous system been their effect on vascular disease. We would have counted the observed numbers of people in each group who suffered a first vascular event after randomization, and compared these with the calculated extents of exposure to risk of suffering one. (Analysis of the *total* number of vascular events in each group would be less satisfactory, as the chance inclusion of one patient who suffers several events might affect the totals unduly.)

If there are 2 or more definite events that might be adopted as "endpoints" (*e.g.* time to first rejection episode *and* time to complete graft failure, in the MRC trial of therapy following renal transplantation, or time to relapse *and* time to death in leukaemia), then it is usually worth making a completely separate analysis of the whole trial for each endpoint of interest.

### 29.—*Remission duration*

**If time is measured from the date of remission, analogous analyses of remission duration are possible.**

Exactly analogous techniques can be used to study the duration of remission, if the trial time is taken to start at remission and to end at "relapse or death without relapse" rather than at death. If the randomization is done before the state of remission is achieved, the time at which the physician declares that a patient is in remission may depend on the particular treatment regime that the patient will receive in remission, and this could bias the results. It is therefore preferable, if possible, not to randomize until remission has been achieved, and then to study time from randomization, as before. If this is not possible, a very strict definition of remission and relapse is needed in order to communicate the results to other workers and to avoid any possibility of biased assessment. It is a general rule that randomization should always be left to the last possible moment before the start of treatment, so that events between randomization and treatment do not bias or obscure the effects of the treatments.

Moreover, it should always be borne in mind in analyses of remission duration, that a treatment may prolong the first remission without prolonging survival (as in the immunotherapy trial described in the next section) if the delayed relapse is more likely to be refractory to treatment. (Against this, the observation that "first remission duration and survival duration are strongly correlated" is sometimes made, which, although true, is not relevant in this context.) It should also be borne in mind that a good treatment, which improves the chances of achieving remission but does little or nothing to prolong remission, may result on average in shorter remissions among those achieving remission, because more of the very ill patients are being got into remission and they might relapse more rapidly.

### 30.—*Combining information from different trials*

**Different trials which each compare the same 2 treatments may best be pooled, to give an overall treatment comparison, by analysing the data as though the separate trials were each retrospective strata within one large trial.**

Some therapeutic comparisons which are important, but which are nevertheless known not to involve major differences in survival, require larger resources than any of the investigators who have

(CNS) which is followed by proliferation in the CNS, partial control of the blood but not of the CNS would prevent death from leukaemia in the blood but would allow CNS relapse to be seen. In this case a logrank analysis of the event "CNS relapse or death without it" might be less misleading than a logrank analysis of "CNS relapse", but unfortunately this safer analysis would be less sensitive, if one treatment really did just prevent or exacerbate CNS relapse, and would moreover confuse control of CNS relapse with control of death from other causes. The same difficulties arise, of course, with all other statistical techniques, including the apparently more straightforward visual examination of life tables.

approached them have mustered; and consequently many different randomized trials of these treatment comparisons may have been undertaken, none of which is sufficiently accurate on its own. An example might be the possible utility of anti-coagulants following myocardial infarction, or the utility of a particular form of immunotherapy for a particular neoplasm. We might hope to find an overall tendency for patients given A to fare slightly better than patients given B, obscured or enhanced in each particular trial by random variation.

The most efficient unbiased way of determining whether this is so is to ignore in each particular trial all patients allocated to treatments other than A or B, and then to calculate, for each separate trial, O and E for Treatment A and O and E for Treatment B. Finally, we combine the trials by adding all the O's for Treatment A, to get an overall $O_A$, all the E's for Treatment A, to get an overall $E_A$, and similarly we obtain a combined $O_B$ and $E_B$: $O_A + O_B$ will equal $E_A + E_B$, of course. Comparison of $O_A$ with $E_A$ and $O_B$ with $E_B$ in the usual way, by computing $X^2 = (O_A-E_A)^2/E_A + (O_B-E_B)^2/E_B$ and using the analogy between $X^2$ and chi-square with one degree of freedom, will lead to a *P*-value testing whether A and B are statistically significantly different from each other. Calculation of $R = (O_A/E_A)/(O_B/E_B)$ will, moreover, yield a useful pooled estimate of the ratio of the death rate on A to that on B.

This method of pooling different trials treats each trial as though it were a retrospective stratum in a single large trial, and of course would be equally applicable if some of the separate trials were themselves actually subdivided, *e.g.* into young patients and old patients, with calculation of the O's and E's comparing treatments occurring entirely within separate strata in each separate trial. (A standard alternative is to pool different trials by mathematically combining the *P*-values from the separate trials, but this is less sensitive.) It is an advantage of reporting logrank O's and E's when publishing clinical trials, that the efficient combination of different studies is then straightforward.

### EXAMPLES

31.—*Example I. Immunotherapy of acute leukaemia*

In 1968, Mathé and his co-workers reported that of 20 patients with acute lymphoblastic leukaemia in remission who were subsequently given immunotherapy, 8 achieved long-term remissions, whereas none of an untreated control group of 10 such patients did so (Mathé *et al.*, 1969). Eight of the 20 patients treated by immunotherapy had received BCG alone, and it appeared that this was as effective as the other immunotherapy regimens of blast cells alone or BCG combined with blast cells. Animal work had shown that in mice BCG can, under certain circumstances, cure grafted isogeneic leukaemias, and it was hoped that this might be the first news of the breakthrough to a real leukaemia cure. However, approximate comparison with contemporary British experience suggested that, although the treated patients had fared fairly well, the controls had fared much worse than was normal, and that this accounted for much of the disparity between the 2 treatment groups. It was therefore decided that the Medical Research Council should organize a clinical trial to assess these claims made for BCG. If BCG has any effect, first remissions in which BCG is given regularly should on average last longer than first remissions in which no treatment is given; and the trial as originally conceived was to compare unmaintained and BCG-maintained first remissions.

The intake to the trial was to consist of children with acute lymphoblastic leukaemia who had undergone a standard 5-month course of intensive cytotoxic therapy from their time of diagnosis. After this course, maintenance therapy with methotrexate was expected to prolong the first remission, but possibly to make relapses, when they occurred, more refractory than if no maintenance treatment had been given. (Some leukaemic relapses occurring during unmaintained remission can be controlled by drugs, while others are refractory. If the effect of maintenance treatment during remission is chiefly to suppress those relapses which, had they occurred, could have been controlled anyway, then only the refractory relapses will break through. This effect must be allowed for in the comparison of drug maintenance with immunotherapy-only maintenance, before claims that " immunotherapy in remission facilitates the control of subsequent relapses " can be made.) It appeared, therefore, an open question whether a patient in remission should be left alone or given maintenance chemotherapy.

However, some physicians felt unhappy about not including a maintenance methotrexate group in the trial, and the trial as finally agreed, therefore, had 2 " control " groups, a no-treatment group and a methotrexate group. At each centre, 2-way randomization between immunotherapy and control was used, but which regimen to use for all their control patients was chosen by each centre before the trial began. Intake began in January 1969 and continued to August 1970.

An individual trial cannot exist in isolation. It will of necessity last some years, even if intake only lasts one year, and during this time reports from other workers may make the trial irrelevant, and may even make its continuance unethical. This is a major problem, and must be considered very carefully before starting a trial (Pike, 1973). About one year after the start of this trial, reports from the United States showed that the extent to which first remission was prolonged by maintenance methotrexate was far greater than had been supposed. The physicians from centres which had chosen the no-treatment control now felt that they should no longer put patients into this group. It was therefore decided that all control patients entered from that time onwards should receive maintenance methotrexate as their control treatment. This detracted from the uniformity of the series of patients, but was in the circumstances unavoidable.

One advantage of the fact that 5 months of standard cytotoxic therapy preceded the allocation to different treatment groups in this trial was that all the " difficult " patients (those who had " bad " veins, refused in-patient treatment, were unable to tolerate the intensive therapy, or were not in remission 5 months after first treatment) were eliminated from the trial before randomization. The group available for randomization was therefore more than usually homogeneous: this generally has the effect of increasing the chances of detecting any differences between the treatments. This uniformity was, however, marred to some extent by the dose of L-asparaginase given in the intensive treatment phase being reduced after the first 50 patients, as the original dose level was proving too toxic and too expensive. This could perhaps have been avoided, had the protocol been tried out more extensively on a pilot basis (MRC, 1971*b*) just to study its toxicity.

Because of the great interest generated by Mathé's group's results, the trial was analysed at several different times. As we have explained, this is in principle a bad practice, but in this case it was inevitable. These analyses were initially limited to the overall comparison of the 3 treatment groups. To our disappointment, it was soon apparent that the effect of BCG was, if positive at all, only slight. However, since our BCG therapy protocol differed from the French in possibly important ways, this finding is of less value than it might otherwise have been.

Ideally, one should use an identical protocol if one wants to test a published claim, but this was not possible, partly due to difficulty in obtaining the Pasteur Institute vaccine used by the French; partly due to unwillingness to scarify large areas of skin, as they had done, rather than use percutaneous inoculation by Heaf gun; and partly because 7 different protocols were used by the French, each on 2, 3 or 4 of the immunotherapy patients. In retrospect, differences between the MRC protocol and the French protocols meant that the negative result eventually found in our trial is not sufficient to demonstrate that their methods were not effective. However, another, still larger, trial (Heyn *et al.*, 1975) based on Mathé's report has also produced a null result.

In the MRC trial, 191 cases presented, 122 went into remission and were randomized as intended at Month 5, and a further 10 were randomized later, during the next few months. When, in 1971, half the patients had relapsed (though few had died), the results among the 122 were reported (MRC, 1971*b*). People who have cited this publication have given less prominence to the fact that there was no statistically significant difference between the first remission durations of the group given BCG and those of the untreated control group, than to the fact that the observed median remission duration, an unreliable statistic, was 27 weeks on BCG and 17 weeks on untreated control. However, *partly because this difference diminished as more data accumulated*, the remission durations have never been properly reported since. The trial was not a critical test of immunotherapy, because prophylactic treatment of the central nervous system was so inadequate that meningeal relapses prematurely terminated many of the remissions, but since on 1 January 1976 only 3/55 children given immunotherapy were still in first remission (compared with 2/20 given no maintenance and 8/57 given methotrexate) BCG given in this way appears to be of little value (Table VI).

In Table VI, the fourth line confirms that there is little difference between BCG and untreated control, since the relative relapse rates are so similar (1·25 and 1·32).

If the data for relapses in the BCG and untreated control groups are pooled, the combined relative relapse rate is 1·27: (52 + 18)/(41·58 + 13·61). The relative relapse rate on methotrexate is, by comparison, only 0·77, confirming that maintenance methotrexate really did pro-

TABLE VI.—*MRC Immunotherapy ("Concord") Trial: Follow-up on* 1 *January* 1976

| | BCG maintenance (55 patients) | | | Untreated control (20 patients) | | | Methotrexate maintenance (57 patients) | | | Total (132 patients) | |
|---|---|---|---|---|---|---|---|---|---|---|---|
| | O | E | O/E | O | E | O/E | O | E | O/E | O | E |
| First relapse or death in first remission | | | | | | | | | | | |
| (a) WBC* 0–5 | 18 | 11·56 | 1·56 | 6 | 3·62 | 1·66 | 17 | 25·82 | 0·66 | 41 | 41·00 |
| (b) WBC* 6–20 | 18 | 14·02 | 1·28 | 7 | 5·72 | 1·22 | 15 | 20·27 | 0·74 | 40 | 40·00 |
| (c) WBC* 21+ | 16 | 16·01 | 1·00 | 5 | 4·28 | 1·17 | 17 | 17·71 | 0·96 | 38 | 38·00 |
| Total (a) + (b) + (c) (*i.e.* numbers of relapses and total extents of exposure to risk of relapse stratified retrospectively for WBC) | 52 | 41·58 | 1·25 | 18 | 13·61 | 1·32 | 49 | 63·80 | 0·77 | 119 | 119·00 |
| Death | | | | | | | | | | | |
| Numbers of deaths and total extents of exposure to risk of death within WBC strata | 39 | 39·46 | 0·99 | 15 | 15·05 | 1·00 | 40 | 39·49 | 1·01 | 94 | 94·00 |

* White blood count at presentation, in units of $10^9$/l. Among people in full remission, the original WBC is still a good indicator of how long the remission might last, and among people diagnosed years ago, the original WBC is a useful predictor of future risks of death.

long first remissions ($\chi^2 = 7{\cdot}41$, d.f. = 1, $P < 0{\cdot}01$), a result which was reported in the 1971 paper. However, examination of the life tables (not given) and the last line of Table VI, where the relative death rates are all unity, shows that, in the long run, the group given methotrexate maintenance did not survive any longer. This result has so far not been reported, partly because the treatments given after relapse were so various as to defy summary, but also, unsatisfactorily, because it is null.

An interesting feature of the effect of methotrexate on relapses is that the lower the initial WBC, the greater the importance of methotrexate. The 3 ratios of the relative relapse rate of the methotrexate patients to the combined relative relapse rate among BCG plus unmaintained control patients in Table VI, were 0·42 (WBC 0–5), 0·58 (WBC 6–20), and 0·93 (WBC 21+). In other words, among patients presenting with lower WBCs, methotrexate can apparently roughly halve the relapse rate, while among patients presenting with high WBCs, methotrexate seems to have almost no delaying effect on relapses. When, as here, the relative merits of 2 treatments are very different in different strata, there is said to be an *interaction* between strata and treatments. This particular interaction has been noticed in other studies, and deserves investigation.

32.—*Example II. The MRC myelomatosis trials*

By 1964, it was generally agreed that both cyclophosphamide and melphalan were useful drugs for treating patients suffering from myelomatosis. No randomized comparison of them had, however, been made and a trial to compare daily oral administration of the 2 agents was begun in October 1964 by the Medical Research Council. Intake continued until August 1968, by which time 276 patients had been admitted. The 2 treatment groups fared almost exactly the same, and apart from this, the main scientific interest of the trial has, therefore, been the relationship between certain biochemical measurements made on the patients at admission and the survival times (MRC, 1973).

It was known that a high blood urea indicated renal failure, and was the major determinant of prognosis, but the statistical techniques discussed above enabled us to investigate a whole range of possible explanatory factors. We found that the apparent adverse effects of hypercalcaemia, osteolytic lesions, and the presence of Bence Jones protein in the urine, were wholly explained by their associations with uraemia. The degree of initial anaemia was strongly correlated with prognosis, and as expected, this correlation was by no means completely accounted for by the strong association between uraemia and anaemia. However, one entirely unsuspected factor—the serum albumin—was found to be of substantial prognostic significance, independently of the urea level (Table VII).

This table is based on 258 of the total of 276 patients entered (18 patients with unusual paraprotein types were excluded from this analysis). The difference between the relative death rates of 1·3 and 0·4 for the low and high albumin patients with no evidence of renal failure, represents a difference between about 18 months and 5 years in median survival time, and is thus of considerable medical significance. The reason for this difference remains obscure. It has been suggested that it might be that the more dangerous myeloma cell populations actually catabolise albumin much more rapidly, but this suggestion has not been properly investigated. Likewise, the reason for the relevance of anaemia to prognosis requires investigation, to see whether the relevant anaemia is chiefly of renal origin or due to bone marrow failure.

In the second MRC myelomatosis trial, daily low-dose cyclophosphamide was compared with intermittent high

TABLE VII.—*First MRC Myelomatosis Trial*

Relative death rates by initial serum levels of urea and albumin

| | Low albumin | Medium albumin (30–39 g/l) | High albumin | All albumin levels |
|---|---|---|---|---|
| Low urea (no evidence of renal failure) | 1·3 | 0·8 | 0·4 | 0·6 |
| Medium urea (40–79 mg/100 ml) | 1·8 | 1·0 | 0·9 | 1·1 |
| High urea (evidence of severe renal failure) | 3·8 | 4·4 | 2·1 | 3·2 |
| All urea levels | 1·8 | 1·1 | 0·7 | 1·0 (necessarily) |

doses of melphalan, and with intermittent high-dose melphalan plus prednisone. Because 3 treatment alternatives were being compared, a large number of patients was required, and intake lasted from 1968 to 1975, which is an undesirably long time. Three hundred and seventy-three patients were followed up to 1 July 1976. No significant difference between the 3 treatments emerged, but the intermittent melphalan plus prednisone group did fare a little better. Since intermittent melphalan plus prednisone is as acceptable as intermittent melphalan alone, and more acceptable than continuous cyclophosphamide, it can be definitely recommended, even though its superiority was not statistically significant.

In the second trial, we again found a statistically significant relationship between albumin and prognosis, but it was a weaker relationship than had been observed in the first trial. This slight disappointment should have been anticipated; moderately strong relationships are often more extreme in the studies where they were first discovered to be interesting than in subsequent studies. This is because a relationship which is in expectation only moderate may, in various different studies, appear weaker than it should or stronger than it should, and it is the latter studies which generate interest. (For the same reason, promising new treatments should not be expected to live up to their early promise.)

We now have extensive initial data, and various numbers of years of follow-up, on a total of over 600 myeloma patients, all treated fairly equivalently. Some of the associations with prognosis (particularly that of anaemia) are proving easier to investigate in this larger series than in the first trial series alone. As far as prognostic correlates and their interpretation are concerned, though, the chief need now is not for still larger numbers, nor for other workers to check our findings on other, probably smaller series, but for new and preferably strikingly different therapeutic strategies to be proposed and tested, and for new factors to be measured in future patients. Since 1975, these have been the aims of the third MRC myelomatosis trial.

M. C. Pike is supported by Contract NO1-CP-53500 and Grant PO ICA 17045-02 from the National Cancer Institute, and N. Mantel by PHS Grant CA 15686.

We are grateful to Gale Mead for typing the annual rewrites which this manuscript has suffered since 1971, and to the many colleagues who used and criticized previous versions.

The Statistics Department, University College, London, authorized our reproduction of Table III from Tracts for Computers, No. 24. R. Peto is supported by an Imperial Cancer Research Fund readership in cancer studies.

REFERENCES

ARMITAGE, P. & GEHAN, E. A. (1974) Statistical Methods for the Identification and use of Prognostic factors. *Int. J. Cancer*, **13**, 16.

BRESLOW, N. E. (1975) Analysis of Survival Data under the Proportional Hazards Model. *Int. statist. Rev.*, **43**, 45.
COX, D. R. (1972) Regression Models and Life Tables (with discussion). *J. R. statist. Soc.*, *B*, **34**, 187.
GEHAN, E. A. (1965) A Generalized Wilcoxon Test for Comparing Arbitrarily Singly-censored Samples. *Biometrika*, **52**, 203.
HEYN, R. M., JOO, P., KARON, M., NESBIT, M., SHORE, N., BRESLOW, N., WEINER, J., REED, A. & HAMMOND, D. (1975) BCG in the Treatment of Acute Lymphocytic Leukaemia. *Blood*, **46**, 431.
KAPLAN, E. L. & MEIER, P. (1958) Nonparametric Estimation from Incomplete Observations. *J. Am. statist. Ass.*, **53**, 457.
MANTEL, N. (1966) Evaluation of Survival Data and Two New Rank Order Statistics Arising in its Consideration. *Cancer Chemother. Rep.*, **50**, 163.
MATHÉ, G., AMIEL, J., SCHWARZENBERG, L., SCHNEIDER, M., CATTAN, A., SCHLUMBERGER, J. R., HAYAT, M. & DE VASSAL, F. (1969) Active Immunotherapy for Acute Lymphoblastic Leukaemia. *Lancet*, i, 697.
MEDICAL RESEARCH COUNCIL (1968) Chronic Granulocytic Leukaemia: Comparison of Radiotherapy and Busulphan Therapy. *Br. med. J.*, i, 201.
MEDICAL RESEARCH COUNCIL (1971*a*) Myelomatosis: Comparison of Melphalan and Cyclophosphamide Therapy. *Br. med. J.*, i, 640.
MEDICAL RESEARCH COUNCIL (1971*b*) Treatment of Acute Lymphoblastic Leukaemia. *Br. med. J.*, **4**, 189.
MEDICAL RESEARCH COUNCIL (1973) Report on the First Myelomatosis Trial. *Br. J. Haemat.*, **24**, 123.
MEDICAL RESEARCH COUNCIL (1974) Treatment of Acute Myeloid Leukaemia with Daunorubicin, Cytosine Arabinoside, Mercaptopurine, L-asparaginase, Prednisone and Thioguanine: Results of Treatment with Five Multi-drug Schedules. *Br. J. Haemat.*, **27**, 373.
PETO, R. (1972) Rank Tests of Maximal Power against Lehmann-type Alternatives. *Biometrika*, **59**, 472.
PETO, R. & PETO, J. (1972) Asymptotically Efficient Rank Invariant Test Procedures (with discussion). *J. R. statist. Soc.*, A, **135**, 185.
PETO, R. & PIKE, M. C. (1973) Conservatism of the Approximation $\Sigma(O-E)^2/E$ in the Logrank Test for Survival Data or Tumor Incidence Data. *Biometrics*, **29**, 579.
PIKE, M. C. (1973) The Analysis of Clinical Trials in Leukaemia. In *Recent Results in Cancer Research*, **43**, Ed. G. Mathé, P. Pouillant, and L. Schwarzenberg. New York: Springer-Verlag.
ZELEN, M. (1973) Keynote Address on Biostatistics and Data Retrieval. *Cancer Chemother. Rep.*, **4**, 31.

### APPENDICES

### APPENDIX 3.—*Worked Example of a Clinical Trial Analysis (Hypothetical Data)*

Suppose that for each patient, we know the date of randomization, the date of death if he died before the follow-up date of 31 May 1974, the randomized treatment (A or B), and the renal function (I = impaired, or N = normal) at the time of randomization. Some hypothetical data of this type are set out in Table VIII, along with the derivation from them of the trial time. We shall study the relevance of treatment to death from the disease or from causes that might well be correlated with the disease or its treatment, and so we shall not include the death due to a road accident in our analysis. If we wanted to study total mortality, of course, we could easily count this one death and modify the calculated extents of exposure to risk on Day 2240 after randomization accordingly.

#### *Notes on the calculation of trial times in Table VIII*

(a) If a patient dies after randomization, but before treatment could be started, that is irrelevant: include him as though he had been treated. (To avoid such occurrences, do not randomize until the last possible minute.)

(b) If a patient changes treatment for serious medical reasons, for social reasons, or even at a doctor's whim, that is also irrelevant: include him as though no deviation had occurred, and try to ensure that as few such changes as possible occur in the next clinical trial you design. The anaylsis will then try to answer: "Is it better to adopt a *policy of* Treatment A if possible, with deviations if necessary, or a *policy of* Treatment B if possible, with deviations if necessary, for patients who *seem to* have this disease?" This is a relevant question, sometimes even more relevant than "Is A or B better for this disease?"

(c) If, during the course of the treatment, it is realized that the original diagnosis was erroneous, it may be decided that such patients should stay in the analysis as if no mistake had occurred (see the discussion surrounding Fig. 2 on p. 602 in Part I of this report), even if treatment has to be completely altered to attack the real disease.

(d) If causes of death are available for *all* patients who die, it may be decided that deaths from causes which could not possibly be related to the disease or its treatment will be ignored, and that such patients will be

TABLE VIII.—*Original Data, with Calculation of the Trial Times (Using* 31 *May* 1974 *as the Follow-up Date)*

| Patient number (arbitrary) | Treatment | Renal function at randomization | Date of randomization (day, month, year) | Date of death | Was an event suffered? | Trial time (days from randomization) | Note |
|---|---|---|---|---|---|---|---|
| 1 | A | I | 12.05.68 | Died before treatment could be started 20.05.68 | Yes | 8 | (a) |
| 2 | B | N | 18.10.70 | Died 16.04.71 | Yes | 180 | |
| 3 | B | N | 12.02.69 | Died 06.11.70 | Yes | 632 | |
| 4 | A | N | 30.01.72 | Still alive 31.05.74 | No | 852 | |
| 5 | A | I | 11.11.73 | Died 02.01.74 | Yes | 52 | |
| 6 | B | N | 12.03.68 | Died in road traffic accident 30.04.74 | No | 2240 | (d) |
| 7 | A | N | 06.01.69 | Died 14.08.69 | Yes | 220 | |
| 8 | A | I | 07.09.73 | Died 09.11.73 | Yes | 63 | |
| 9 | B | N | 02.05.71 | Died 13.11.71 | Yes | 195 | |
| 10 | B | N | 08.03.68 | Died 23.05.68 | Yes | 76 | |
| 11 | B | N | 12.12.73 | Died 20.02.74 | Yes | 70 | |
| 12 | A | N | 01.05.74 | Died 09.05.74 | Yes | 8 | |
| 13 | B | I | 02.07.72 | Died 15.07.72 | Yes | 13 | |
| 14 | B | N | 18.12.68 | Changed treatment from B to A on 18.12.69; still alive 31.05.74 | No | 1990 | (b) |
| 15 | A | N | 01.01.69 | Still alive 31.05.74 | No | 1976 | |
| 16 | B | I | 02.09.73 | Died 20.09.73 | Yes | 18 | |
| 17 | B | N | 11.02.70 | Diagnosis revised due to data obtained during 1971; died 12.01.72 | Yes | 700 | (c) |
| 18 | A | N | 12.11.70 | Still alive 31.05.74 | No | 1296 | |
| 19 | A | N | 19.05.68 | Lost to follow-up about 4 years after randomization | No | 1460 | (e) |
| 20 | B | N | 18.07.73 | Died 13.02.74 | Yes | 210 | |
| 21 | A | I | 12.03.69 | Died 14.05.69 | Yes | 63 | |
| 22 | A | N | 11.10.70 | Still alive 31.05.74; died Aug. 1974 | No | 1328 | (f) |
| 23 | B | N | 17.11.69 | Died 05.06.73 | Yes | 1296 | |
| 24 | A | N | 08.02.69 | Emigrated 08.02.70 | No | 365 | (e) |
| 25 | B | I | 07.03.74 | Died 30.03.74 | Yes | 23 | |

analysed as though they had emigrated alive on the day of their death.

(e) Patients who emigrate or are lost to follow-up have trial times which run up to their date of disappearance.

(f) Ignore deaths after the chosen follow-up date, and be certain that no deaths before this date have escaped your notice.

(g) It is slightly preferable to calculate trial times accurately to the nearest day, especially if mortality shortly after entry is very rapid, although weeks or even months are often sufficiently accurate units of time if computing facilities are not available. (Leap years can be dealt with most conveniently, if using a computer or calculator to derive days from dates, by altering the month and year so that January and February become months 13 and 14 in the *previous* year. The number of leap-days to be counted for a date is then the number of times 4 can be divided into the corrected year, and allowance for the irregular lengths of months need make no special allowance for February.)

Now arrange the patients in order of increasing trial time. (If there is a tie, put patients who suffered a relevant event on that day just before patients who did not.) This is done in Table IX, where the life table and extents of exposure to risk of death are calculated. The life table calculated in Table IX appears as Fig. 5.

From Table IX, we obtain:

$O_A$ = observed no. of deaths in Group A = 6
$E_A$ = extent of exposure to risk of death in Group A = 8·34
$O_B$ = observed no. of deaths in Group B = 11
$E_B$ = extent of exposure to risk of death in Group B = 8·66

(At this point of the calculation, check for arithmetic accuracy by seeing that $O_A + O_B = E_A + E_B$.)

Calculate

$$X^2 = \frac{(O_A - E_A)^2}{E_A} + \frac{(O_B - E_B)^2}{E_B} = 1{\cdot}29$$

Because we are comparing 2 groups with each other, it is appropriate to compare $X^2$ with the chi-square distribution with mean *one*. When we do so, we find that the probability of getting a value of chi-square with 1 degree of freedom as big as or bigger than 1·29 is quite substantial (about 1 in 4), which suggests that the apparent superiority of Treatment A could well have arisen by chance alone.

However, before accepting this conclusion let us first study the relevance of renal function to prognosis. This requires an extra 4 columns of numbers to be added to the right of Table IX, and these are given as Table X.

From Table X:

$O_I$ = observed no. of deaths among those with renal impairment = 7
$E_I$ = extent of exposure to risk of death among those with renal impairment = 1·60
$O_N$ = observed no. of deaths among those with normal renal function = 10
$E_N$ = extent of exposure to risk of death among those with normal renal function = 15·40

and so

$$\frac{(O_I - E_I)^2}{E_I} + \frac{(O_N - E_N)^2}{E_N} = 20{\cdot}12.$$

Since the probability of getting a value of chi-square with 1 degree of freedom as big as or bigger than 20·12 is $< 0{\cdot}001$, the tendency of those with renal impairment to die sooner cannot plausibly be merely due to chance. This indicates that we should examine the treatment differences separately among those with and without renal impairment, and this is done in Tables XI and XII.

From Table XI, among patients with impaired renal function, $O_A = 4$, $E_A = 5{\cdot}42$, $O_B = 3$, and $E_B = 1{\cdot}58$.

From Table XII, among patients with normal renal function, $O_A = 2$, $E_A = 5{\cdot}01$, $O_B = 8$, and $E_B = 4{\cdot}99$.

Combining the observed numbers and the extents of exposure to risk of death in both prognostic categories:

$O_A = 4 + 2 = 6$
$E_A = 5{\cdot}42 + 5{\cdot}01 = 10{\cdot}43$
$O_B = 3 + 8 = 11$
$E_B = 1{\cdot}58 + 4{\cdot}99 = 6{\cdot}57$

Because we have now allowed for the overwhelmingly strong effect that renal condition has on prognosis, the effect of treatment on prognosis stands out more clearly. This is because, by retrospective

TABLE IX.—*Original Data Sorted by Trial Times, with Calculation of Various Quantities to 3 Decimal Places*

| Patient number | Treatment | Renal function (I/N) | Event suffered? . = No, Y = Yes | Trial time (T) sorted | e = no. of events during Day T | r = no. at risk on Day T (*i.e.* with trial times ≥ T) | p = obs. survival proportion on day T p = 1 − e/r | Overall Life Table (product of all p's up to now) | a = no. at risk in Group A on Day T | b = no. at risk in Group B on Day T | Extents of Exposure to Risk of Event (i) in Group A = e.a/r | Extents of Exposure to Risk of Event (ii) in Group B = e.b/r |
|---|---|---|---|---|---|---|---|---|---|---|---|---|
| 1 | A | I | Y | 8 } | 2 | 25 | 0·920 | 0·920 | 12 | 13 | 0·960 | 1·040 |
| 12 | A | N | Y | 8 } | | | | | | | | |
| 13 | B | I | Y | 13 | 1 | 23 | 0·957 | 0·880 | 10 | 13 | 0·435 | 0·565 |
| 16 | B | I | Y | 18 | 1 | 22 | 0·955 | 0·840 | 10 | 12 | 0·455 | 0·545 |
| 25 | B | I | Y | 23 | 1 | 21 | 0·952 | 0·800 | 10 | 11 | 0·476 | 0·524 |
| 5 | A | I | Y | 52 | 1 | 20 | 0·950 | 0·760 | 10 | 10 | 0·500 | 0·500 |
| 8 | A | I | Y | 63 } | 2 | 19 | 0·895 | 0·680 | 9 | 10 | 0·947 | 1·053 |
| 21 | A | I | Y | 63 } | | | | | | | | |
| 11 | B | N | Y | 70 | 1 | 17 | 0·941 | 0·640 | 7 | 10 | 0·412 | 0·588 |
| 10 | B | N | Y | 76 | 1 | 16 | 0·938 | 0·600 | 7 | 9 | 0·438 | 0·562 |
| 2 | B | N | Y | 180 | 1 | 15 | 0·933 | 0·560 | 7 | 8 | 0·467 | 0·533 |
| 9 | B | N | Y | 195 | 1 | 14 | 0·929 | 0·520 | 7 | 7 | 0·500 | 0·500 |
| 20 | B | N | Y | 210 | 1 | 13 | 0·923 | 0·480 | 7 | 6 | 0·538 | 0·462 |
| 7 | A | N | Y | 220 | 1 | 12 | 0·917 | 0·440 | 7 | 5 | 0·583 | 0·417 |
| 24 | A | N | . | 365 | 0* | 11 | 1 | 0·440 | 6 | 5 | 0 | 0 |
| 3 | B | N | Y | 632 | 1 | 10 | 0·900 | 0·396 | 5 | 5 | 0·500 | 0·500 |
| 17 | B | N | Y | 700 | 1 | 9 | 0·889 | 0·352 | 5 | 4 | 0·556 | 0·444 |
| 4 | A | N | . | 852 | 0* | 8 | 1 | 0·352 | 5 | 3 | 0 | 0 |
| 23 | B | N | Y | 1296 } | 1 | 7 | 0·857 | 0·302 | 4 | 3 | 0·571 | 0·429 |
| 18 | A | N | . | 1296 } | | | | | | | | |
| 22 | A | N | . | 1328 | 0* | 5 | 1 | 0·302 | 3 | 2 | 0 | 0 |
| 19 | A | N | . | 1460 | 0* | 4 | 1 | 0·302 | 2 | 2 | 0 | 0 |
| 15 | A | N | . | 1976 | 0* | 3 | 1 | 0·302 | 1 | 2 | 0 | 0 |
| 14 | B | N | . | 1990 | 0* | 2 | 1 | 0·302 | 0 | 2 | 0 | 0 |
| 6 | B | N | . | 2240 | 0* | 1 | 1 | 0·302 | 0 | 1 | 0 | 0 |
| | | | | | | | | | | | Sum =8·338 | Sum =8·662 |

* As with days which nobody has as their trial time, so days which nobody who suffers an event has as their trial time make no difference to the life table, nor to the extent of exposure to risk, and so this table would lead to identical calculations if the asterisked lines were omitted (or were left blank from the 6th column onwards). The life table for a particular day gives the estimated probability of not suffering an event within that number of days from randomization. This life table, plotted in Fig. 5, combines Groups A and B; we could, of course, have computed separate life tables for Treatments A and B as well.

TABLE X.—*Extension of Table IX to Study the Relevance of Impaired Renal Function to Prognosis*

| | Numbers alive and at risk | | | Extent of Exposure to Risk of Death | |
|---|---|---|---|---|---|
| e, from Table IX | r, from Table IX | i = no. impaired | n = no. normal | in impaired = e.i/r | in normal = e.n/r |
| 2 | 25 | 7 | 18 | 0·560 | 1·440 |
| 1 | 23 | 6 | 17 | 0·261 | 0·739 |
| 1 | 22 | 5 | 17 | 0·227 | 0·773 |
| 1 | 21 | 4 | 17 | 0·190 | 0·810 |
| 1 | 20 | 3 | 17 | 0·150 | 0·850 |
| 2 | 19 | 2 | 17 | 0·211 | 1·789 |
| 1 | 17 | 0 | 17 | 0 | 1 |
| 1 | 16 | 0 | 16 | 0 | 1 |
| 1 | 15 | 0 | 15 | 0 | 1 |
| 1 | 14 | 0 | 14 | 0 | 1 |
| 1 | 13 | 0 | 13 | 0 | 1 |
| 0 | 12 | 0 | 12 | 0 | 1 |
| 1 | 11 | 0 | 11 | 0 | 0 |
| 1 | 10 | 0 | 10 | 0 | 1 |
| 1 | 9 | 0 | 9 | 0 | 1 |
| 0 | 8 | 0 | 8 | 0 | 0 |
| 1 | 7 | 0 | 7 | 0 | 1 |
| 0 | 6 | 0 | 6 | 0 | 0 |
| 0 | 5 | 0 | 5 | 0 | 0 |
| 0 | 4 | 0 | 4 | 0 | 0 |
| 0 | 3 | 0 | 3 | 0 | 0 |
| 0 | 2 | 0 | 2 | 0 | 0 |
| 0 | 1 | 0 | 1 | 0 | 0 |
| | | | | Sum = 1·599 | Sum = 15·401 |

stratification, our analysis now only compares like with like, instead of mixing all sorts of different patients blindly together as before. Using the above observed numbers and the extents of exposure calculated within prognostic strata, we now have:

$$X^2 = \frac{(6-10{\cdot}43)^2}{10{\cdot}43} + \frac{(11-6{\cdot}57)^2}{6{\cdot}57}$$

$$= 4{\cdot}87,$$

and referring to the chi-square distribution appropriate for a 2-group comparison (*i.e.* chi-square with 1 "degree of freedom"), we find that $0{\cdot}025 < P < 0{\cdot}05$. The difference in prognosis between the 2 groups is therefore statistically significant ($X^2 = 4{\cdot}87$, d.f. = 1, $P < 0{\cdot}05$), although only marginally so.

After adjustment for the effects of renal condition on the extent of exposure to risk of death, the relative death rate in Group A is 0·58 (6/10·43), that in Group B is 1·67 (11/6·57), and so the ratio of the death rate on Treatment A to that on Treatment B is 0·34 (0·58/1·67). In other words, the death rate observed on A is about one-third of that on B, although the vast uncertainty attached to this as an estimate of medical fact is emphasized by the fact that this extreme ratio is only just significantly different from unity!

If you have a statistician analysing your data for you, he may use your O's and E's to calculate something slightly different from your $X^2$ to compare with the chi-square distribution (Peto and Pike, 1973; Breslow. 1975—see statistical notes 7 and 8 on p. 38), However, the answers you will obtain by the simple analogy between $X^2$ and the chi-square distribution will give an adequate medical understanding of the data. As was noted on p. 20, a convenient computer program is available on request which will perform all the analyses described in Appendix 3.

APPENDIX 4.—*How to Record Data in Such a Way that it is Easy to Analyse by Computer*

(a) Computers like to read data one line at a time, each line containing up to, but not more than, 80 characters (letters, digits, blanks, dots, *etc.*). Computers like to tell what a number denotes, simply by which position it occupies in the line (*e.g.* the 43rd character), so omission of a blank can shift

**Table XI.**—*Treatment Effect among those with* Impaired *Renal Function*

| Patient number | Treatment | Event suffered? | Trial time | e = no. of events | Numbers at Risk: r = both | Numbers at Risk: a = Group A | Numbers at Risk: b = Group B | Extent of Exposure: (i) in Group A = e.a/r | Extent of Exposure: (ii) in Group B = e.b/r |
|---|---|---|---|---|---|---|---|---|---|
| 1 | A | Y | 8 | 1 | 7 | 4 | 3 | 0·571 | 0·429 |
| 13 | B | Y | 13 | 1 | 6 | 3 | 3 | 0·500 | 0·500 |
| 16 | B | Y | 18 | 1 | 5 | 3 | 2 | 0·600 | 0·400 |
| 25 | B | Y | 23 | 1 | 4 | 3 | 1 | 0·750 | 0·250 |
| 5 | A | Y | 52 | 1 | 3 | 3 | 0 | 1·000 | 0 |
| 8 | A | Y | 63 | 2 | 2 | 2 | 0 | 2·000 | 0 |
| 21 | A | Y | 63 | | | | | | |
| | | | | | | | | Sum = 5·421 | Sum = 1·579 |

**Table XII.**—*Treatment Effect among those with* Normal *Renal Function*

| Patient number | Treatment | Event suffered? | Trial time | e = no. of events | Numbers at Risk: r = both | Numbers at Risk: a = Group A | Numbers at Risk: b = Group B | Extent of Exposure: (i) in Group A = e.a/r | Extent of Exposure: (ii) in Group B = e.b/r |
|---|---|---|---|---|---|---|---|---|---|
| 12 | A | Y | 8 | 1 | 18 | 8 | 10 | 0·444 | 0·556 |
| 11 | B | Y | 70 | 1 | 17 | 7 | 10 | 0·412 | 0·588 |
| 10 | B | Y | 76 | 1 | 16 | 7 | 9 | 0·438 | 0·562 |
| 2 | B | Y | 180 | 1 | 15 | 7 | 8 | 0·467 | 0·533 |
| 9 | B | Y | 195 | 1 | 14 | 7 | 7 | 0·500 | 0·500 |
| 20 | B | Y | 210 | 1 | 13 | 7 | 6 | 0·538 | 0·462 |
| 7 | A | Y | 220 | 1 | 12 | 7 | 5 | 0·583 | 0·417 |
| 24 | A | . | 365 | 0 | 11 | 6 | 5 | 0 | 0 |
| 3 | B | Y | 632 | 1 | 10 | 5 | 5 | 0·500 | 0·500 |
| 17 | B | Y | 700 | 1 | 9 | 5 | 4 | 0·556 | 0·444 |
| 4 | A | . | 852 | 0 | 8 | 5 | 3 | 0 | 0 |
| 23 | B | Y | 1296 | 1 | 7 | 4 | 3 | 0·571 | 0·429 |
| 18 | A | . | 1296 | | | | | | |
| 22 | A | . | 1328 | 0 | 5 | 3 | 2 | 0 | 0 |
| 19 | A | . | 1460 | 0 | 4 | 2 | 2 | 0 | 0 |
| 15 | A | . | 1976 | 0 | 3 | 1 | 2 | 0 | 0 |
| 14 | B | . | 1990 | 0 | 2 | 0 | 2 | 0 | 0 |
| 6 | B | . | 2240 | 0 | 1 | 0 | 1 | 0 | 0 |
| | | | | | | | | Sum = 5·009 | Sum = 4·991 |

all subsequent numbers one space leftwards and cause chaos. It is not humanly possible to feed in data as carefully as this, unless you first write out the data on a coding form. Fig. 7 illustrates a coding form used in a recent MRC trial. Although the coding form illustrated was designed to be completed by the physician referring the patient, it is usually preferable to ask the physician to complete a form designed with his convenience and understanding chiefly in mind, and to copy this on to a computer coding form at the trial centre. This keeps him as co-operative as possible, and forces you to check that all requisite information has been supplied. (Immediate queries of missing or doubtful information are better than queries during the statistical analysis a year or two later.)

(b) On the coding form, there are a given number of boxes for each item of information, and the information is written in, one character per box. Although the boxes are all on different lines on the coding form, before they are fed into the computer, they will all be concatenated into a single line, giving all the data for that one patient (unless that makes more than 80 boxes, in which case the line will be cut up at certain points into 2 or more lines per patient, each 80 or less characters in length).

(c) Apart from the boxes reserved for the patient's name, in which you will write a mixture of letters and blanks, try to use nothing but digits and blanks—no letters, and no dots. This will make the programmer's task a little easier. For example, if you want to record a patient's sex, have a box for doing so, but adopt the coding convention 1 for male and 2 for female instead of entering M or F. Also, if you want to record a number with a decimal part, leave out the dot and just give the figures (115 for 11·5 and 110 for 11·0, for example). Everything that has a decimal part must be given to the same number of decimal places, even if the decimal part is zero.

(d) Computers do not like to distinguish between blank and zero. This has two consequences. Firstly, if nothing is written for a piece of information, the computer will read it as though zero were written there. (If, therefore, zero is a possible value, it is usually better to have an impossible number that can be entered to denote " no data ", and when defining how replies about something are to be recorded as a number, it is better not to have zero as one of the codes.) Secondly, if someone has a pair of boxes in which to enter the number 9, he must write it in the right-hand box: if he wrote it in the left-hand box of the pair, the computer would take the number written in that pair of boxes as being 90!

(e) Never *under*estimate the number of characters required for recording something. For example, if a number might possibly, just for one patient in the whole trial, run into 3

| | |
|---|---|
| Surname<br>(first 10 letters only, if long) | I R V I N G _ _ _ _ |
| Sex (1 M, 2 F) | 1 |
| Date randomized (d, m, y) | _ 6 _ 8 7 6 |
| Hb at presentation<br>(g/100 to one d.p., omitting the point) | 1 5 0 |
| Leucocytes<br>(000/μl: enter 999 if not known) | _ _ 5 |
| | etc. |

(So far, 23 characters have been used; this form actually ran to 68 characters in all.)

FIG. 7.—Part of a coding form.

digits (*e.g.* age), then allow 3 boxes for it. It does not matter if, in the event, the left-hand box is never used.

APPENDIX 5.—*Testing for a Trend in Prognosis with Respect to an Explanatory Variable*

Frequently, if a group of patients is divided into more than 2 subgroups with respect to an explanatory variable, these subgroups have a natural order (*e.g.* low, medium, high) and can be numbered 1, 2, 3 . . . in a non-arbitrary way. It is then usually more sensitive, if we need to know whether there is any relationship of that explanatory variable to prognosis, to ask whether there is a statistically significant tendency for a *trend* in prognosis to exist as we go from Group 1 to Group 2 to Group 3 (and on, if there are more than 3 groups), instead of asking if there is statistically significant *heterogeneity* (by calculation of $X^2$, as on page 31). There are a few situations, however, where there could be real heterogeneity but no real trend; for example, average patients might fare better than patients who are extreme in either direction, up or down. The best general policy, when examining the relevance to prognosis of an explanatory variable which is split into 3 or more naturally ordered subgroups, should therefore be to calculate *both* the test for trend (described below) and the $X^2$ test for heterogeneity. However, the *P*-value ultimately used to help infer whether this explanatory variable is at all related to prognosis should nearly always be based on the test for trend, rather than on that for heterogeneity, even if the statistical significance of the heterogeneity test is slightly more extreme than that of the test for trend.

*Computational details.*—The test for trend involves these steps:

(a) Divide the patients into subgroups with respect to the explanatory variable.The choice of the number of subgroups is not usually critical, except that if it is small it is slightly preferable for it to be odd (*e.g.* 3 or 5) rather than even. It is usually best to aim to have roughly similar numbers (to within a doubling) in each subgroup, although this is not essential, and if medical considerations suggest particular natural groupings, especially of a non-continuous explanatory variable such as disease stage, these should be adopted.

(b) Give each subgroup a number, starting at 1 and working upwards in natural order (*e.g.* low urea = 1, medium urea = 2, high urea = 3).

(c) For each subgroup count O, the observed number of events, and calculate E, the extent of exposure to risk of such events, according to the methods described in Section 19. Add up the O's to obtain Osum and the E's to obtain Esum, and check that, apart from rounding errors, Osum = Esum. If not, there is an arithmetical error in the derivation of the O's or the E's. (Note that Esum is simply the total number of patients in the whole trial who have suffered an event.)

(d) Calculate $X^2$, the test statistic for heterogeneity, as the sum of $(O-E)^2/E$, as in Section 20.

(e) In each subgroup we now have $n$, the subgroup number, and the logrank O and E for that subgroup. Calculate, within each subgroup:

$$A = n(O-E)$$
$$B = nE$$
$$C = n^2E$$

(f) Add up all the A's in the different subgroups to obtain " Asum ". Analogously obtain " Bsum " and " Csum ".

(g) Calculate V where V = Csum—(Bsum × Bsum/Esum) (see statistical note 9 on p. 38). Finally, calculate T, the test statistic for trend, where

$$T = \text{Asum} \times \text{Asum}/V.$$

(If T is negative or exceeds $X^2$ you have made an arithmetical error somewhere: if Osum and Esum are equal after step c, there must be an error in step d, e, f or g.)

(h) Obtain a *P*-value by using an analogy which exists between the behaviour of T if the explanatory variable is in fact irrelevant and the behaviour of one of the standard distributions in statistics, called " chi-square on 1 degree of freedom " (see page 10). This implies that if the explanatory variable is irrelevant (so there is no real trend), then

T is zero or positive, and would be expected to be around unity

T has an approximately $\frac{1}{3}$ chance of exceeding unity

T has an approximately 10% chance of exceeding 2·71

T has an approximately 5% chance of exceeding 3·84
T has an approximately $2\frac{1}{2}$% chance of exceeding 5·02
T has an approximately 1% chance of exceeding 6·63
T has an approximately $\frac{1}{2}$% chance of exceeding 7·88
T has an approximately 0·001 chance of exceeding 10·83.

For example, if we computed T = 4·32 in a particular case, we might insert, in our published account of the data, "Chi-square test for trend yielded 4·32; d.f. = 1; $P < 0{\cdot}05$."

Example: data from Table V are displayed in Table XIII, with derivation from them of the requisite quantities.

$$\text{Now V} = 567{\cdot}52 - 320{\cdot}28 \times 320{\cdot}28/213{\cdot}00 = 85{\cdot}93$$

$$\text{and T} = 79{\cdot}72 \times 79{\cdot}72/85{\cdot}93 = 73{\cdot}96$$

(Check: Osum $\simeq$ Esum and zero $\leq T \leq X^2$.)

If initial urea were irrelevant to prognosis, the probability that, by chance alone, T would exceed 10·83, is approximately 0·001, so the probability in this case that T should equal or exceed 73·96 is much less than 0·001. We would therefore write "Chi-square test for trend yielded 73·96; d.f. = 1; $P < 0{\cdot}001$."

Incidentally, in a test for trend between just 2 groups of patients, so that $n = 2$, T will necessarily just equal $X^2$.

### STATISTICAL NOTES

As in Part I, these are collected together so that they can be completely ignored by the non-statistical reader. The statistical methods recommended in this paper are developed in Kaplan and Meier (1958), Mantel (1966), Peto (1972), Cox (1972), and Peto and Pike (1973), and are reviewed by Breslow (1975).

STATISTICAL NOTE 6.—(From p. 6). If the life table at a particular time after randomization equals L, and there are then N patients still at risk, the standard error of L at that time is approximately $L\sqrt{((1-L)/N)}$. (Example: suppose that at one year after randomization there are 20 survivors still being observed, and the life table estimate of the chance of surviving one full year from randomization is 0·2. The estimated standard error of the life table at one year would then be $0{\cdot}2 \times \sqrt{(0{\cdot}8/20)}$, which is 0·04.)

The justification for this formula is that there must initially have been at least N/L patients for N to remain when the life table equals L. If originally there were exactly N/L, binomial theory yields the standard error estimate $L\sqrt{((1-L)/N)}$. If there were more than N/L originally, the life table will be somewhat more accurate than this, depending on the trial times of the surviving patients. This estimate is therefore usually conservative, although the actual degree of conservatism is often surprisingly slight, and is counterbalanced by the fact that the formula deals appropriately with the increasing uncertainty that should properly be expected as one goes along the long flat region with which many life tables finish. The Greenwood standard error estimate (Kaplan and Meier, 1958) is less immediately computed, and has the disadvantage that in such life table tails, which are often the regions of greatest medical interest (and the source of most mistakes), the standard error may be grossly underestimated. Whichever estimate is preferred, the trial times of the surviving patients should somehow be indicated with the plotted life table as in Figs. 3, 4 and 5.

TABLE XIII.—*Test for Trend in Prognosis with Respect to Initial Blood Urea Among Patients Entered into the First MRC Myelomatosis Trial (From Table V)*

| Initial urea (mg/100 ml blood) | Group number, *n* | O, observed no. of deaths | E, extent of exposure to risk of death | $\frac{(O-E)^2}{E}$ | A = *n* (O−E) | B = *n* E | C = $n^2$E |
|---|---|---|---|---|---|---|---|
| 0–39 | 1 | 79 | 122·06 | 15·19 | −43·06 | 122·06 | 122·06 |
| 40–79 | 2 | 81 | 74·60 | 0·55 | 12·80 | 149·20 | 298·40 |
| 80– | 3 | 53 | 16·34 | 82·25 | 109·98 | 49·02 | 147·06 |
| Totals | | 213 | 213·00 | 97·99 | 79·72 | 320·28 | 567·52 |
| Names of totals: | | Osum | Esum | $X^2$ | Asum | Bsum | Csum |

STATISTICAL NOTE 7.—(From p. 11). For a 2-group comparison, the variance, V, of $(O_A - E_A)$ can be computed. It is the same as the variance of $(O_B - E_B)$, of course, and is obtained by arguing that, for those alive and observed in the trial on each particular day, a $2 \times 2$ table giving group membership (A or B) and fate that day (died or not) can be constructed. The variance of the difference between observed and expected for the number of Group A deaths in each such table can be calculated, and since $O_A - E_A$ is the sum of all such differences V is the sum of all such variances. On a day when there are $a$ A-patients and $b$ B-patients at risk and the observed survival rate among both groups of patients combined is $p$, the contribution to the overall variance V will be $p(1-p)ab/(a+b-1)$.

Finally, $(O_A - E_A)^2/V$ (or, if a continuity correction is preferred,* $(|O_A - E_A| - \frac{1}{2})^2/V$) is referred to tables of chi-square with one degree of freedom. If the continuity correction of $\frac{1}{2}$ is not used, this is necessarily greater than or equal to $X^2$. If more than 2 groups are to be compared a variance/covariance matrix from each day must be accumulated to give the overall variance/covariance matrix for the vector of the $(O-E)$'s in each group, but the principle is similar. Details are given by Peto and Pike (1973) and an example is given in Appendix 3.

There is a strong connection between these methods and those of Cox (1972) for comparing 2 groups. Cox uses $\beta$ to denote the log of the ratio of the hazard functions in the 2 treatment groups. He then derives a log-likelihood $L(\beta)$ and uses the statistic $L'(0)$ to test $\beta = 0$ noting from likelihood theory that its variance must be approximately $-L''(0)$. In the absence of tied ranks $L'(0)$ and $-L''(0)$ are the logrank $(O-E)$ and its variance V, giving a deeper justification to logrank methods.

STATISTICAL NOTE 8.—(From p. 33). In this particular example, where $O_A = 6$ and $E_A = 10{\cdot}43$ one might use the methods described in the statistical note 7 to compute $V = \text{variance of } (O_A - E_A) = 3{\cdot}39$, leading to a chi-square of $4{\cdot}43^2/3{\cdot}39 = 5{\cdot}79$ without a continuity correction, or, if a continuity correction is preferred, $(4{\cdot}43 - \frac{1}{2})^2/3{\cdot}39 = 4{\cdot}56$.

STATISTICAL NOTE 9.—(From p. 36). The approximation is being made that under the null hypothesis Asum has mean zero (which is exactly true) and variance V (which is not). The exact variance, $V_{exact}$, of Asum may be obtained by noting that, if $x_i$ denotes the

* Continuity correction. With more than 2 groups, a continuity correction is definitely unwanted, but there is no uniform practice of using or not using a continuity correction when comparing 2 groups by the logrank test, and the reasons why are quite interesting. Two fundamentally different methods may be available for deriving the $P$-value from the calculated values of O and E in each group, the permutational method (Peto, 1972) or the conditional method (Mantel, 1966). If there are initially $a$ Group A subjects and $b$ Group B subjects, then both methods calculate a $P$-value *given* the times from randomization at which deaths occur and *given* the durations of follow-up of those who do not die. The permutational $P$-value, $P_{perm}$, is the probability that if $a$ of the $a + b$ patients were selected at random and taken as Group A, the remainder being taken as Group B, a value of $(O - E)$ as extreme as or more extreme than that actually observed would then be generated. The conditional argument considers the information in the data to be equivalent to that in a hypothetical set of independent $2 \times 2$ tables, one for each post-randomization day with margins equal to the margins of the $2 \times 2$ tables actually observed relating death on that day to group membership among those still at risk on that day. The conditional probability, $P_{cond}$, is then the probability that the value of $(O - E)$ obtained by combining a set of such $2 \times 2$ tables in the usual way would be as extreme as or more extreme than the observed $(O - E)$.

The permutational argument is usually applicable when two groups being compared have been separated by randomization, and is then preferable because $P_{perm}$ will usually, although not always, be smaller than $P_{cond}$. The variance of $(O - E)$ under the permutational argument is approximately V, and since there are usually many permutations that would lead to values of $(O - E)$ very close to, but not equal to, the value of $(O - E)$ actually obtained, $P_{perm}$ is usually better estimated if a continuity correction is *not* used.

The conditional argument lacks a little of the efficiency of the permutational argument, but leads more naturally into Cox's (1972) methods, and into the study of treatment effects in particular time-periods. However, since it leads us, albeit slightly artificially, to regard E as fixed and O as an *integer-valued* random variable with variance exactly V, $P_{cond}$ is usually better estimated if a continuity correction *is* used.

The conditional argument is always valid, but the permutational argument is sometimes not (*e.g.* if we compare survival among 2 groups of patients which have on average been followed up for different lengths of time). Nevertheless, when comparing the efficiency of the logrank test with that of alternative statistica tests for detecting differences between randomized groups, the efficiency of the permutational argument should be used.

value of (O − E) in the $i^{th}$ subgroup, Asum = $\Sigma i x_i$ and the $V_{exact}$ is $\Sigma\Sigma ij c_{ij}$, where summation is over all groups from first to last inclusive and $c_{ij}$, the covariance of $x_i$ with $x_j$, is given by Peto and Pike (1973). A preferable test for trend, albeit one which is not accessible without statistical expertise, is then to take $Asum^2/V_{exact}$ (or, if a continuity correction is preferred, $(|Asum| - 0{\cdot}5)^2/V_{exact}$) as chi-square with 1 d.f. There is a strong connection between this recommended test for trend and the methods developed by Cox (1972). These methods test for trend by attempting to relate the log hazard function linearly to the subgroup number by a parameter $\beta$ and then testing $\beta = 0$ by examining the log-likelihood function, $L(\beta)$. Since the quantity Asum equals Cox's $L'(0)$, and in the absence of tied ranks the quantity $V_{exact}$ equals Cox's $L''(0)$, it is therefore of full asymptotic efficiency in a reasonable class of models to base a test for trend on Asum.